# BrainWAVE: A Flexible Method for Noninvasive Stimulation of Brain Rhythms across Species

**DOI:** 10.1523/ENEURO.0257-22.2022

**Published:** 2023-02-23

**Authors:** Matthew K. Attokaren, Nuri Jeong, Lou Blanpain, Abigail L. Paulson, Kristie M. Garza, Ben Borron, Michael Walelign, Jon Willie, Annabelle C. Singer

**Affiliations:** 1 Coulter Department of Biomedical Engineering, Georgia Institute of Technology and Emory University, Atlanta, GA 30322; 2 Neuroscience Graduate Program, Graduate Division of Biological and Biomedical Sciences, Emory University, Atlanta, GA 30322; 3 Neurosurgery, Biomedical Engineering, Psychiatry, Neuroscience and Neurology, Washington University, St Louis, MO 63110

**Keywords:** brain oscillations, brain stimulation, entrainment, flicker, noninvasive deep brain modulation, sensory stimulation

## Abstract

Rhythmic neural activity, which coordinates brain regions and neurons to achieve multiple brain functions, is impaired in many diseases. Despite the therapeutic potential of driving brain rhythms, methods to noninvasively target deep brain regions are limited. Accordingly, we recently introduced a noninvasive stimulation approach using flickering lights and sounds (“flicker”). Flicker drives rhythmic activity in deep and superficial brain regions. Gamma flicker spurs immune function, clears pathogens, and rescues memory performance in mice with amyloid pathology. Here, we present substantial improvements to this approach that is flexible, user-friendly, and generalizable across multiple experimental settings and species. We present novel open-source methods for flicker stimulation across rodents and humans. We demonstrate rapid, cross-species induction of rhythmic activity without behavioral confounds in multiple settings from electrophysiology to neuroimaging. This flicker approach provides an exceptional opportunity to discover the therapeutic effects of brain rhythms across scales and species.

## Visual Abstract

**Figure:**
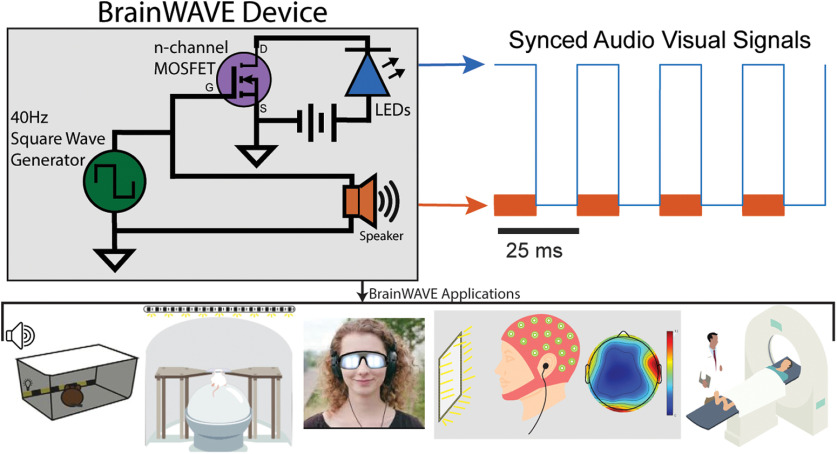

## Significance Statement

Despite many studies showing abnormal brain rhythms in multiple diseases, limited means to target deep brain regions noninvasively has restricted the therapeutic potential of driving brain rhythms. Accordingly, we developed a noninvasive millisecond precise sensory stimulation to drive brain rhythms. Here, we introduce for the first time newly developed open-source software and instructions for building, testing, debugging, and using BrainWAVE (Brain Wide-spectrum Audio/Visual Exposure) stimulation. We demonstrate BrainWAVE stimulation across multiple species and different experimental settings. These methods constitute a customizable, open-source, accessible, and noninvasive technology that stimulates brain oscillations to causally test how rhythmic brain activity impacts brain function.

## Introduction

Neural oscillations, rhythmic patterns of activity in the brain, have been observed across many species and extensively investigated in studies of sensory and cognitive processing. Studies of humans and animal models of disease have uncovered neural deficits of different frequencies in multiple brain regions (Herrmann and Demiralp, 2005; Uhlhaas and Singer, 2006; Worrell et al., 2008; Verret et al., 2012; J. Wang et al., 2013; Schneider et al., 2014; Shahriari et al., 2016; Tamura et al., 2017; Solís-Vivanco et al., 2018; Prince et al., 2021). In particular, we and others have found reduced power of endogenous γ-frequency oscillations in mouse models of Alzheimer’s disease (Verret et al., 2012; Goutagny et al., 2013; Iaccarino et al., 2016; Mably et al., 2017; Martorell et al., 2019). We previously showed that enhancing γ neural activity using noninvasive rhythmic sensory stimulation (hereinafter referred to as “flicker”), specifically lights and/or sounds turning on and off at 40 Hz, reduced Alzheimer’s pathology, recruited immune cells, and improved memory performance in mice (Iaccarino et al., 2016; Adaikkan et al., 2019; Martorell et al., 2019). These studies highlight the potential therapeutic benefits of modulating neural oscillations using noninvasive sensory stimulation. Indeed, a growing number of studies have used flicker to ameliorate pathology beyond Alzheimer’s disease. For example, 30- to 50-Hz light flicker was protective against neurons in a cerebral ischemia model (Zheng et al., 2020). Other studies have found flicker to be effective in correcting circadian rhythms (Yao et al., 2020; Chan et al., 2021). Furthermore, this sensory stimulation is useful for studying how rhythmic neural activity affects brain function including immune cells and signals (Iaccarino et al., 2016; Martorell et al., 2019; Garza et al., 2020; He et al., 2021; Venturino et al., 2021). These findings demonstrate the general applicability of flicker stimulation as a promising means to treat multiple disorders and diseases (Jones et al., 2019; Park et al., 2020; Huang et al., 2021; Shi et al., 2021).

Flicker stimulation has significant advantages over existing methods of neuromodulation. First, flicker successfully produces reliable modulation in multiple species, including rodents and humans, in multiple brain regions, including difficult-to-target areas such as the hippocampus (Iaccarino et al., 2016; Adaikkan et al., 2019; Martorell et al., 2019; He et al., 2021; Quon et al., 2021). The ability to reach deeper brain regions beyond superficial sensory areas is particularly exciting because many such brain regions are important for cognition and affected by disease but difficult to modulate noninvasively (Qin et al., 2018; Lozano et al., 2019; Spagnolo et al., 2019; Hescham et al., 2020). Second, sensory flicker has been shown to alter neuroimmune signaling in mice and humans (Iaccarino et al., 2016; Adaikkan et al., 2019; Martorell et al., 2019; Garza et al., 2020; He et al., 2021; Venturino et al., 2021). Deficits in neuroimmune function are prevalent in many diseases, but traditional invasive techniques to manipulate neural or immune activity cause immune responses themselves thereby limiting their use or interpretation in the study of neuroimmune function (Chung et al., 2015; Hong et al., 2016; Wohleb et al., 2016; Hickman et al., 2018). Third, flicker is an attractive option for chronic at-home therapy in humans. This inexpensive flicker device is widely accessible to researchers and clinicians. Finally, flicker has limited risk and potential side effects in studies to date and offers an attractive model for individualized therapy programs.

Despite these advantages, earlier versions of flicker devices had limitations and room for improvement. Previous flicker stimulation devices used separate systems for audio and visual stimulation without the ability to synchronize the two signals. Furthermore, prior work did not describe methods to test and debug multimodal flicker. These early devices also required extensive knowledge of coding and circuitry to build and operate. As a result, these devices were not user-friendly or fully optimized for clinical and research use. Early users could not easily and quickly adjust brightness and volume based on participant comfort and study goals. Furthermore, previous visual flicker systems could not be used for magnetic resonance imaging (MRI) studies because they were either not MR-compatible or were not bright enough to sufficiently illuminate the field of view of a participant in the scanner bore from a safe distance. Furthermore, there was no established protocol for blinding, which is especially tricky when the intervention is visible to the experimenter and easily distinguished from control conditions. Earlier studies also did not describe the experience or potential side effects of flicker in healthy subjects, which is important to consider in how readily usable this stimulation is in a variety of participants.

For these reasons, we developed an easy-to-build, modular, and customizable BrainWAVE (Brain Wide-spectrum Audio/Visual Exposure) Stimulator to modulate neural activity across multiple species and experimental settings. Given the general utility of this device, here we introduce for the first time open-source software and instructions for hardware assembly, testing, and debugging. We developed a user-friendly graphical user interface (GUI) to easily control and adjust flicker during experiments without programming. We demonstrate the feasibility, safety, and effectiveness of our newly optimized methods for use in both clinical and preclinical research. We extend our previously published work by detailing how to implement and troubleshoot flicker stimulation across multiple species and different experimental settings, including intracranial recordings from humans and mice, behavioral assays in mice, and assays of side effects in humans. We have also developed methods for minimizing signal interference during simultaneous audio and visual flicker with human electroencephalography (EEG) recordings or MRI scans while participants engage in behavior tasks. Additionally, we outline how to design and conduct a blinded flicker study and discuss considerations for human and animal experiments involving flicker that we hope will aid future research. These methods constitute a customizable, accessible, and noninvasive technology that stimulates brain oscillations to causally test how rhythmic brain activity impacts brain function.

## Materials and Methods

### Device design

**Figure 1.**
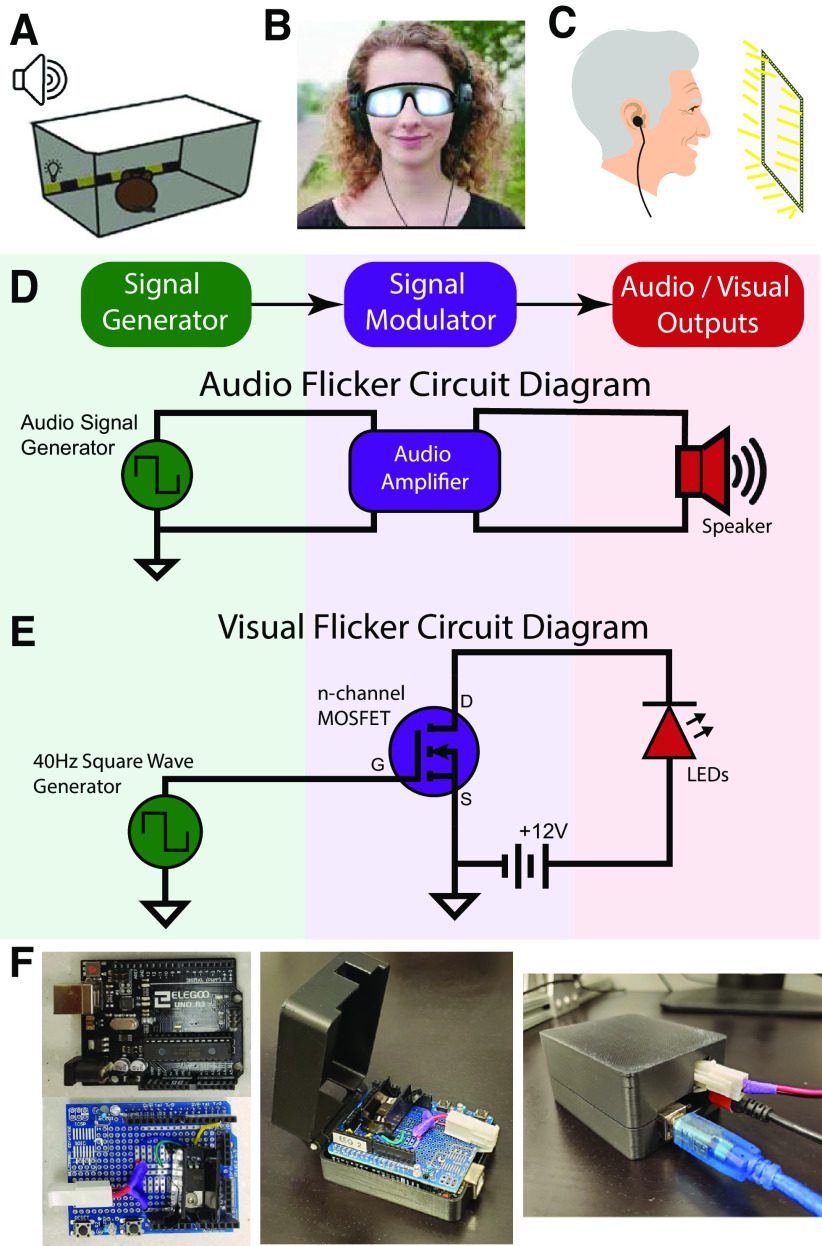
BrainWAVE circuit design. ***A***, Flicker presentation for mice in a clear enclosure with a strip of light-emitting diodes (LEDs) and a speaker. ***B***, Flicker presentation for humans with LED-lined goggles and headphones. ***C***, Flicker presentation for humans with an LED-frame surrounding a computer monitor and earbuds. ***D***, A circuit diagram of an audio flicker circuit with an audio amplifier to allow for volume adjustments. ***E***, A circuit diagram of an LED visual flicker circuit with a metal–oxide–semiconductor field-effect transistor (MOSFET) to allow a 12-V voltage source to power the LEDs. ***F***, Left, An Arduino Uno microcontroller (top) and Arduino shield (bottom). A MOSFET and wires are soldered on the Arduino shield. Middle, The Arduino shield fits on top of the Arduino Uno and both sit in a custom 3D-printed case. Right, The Arduino and BrainWAVE stimulator circuit shield are enclosed in a 3D-printed case. Ports in the case allow a USB Type-B cable, an LED cable, and a power cable to plug into the Arduino and shield. See Extended Data Table 1-1 for a list of BrainWAVE stimulator parts. See Extended Data Figure 1-1 for a physical diagram of an Arduio-Visual BrainWAVE circuit. See Extended Data 2 for detailed instructions on how to assemble the device.

The hardware components of each type of BrainWAVE stimulator were selected to suit many different types of subjects (animals/rodents, patients, healthy humans) and different types of studies (EEG, MRI, behavior, electrophysiological recordings, intracranial EEG, etc.). For mouse studies, we designed and built a BrainWAVE stimulator using a strip of LEDs (light-emitting diodes) and a speaker to administer sensory stimulation to mice housed within a cage (Fig. 1*A*) to assess behavioral and immunologic effects. To deliver flicker stimulation to humans, we developed devices that consisted of headphones or earbuds and LED goggles or an LED frame (Fig. 1*B*,*C*). For audio stimulation during intracranial recordings or scalp EEG recordings small earbuds were advantageous compared with headphones with a headband since the headband interfered with electrodes. When incorporating flicker with computer-based behavioral tasks, we used an LED frame placed around a computer monitor (Fig. 1*C*).

10.1523/ENEURO.0257-22.2022.f1-1 Extended Data Figure 1-1 Diagram of audio-visual BrainWAVE circuit. The audio and visual signal generators (green), an Arduino Uno, and a data acquisition device (DAQ) supply signals to the signal modulators (purple), an audio amplifier and a MOSFET, which then control the outputs (red) for this circuit, a speaker and LED strip lights. Eight 1.5-V AA batteries supply power to a strip of LEDs. Download Figure 1-1, TIF file.

10.1523/ENEURO.0257-22.2022.t1-1 Extended Data Table 1-1 Parts list. Components for signal generation, modulation, and sensory stimulation are needed to assemble and BrainWAVE stimulator. This table lists components that can be used and their estimated price. Download Table 1-1, DOCX file.

Each BrainWAVE stimulator consisted of three types of hardware components: signal generators, signal modulators, and the audio and visual outputs (Fig. 1*D*,*E*; Extended Data Table 1-1). These three components were assembled to create either two independent audio and visual BrainWAVE stimulator circuits or one combined circuit (Fig. 1*D–F*; Extended Data Fig. 1-1). A signal generator produced the signal dictating the on/off timing of audio and visual outputs before sending it to a signal modulator. The signal modulator amplified or attenuated the flicker control signal to adjust the intensity (e.g., brightness or volume) of the stimulation. The modulated signal was then sent to audio and visual output components, specifically the lights and speakers converting the electrical signals into visible and audible sensory stimuli.

For signal generation, we used a microcontroller (e.g., an Arduino) or digital acquisition (DAQ) hardware to create the audio and visual control signals (Fig. 1*D*,*F*; Extended Data Fig. 1-1), although other signal generators with submillisecond precision may also be used. When selecting a signal generator, the advantages and disadvantages of an Arduino or DAQ were considered in regard to the temporal precision required for a particular experiment. A DAQ and PC system offers a higher sampling rate and better temporal precision (e.g., 400,000 samples per second for a NI-DAQ USB-6212 vs ∼9600-Hz sampling rate for an Arduino Uno). Higher precision was required when aligning flicker stimulation to other signals with high temporal resolution, like electrophysiological signals in scalp EEG, intracranial EEG, or depth electrode recordings. If high sampling rate and precision were not necessary, then flicker signals were generated effectively and more inexpensively using an Arduino microcontroller (less than $25). Arduino and DAQ signal generators were programmed with custom code (see Extended Data 1) in Arduino IDE software or MATLAB, respectively. For our studies, this code was either uploaded to an Arduino Uno (for an Arduino BrainWAVE stimulator) from a PC or was run in MATLAB on a PC that sent signals to a NI-DAQ USB-6212 (for a DAQ BrainWAVE stimulator).

Next, the generated output signal was typically amplified or attenuated via a signal modulator to produce the desired level of brightness or volume for a particular experiment. An audio amplifier (Fig. 1*D*) was used to increase or decrease the volume of the audio signal before being sent to a speaker or earbuds. Visual signal modulation was achieved with a simple MOSFET circuit to power LEDs with a higher voltage source (Fig. 1*E*,*F*) because the output voltages of the signal generators were too low for some light sources. Arduino-generated visual signals were sent from the Arduino to a MOSFET circuit built on an Arduino shield and then the modified signals were sent to the visual outputs. We adjusted the brightness of the LEDs by installing a dimmer switch between the MOSFET circuit and the light source (see Extended Data 2 for detailed instructions on how to assemble a BrainWAVE stimulator). Auditory signals were similarly sent from the Arduino through an audio amplifier and then to the audio outputs. The ability to alter the level of audio and visual stimulus intensity was important especially in studies involving human subjects to ensure participant comfort and tolerance. For flexible adjustment of stimulus parameters, we developed a user-friendly GUI tool in MATLAB (software and associated code in the Extended Data 1).

A 3D-printed case was used to protect the Arduino and circuit (Fig. 1*F*). Instead of the Arduino shield, circuits may be produced via a printed circuit board (PCB) or breadboard (Extended Data Fig. 1-1). Using a PCB simplifies the assembly process, reduces the footprint of the circuit, and makes a more reliable circuit. To prevent electrical noise in electrophysiology or EEG recordings, the BrainWAVE stimulator was shielded by placing it in a metal-lined box. Testing for electrical artifacts was performed before recording.

### BrainWAVE stimulator code and software

**Figure 2.**
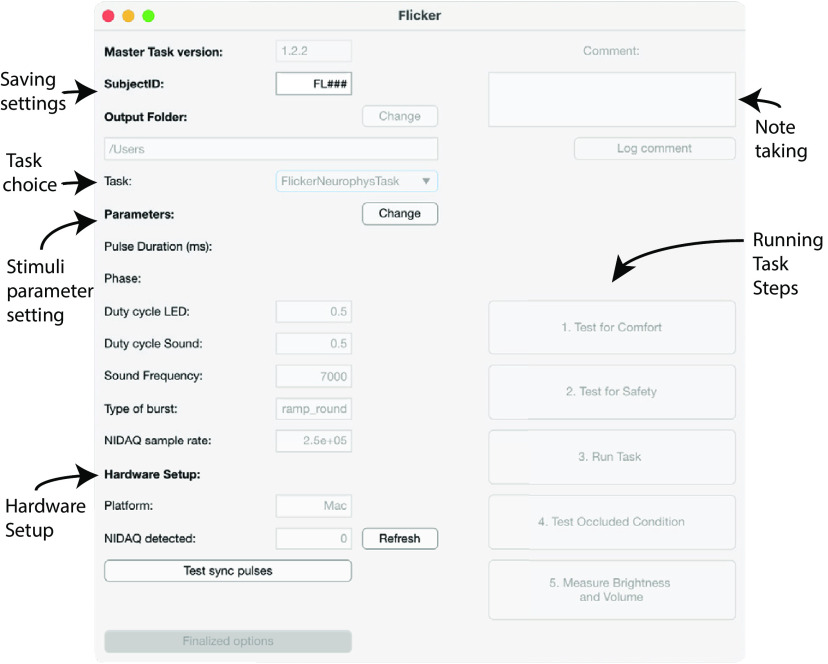
A user-friendly BrainWAVE graphical user interface (GUI) to perform many types of experiments. We developed a user-friendly application to run a variety of experiments involving visual and/or auditory stimulation. Preprogramed tasks are found under the task dropdown menu and include four different tasks. First, a classical flicker task, with exposure to 5.5 Hz (θ-like), 40 Hz (γ-like), 80 Hz, and random nonperiodic flicker at visual, audiovisual and auditory modalities. Second, a flicker duration task, exposing subjects to a given modality and frequency of flicker for minutes at a time. Third, a flicker frequency task, which allows exposing subjects to up to 26 different frequencies of flicker of a given modality. Fourth, a single pulse evoked potential task, where subjects are exposed to single visual, audiovisual and auditory 12.5-ms pulses. The stimuli parameters are set in entry boxes for stimulus duty cycle and tone (sound frequency). The comments box is used to write and save time-stamped experiment notes during the experiment. Developed for testing in human participants, each task includes tests for comfort to determine the optimal brightness and volume of the stimuli that are comfortable to the subject (adjusted on the device), tests for safety to determine whether the intended flicker stimuli induce adverse events, experimental tasks, control occluded condition (where subjects wear a sleep mask and earplugs), and measures of brightness and volume used. See Extended Data 2, BrainWAVE stimulator guide, for instructions on how to set-up and run an experiment using the BrainWAVE GUI.

Custom software controlled the signals generated by the microcontroller or DAQ. For Arduinos, code was written in Arduino IDE and uploaded to an Arduino from a Windows 10 PC. Arduino code runs automatically whenever the Arduino is supplied with power (either from a PC via USB cable or from a wall power adapter) regardless of whether the Arduino is connected to the PC. For a DAQ BrainWAVE stimulator, the DAQ was first connected to a PC with MATLAB using the data acquisition toolbox (see National Instruments for further instructions for NIDAQs). The DAQ BrainWAVE stimulator was controlled using MATLAB software and unlike an Arduino, the DAQ typically must be connected to a PC while in use. While running the DAQ system, the signals generated in MATLAB were sent from the PC to the DAQ, which in turn sent the signals to the signal amplification/attenuation components and then the output components. We developed a user-friendly application (Fig. 2) to run a variety of experiments involving visual and/or auditory stimulation. All chosen experiment details, and timing of trials, are saved in a MATLAB structure for offline data processing. Code used for Arduino and NI-DAQ BrainWAVE stimulators is found on GitHub.

### Code accessibility

The code/software described in the paper is freely available online at https://github.com/singerlabgt/BrainWAVE. The code is available as Extended Data 1.

10.1523/ENEURO.0257-22.2022.ed1 Extended Data 1 BrainWAVE stimulator code. These files contain code to generate and play flicker sensory stimulation with an Arduino Uno or NIDAQ BrainWAVE stimulator device. Download Extended Data 1, ZIP file.

10.1523/ENEURO.0257-22.2022.ed2 Extended Data 2 BrainWAVE stimulator guide. This guide provides more information on how to assemble a BrainWAVE device and set-up the software to produce the stimulation. The guide also provides information on how to measure the stimulation and troubleshooting tips. Download Extended Data 2, RTF file.

### Measuring BrainWAVE outputs

**Figure 3.**
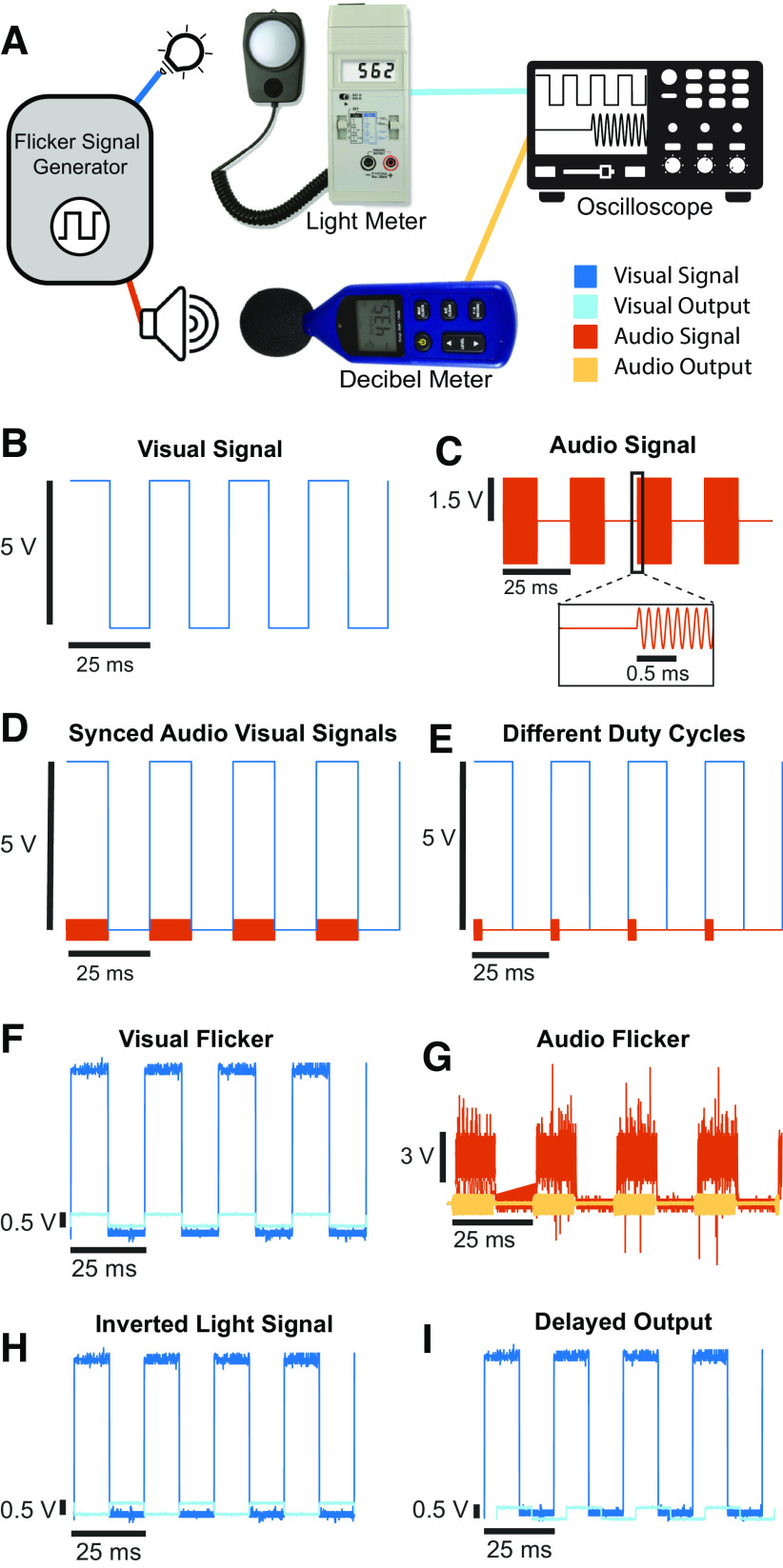
Measuring BrainWAVE stimulator output signals. ***A***, Visual and auditory stimuli generated by signal generators are measured with a light meter and decibel meter, respectively. Connecting these devices to an oscilloscope allows visualization and quantification of frequency and duty cycle. ***B***, Ideal 40-Hz visual signal with a 50% duty cycle. ***C***, Ideal 40-Hz audio signal (8-kHz tone) with a 50% duty cycle. Inset, a zoomed-in view of the signal at the stimulus onset showing an 8-kHz sinusoid. ***D***, Ideal 40-Hz visual (blue) and audio (orange) signals in phase, both with a 50% duty cycle. ***E***, Example of an ideal 40-Hz visual signal (blue) with a 50% duty cycle in phase with a 40-Hz audio signal (orange) with a 4% duty cycle. ***F***, Example of a real 40-Hz visual signal (dark blue) and its light output (light blue) recorded with an oscilloscope. ***G***, Example of a real 40-Hz audio signal (dark orange) and its audio output (light orange) recorded with an oscilloscope. ***H***, Same as ***F***, but of a BrainWAVE stimulator with an inverted MOSFET, which turns the lights off with a high-voltage signal and turns LEDs on with a low-voltage signal. ***I***, Same as ***F***, but with a BrainWAVE stimulator that uses a light source with a slower onset time. A delay is observed in the onset timing between the visual signal (dark blue) and measured output (light blue). See Extended Data Table 3-1 for recommended light and sound levels.

After constructing BrainWAVE stimulators, testing was performed to determine whether the devices generate appropriate stimulus intensity, timing, and other signal properties. Light illuminance and audio volume were measured with a light meter and decibel meter, respectively, with the distance between the sensor and meter approximating the distance from the sensory to the subjects’ eyes and ears (Extended Data Table 3-1). For mouse studies, light intensity was set at ∼150 lux and sound intensity at 60–65 dB (Garza et al., 2020; Martorell et al., 2019). For human studies, we adjusted stimulus intensity for each subject based on tolerance, with the levels ranging from 0 to 1400 lux for brightness and 0–80 dBA for sound (He et al., 2021). We measured the frequency and duty cycle of the audio and visual stimuli in real-time using an oscilloscope connected to the analog output ports of the light and decibel meters (Fig. 3*A*). Alternatively, the timing of the light and sound stimulus may be measured with a photodiode and a microphone connected to an oscilloscope, or the stimulus may be recorded on a laptop and analyzed on a computer. Audio and visual signals were measured simultaneously to compare their duty cycle, frequency, and phase timing.

10.1523/ENEURO.0257-22.2022.t3-1 Extended Data Table 3-1 BrainWAVE light and sound levels. These volume for the illuminance of LEDs and the volume of a speaker producing flicker stimulation from have been successfully used in prior studies to comfortably produce γ modulation in humans and mice. Download Table 3-1, DOCX file.

To modulate neural activity, we generated sensory signals at specific frequencies depending on the experimental design. Visual γ flicker (40 Hz) was produced using a 5.17-V, 40-Hz square wave with a 50% duty cycle (Fig. 3*B*). The voltage must be greater than 4 V to operate the MOSFET. Auditory γ flicker was produced with a pure sinusoid tone signal that was modulated by a 40 Hz square wave with a 50% duty cycle for audiovisual stimulation, and a 4% duty cycle for audio-only stimulation (Fig. 3*C*). The pure tone used was adjusted to fall within the center of the hearing range of the species tested: 10 kHz for mice and 7 or 8 kHz for humans (Heffner and Heffner, 2007). We used a 4% duty cycle for audio-only stimulation to more closely match the timing of clicks in studies on auditory steady-state responses evoked with 40-Hz click trains (Galambos et al., 1981; Stapells et al., 1984; Osipova et al., 2006; Ma et al., 2013; Thuné et al., 2016). Other frequencies of sensory were generated in a similar manner typically with a 50% duty cycle. Randomized stimulation was used to compare periodic to aperiodic flicker stimulation and had varying duty cycles (from 33% to 99%). Audio and visual signals were typically synchronized with similar duty cycles, but offset signals or different duty cycles may be desired in some cases (Fig. 3*D–G*,*I*).

### Presenting BrainWAVE stimuli to humans and mice

**Figure 4.**
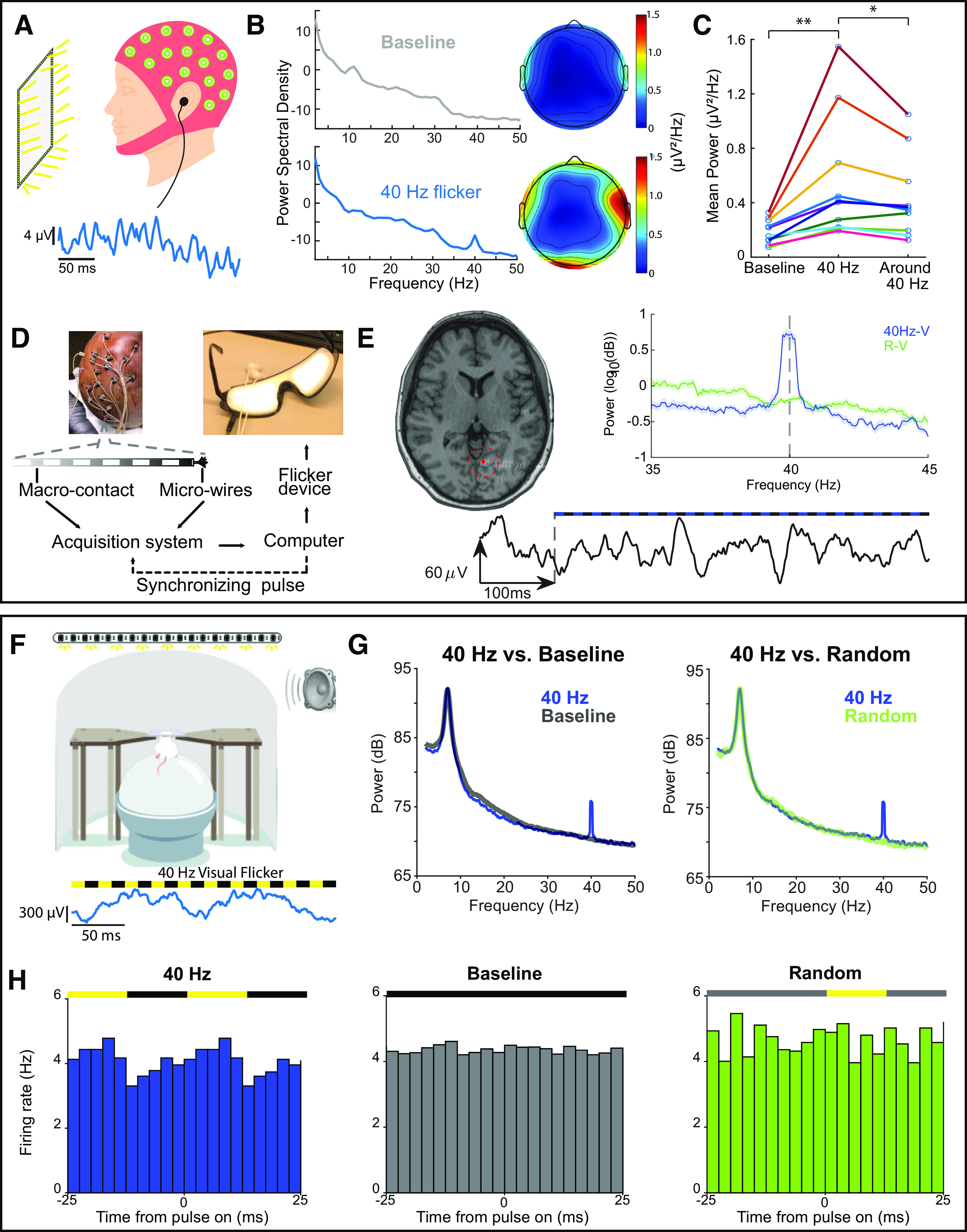
Sensory flicker entrains brain regions in humans and rodents. ***A***, Top, Schematic of a typical electroencephalogram (EEG) recording setup. Participants with EEG caps sat in front of a computer monitor (∼2 feet away) framed by LEDs and received auditory stimulation through earbuds. Bottom, Example EEG trace recorded from the center electrode (Cz) during flicker stimulation. ***B***, Left, Power spectral density averaged across all channels in an example EEG recording during baseline (top) and 40-Hz audiovisual flicker stimulation (bottom). Right, Heatmaps of mean power at 40 Hz averaged across subjects before (top) and after (bottom) 40-Hz stimulation. ***C***, Mean power at 40 Hz during baseline (“Baseline”) and during 40-Hz audiovisual flicker stimulation (“40 Hz”), and mean power at neighboring frequencies (31–39 and 41–49 Hz) during 40-Hz audiovisual stimulation (“Around 40 Hz”). Mean power at 40 Hz is significantly higher during 40-Hz stimulation than power at 40 Hz during baseline (*n* = 10 participants, *p* = 0.002, two-sided Wilcoxon signed-rank test) or power at neighboring frequencies (*n* = 10 participants, *p* = 0.014, two-sided Wilcoxon signed-rank test). Each colored line represents a single participant. * *p* ≤ 0.05, ** *p* ≤ 0.01. ***D***, Example setup of a human intracranial recording during flicker stimulation. Local field potential recordings were obtained from treatment-resistant epileptic patients implanted with intracranial electrodes for seizure monitoring. A computer controlled the delivery of sensory stimuli via a custom-made BrainWAVE stimulator circuit, which sent its output to a set of LED-lined goggles and earbuds. ***E***, Example of modulation to visual flicker recorded in lingual gyrus. Left, Axial slice of preimplant T1 MRI overlaid with postimplant computerized tomography (CT) scan, showing the location of the recording depth electrode. Highlighted in red is the electrode for data on right. Example recording trace before and during stimulation where the start of stimulation is indicated with a dashed line (below). Right, Power spectral density averaged across 15 trials of either 40-Hz visual (blue) or random visual stimulation (green). Shaded areas represent SEM (*n* = 15 trials in 1 participant, *p* = 0.00005, paired *t* test). ***F***, Top, Schematic of *in vivo* electrophysiology in head-fixed mice. Mice running on a spherical treadmill received sensory flicker stimulation through a strip of LEDs placed above the mouse and a speaker to the right. Bottom, Example trace of local field potentials in mouse auditory cortex during 40-Hz audiovisual flicker. ***G***, Power spectral density comparison between 40-Hz flicker stimulation (blue) and baseline (gray), and between 40-Hz flicker and random (green) condition. ***H***, Firing rate modulation during 40-Hz audiovisual stimulation (left), baseline (middle), and random (right) conditions in mouse hippocampus. Colors above indicate if light was on (yellow), off (black), or varied trial-to-trial (gray).

All human studies were approved and monitored by the Institutional Review Board. For EEG studies, male and female human subjects (ages 18–24) received the audio stimulus of a 7- or 8-kHz tone via headphones or earbuds. The visual stimulation was produced using an LED frame surrounding a PC monitor (Fig. 4*A*) or via glasses lined with LEDs. Both the tone and LEDs were synchronized. Before experiments, subjects were presented with multiple levels of light and audio intensities to identify the optimal range the subject tolerated. Subjects were allowed to ask the researcher to change the stimulus intensity to a level they were comfortable with at any point during the study. After finding a comfortable stimulation level, we noted the new stimulation level and then checked the subject’s EEG to see whether they still met our modulation criteria. Any subjects that did not meet modulation criteria were excluded from the study. In neural recording studies, a “relative occluded” stimuli recording condition was performed to test for electrical noise from the BrainWAVE stimulator. In human studies, during the relative occluded condition, the participant wore an opaque eye mask and earplugs that prevented exposure to the sensory stimuli. Neural activity of participants wearing the eye mask and ear plugs during this occluded condition session was not modulated when the sensory stimulation was turned on.

The MRI environment poses additional challenges because standard circuit components and stimulus devices are incompatible with the high magnetic field of the scanner. Thus, our lab developed novel methods for conducting flicker in the MRI scanner. First, we designed stimulus presentation methods that were MR safe. During MRI scans, we delivered audio flicker stimulation using in-ear MR safe, headphones (brand: MRIAudio) to allow the participant to hear audio generated from a PC or BrainWAVE stimulator over scanner noises. These headphones were designed to fit within the MRI head coil during scans and have a noise reduction rating (NRR) of 29 dB. For visual flicker, we created an MR safe LED frame that fits around a projection screen. This setup allowed participants to view pictures or videos or perform visual-based tasks while receiving visual flicker stimulation in their periphery. The translucent projection screen with the LED frame was constructed on an MR safe stand placed about two feet from the patient table of the MRI machine. We did not find MRI artifacts with this screen or the headphones.

Second, we designed a system such that the control circuit was far enough from the scanner (placed in the MRI control room) while the lights and sounds were near the MRI scanner so that the stimulation signals from the control circuit were not affected by the scanner’s radio frequency pulses and so that the circuit’s signals did not create noise in the MRI images. A shielded cable was used to connect the flicker stimulation device in the control room to the LED frame. We controlled and adjusted the volume and brightness of flicker from the control room using a computer or BrainWAVE stimulator. A projector in the control room projected images through the control room window onto the back of the projection screen. A front-facing mirror placed above the head coil mirrored the screen and flicker frame to the subject lying down in the scanner. We also included simple attention tasks to determine whether the subject was alert over the course of an experiment.

**Figure 5.**
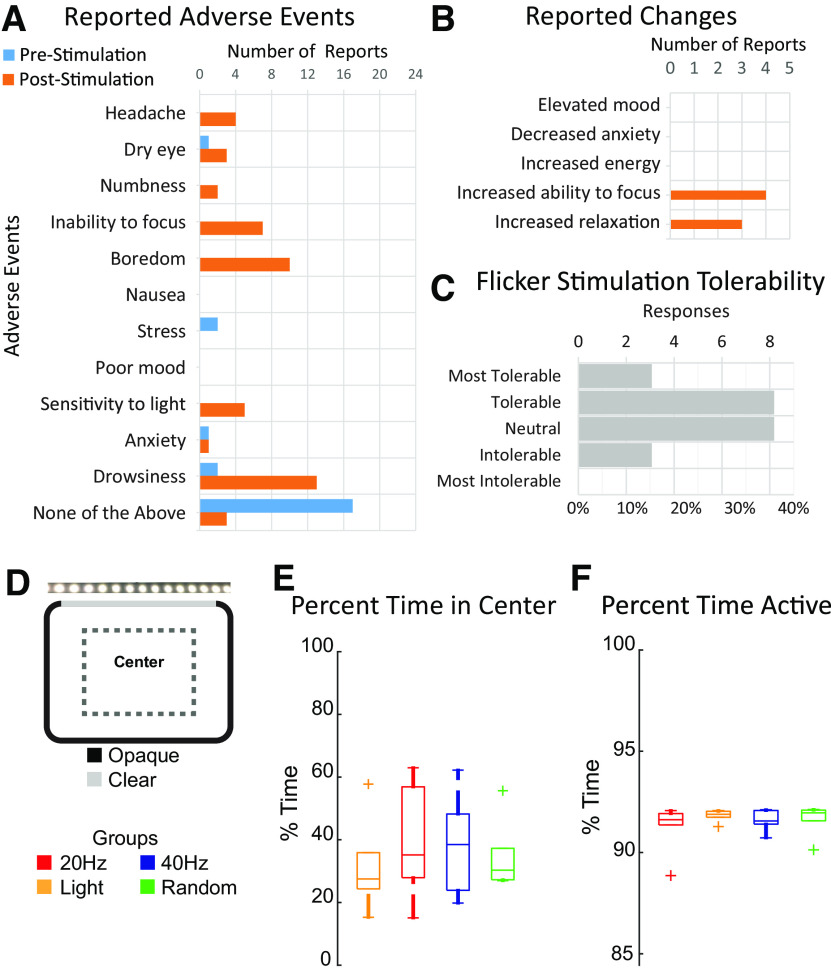
Minimal adverse effects from flicker exposure. ***A***, Number of reported mild adverse events by participants (*n* = 22) before or after 1 h of audiovisual flicker stimulation during an EEG or MRI session. ***B***, Number of reported changes in mood, anxiety, energy, focus, or relaxation (*n* = 22). ***C***, The distribution of responses from participants (*n* = 22) rating the tolerability of flicker stimulation after 1 h of flicker during an EEG or MRI session. ***D***, Schematic of flicker stimulation for freely moving mice in an enclosure. Overhead view of mouse enclosure with a portion of the arena indicating the center zone which was used for analysis. ***E***, Percentage of time spent in the center zone during a 1-h session of constant light (yellow), 20 Hz (red), 40 Hz (blue), and random (green) conditions (*F*_(3,15)_ = 0.8754, *p* = 0.4757, RM-ANOVA, *n* = 5 mice). Error bars indicate the mean ± SEM. ***F***, Total percent of time mice were active during the 1-h session of flicker (*F*_(3,15)_ = 0.9306, *p* = 0.4502, RM-ANOVA). Error bars indicate the mean ± SEM. See Movie 1 for mouse exposure to sensory flicker.

All animal work was approved by the Institutional Animal Care and Use Committee at the Georgia Institute of Technology. For mouse studies, wild-type male two- to three-month-old mice were brought into the animal holding areas and left undisturbed for at least 30 min. Mice were then moved to a dark experimental room and individually placed in an empty enclosure with three opaque black sides (Fig. 5*D*). One transparent side of the cage faced LED strips. An audio stimulus was presented through a speaker and synchronized to the onset and offset of the LEDs. The stimulus was presented for 1 h or for 1 h/d for multiple days. We used multiple different stimulation conditions as controls, namely, random, 20 Hz, and constant light. The random group received sensory stimulation with randomized light-off intervals (duration ranging from 0 to 25 ms) while the total duration of the light-on phase was kept consistent with the 40-Hz group (12.5 ms). The 20-Hz group was exposed to 20-Hz light flicker with a 50% duty cycle. The random condition was used to assess the effects of periodic versus aperiodic stimuli and the 20-Hz condition was used to assess stimulus frequency-dependent effects. The constant light group was exposed to constant light for the entire duration of the session and therefore used to disambiguate the effects of constant versus flickering stimuli.

Movie 1. Mouse BrainWAVE exposure. A mouse is exposed to 40-Hz audio visual flicker. 10.1523/ENEURO.0257-22.2022.video.1

### Blinding

A key consideration in flicker experiments is how to blind experimenters when different experimental stimuli are readily perceived during experiments. To address this, we developed a blinding system such that the experimenter could test and start flicker without being exposed to flicker. In this system, animals were monitored remotely using an infrared (IR) light source and a video camera with an IR-pass filter. The IR filter blocked visible light, thus preventing the experimenter from determining the light flicker frequency, while the IR light source provided illumination to monitor animal behavior. Animals were assigned to stimulation groups by a nonblinded third party and each animal identifier and the corresponding flicker group assignment were listed in a file that was read by the flicker device. To start flicker, a blinded experimenter entered the animal’s unique identifier into the flicker device, which then loaded the correct stimulation condition corresponding to the animal’s identifier. Before starting the flicker stimulation sequence or control condition sequence, the device first played a test sequence of light and sound so that the experimenter could confirm that the LEDs and speaker were working properly. After the test sequence, there was a pause in stimulation during which the experimenter exited the room to the remote monitoring computer. Flicker stimulation or the control stimulation condition then commenced while the experimenter monitored the animal via the IR video in another room.

### Electrophysiology recording, preprocessing, and analyses of neural data

We performed neural recording and analyses as described previously (Iaccarino et al., 2016; Martorell et al., 2019; He et al., 2021). In brief, for *in vivo* electrophysiology in mice, we made small craniotomies on the skull of the animal under isoflurane anesthesia using the coordinates for brain regions of interest. We recorded local field potential (LFP) and spiking activity using a 32-channel NeuroNexus probe with data acquired at a sampling rate of 20 kHz using an Intan RHD2000 Evaluation System with a ground pellet as reference (Fig. 4). Additional recordings were performed with the electrode in saline above the craniotomy while animals were exposed to flicker to detect possible electrical artifacts. Recorded neural data were bandpass filtered from 300 to 6000 Hz for spikes to be clustered into single units using an automatic spike sorting algorithm (MountainSort). For LFP analyses, raw data were first downsampled to 2 kHz and bandpass filtered from 1 to 300 Hz. For power spectral density analyses, we used multitaper methods from the MATLAB Chronux toolbox and compared traces between 40 Hz and random frequency stimulation.

For the human scalp EEG recordings, we used a 32-channel BioSemi ActiveTwo system with data acquired at a sampling rate of 2048 Hz (Fig. 4). Signals were bandpass filtered from 1 to 100 Hz using Hamming windowed FIR filter (EEGLAB) for power spectral density analyses. We defined modulation of individual channels as elevated power at 40 Hz, at least three standard deviations above the mean power in neighboring frequencies of 31–39 and 41–49 Hz. We defined modulation of an individual human subject as having at least three modulated channels with at least one modulated channel in either hemisphere over the course of a recording.

Human intracranial recordings were performed in treatment-resistant epileptic patients who underwent presurgical intracranial seizure monitoring to determine their seizure onset zones (Fig. 4*D*). To record LFPs, these patients were usually implanted with a dozen (number and location determined based on clinical needs) depth electrodes, each containing up to 18 macro-contacts along their length. Some of these electrodes contained microwires that protruded at their tips and allowed recordings from single neurons. Typically, these patients were monitored for several days to weeks, providing a unique opportunity to perform voluntary studies on intracranial human brain activity in between clinical care. We conducted all experiments in the patient’s room. LFP recordings were acquired using the clinical system used by the hospital (XLTEK EMU 128FS; Natus Medical) at a rate ranging from 1024 to 2048 or higher Hertz using subdermal contacts from an electrode array placed at the vertex (subgaleal) as ground and reference. Signals from microwires were recorded using the Blackrock NeuroPort system (Blackrock Microsystems, UTSW), at a rate of 30,000 samples/s, using a dedicated microwire as reference. Data were re-referenced using Laplacian reference and bandpass filtered between 2 and 300 Hz, with a baseline correction over the duration of 12-s records segments. PSD was calculated over 2–100 Hz, using the Chronux toolbox (Mitra and Bokil, 2009; http://chronux.org/), with a time-bandwidth product of 3, and number of tapers of 5.

### Behavioral assessment

Human study participants were given a survey immediately before and after a 1-h audio and visual flicker stimulation to assess any acute symptoms such as headache, dizziness, and negative affect (Fig. 5*A–C*).

To monitor the effects of sensory flicker on animal behavior during stimulation, we recorded mouse behavior using an IR-sensitive camera (Fig. 5*D*). The infrared lighting was required to avoid poor video tracking because of interference from the flickering visible light and to avoid experimenter bias. Behavior was analyzed via automated animal tracking (Ethovision XT v 14.0). We divided the arena into center and outer regions and quantified activity levels in both parts (Fig. 5*E*). Animal activity was quantified by classifying periods as activity or inactivity/freezing. Inactivity was defined as having activity in 0.01% of the total arena for longer than 0.5 s (Fig. 5*F*,*G*). We performed a one-way ANOVA to assess group differences.

## Results

To deliver flicker stimulation to humans and mice, we developed custom BrainWAVE devices (Fig. 1). Standard computer monitors and projectors do not have fast enough refresh rates to achieve 40-Hz flicker, thus custom-built LED BrainWAVE stimulators are required. The BrainWAVE stimulator interfaced with a variety of light sources and speakers as output components to accommodate different experimental needs (Fig. 1*A–C*). The output components included LED lights or a PC monitor with a high refresh rate (e.g., 165 Hz or greater) for visual signals, and speakers, headphones, or earbuds for auditory signals. To illustrate the flexibility and customizability of our device, we provide several output components we have used successfully. For mouse studies, we used LED light strips and speakers (Fig. 1*A*) to expose animals to flicker stimulation while the mice were able to freely move within their cages. For human studies, we used LED goggles or LED monitor frames, and headphones or speakers (Fig. 1*B*,*C*). Flickering LED light strips attached to the edges of a monitor were used in studies where participants perform memory and attention/reaction tasks on a PC while receiving flicker (Fig. 1*C*). We also designed LED frames and panels that use extra bright LEDs to send light from a distance to the visual field of a participant laying down within an MRI scanner (Fig. 1*C*). These different outputs interfaced with a common, compact, and portable circuit (Fig. 1*D–F*). A detailed parts list and instructions for BrainWAVE stimulator circuit assembly are provided (see Extended Data Table 1-1). We developed a user-friendly application to run a variety of experiments involving visual and/or auditory stimulation (Fig. 2). These examples demonstrate the feasibility and customizability of the BrainWAVE stimulator in a variety of settings.

### Modulation in humans

To establish the effects of flicker stimulation in humans, we characterized neural activity with and without 40-Hz audio/visual stimulation during both scalp EEG recordings and intracranial recordings in human participants (Fig. 4*A*,*D*). Using scalp EEG, we found a significant increase in EEG power at 40 Hz during stimulation relative to no-stimulation baseline periods within the same subjects (*n* = 10 participants, *p* = 0.002, two-sided Wilcoxon signed-rank test; Fig. 4*B*,*C*). This increase in EEG power during 40-Hz flicker was observed across multiple channels, with channels located over visual and auditory regions having higher modulation (Fig. 4*B*). Importantly, elevated EEG power was specific to the frequency of stimulation; we found a significant difference in the power at 40 Hz compared with the mean power at neighboring frequencies (e.g., 40 vs 31–39 and 41–49 Hz; Fig. 4*C*; *n* = 10 participants, *p* = 0.014 two-sided Wilcoxon signed-rank test). Furthermore, elevated EEG power was because of sensory stimulation itself and not electrical artifacts since there was no significant increase in 40-Hz power during the occluded condition (*p* = 0.56, paired *t* test). While there was variability across subjects, modulation of at least three channels with at least one channel in each hemisphere was achieved within 10 s after the onset of stimuli and lasted for the duration of flicker exposure (data not shown).

To determine the effects of flicker stimulation in humans with better spatial resolution, we recorded neural activity intracranially in treatment-resistant epileptic patients undergoing presurgical intracranial seizure monitoring with stereotactic EEG (Fig. 4*D*,*E*) and applied offline highly localizing Laplacian re-referencing to the LFP. We found that sensory flicker increased LFP oscillations, indicative of population dendritic activity, at the frequency of the flickering stimulus in auditory and visual cortices. As an example, in the early visual processing lingual gyrus, 40-Hz visual stimulation induced an increase in power at the frequency of stimulation, which is not present in the random visual flicker condition, our control condition (*n* = 15 trials in one subject, *p* = 0.0005, paired *t* test, power at 40-Hz medians and quartiles: 4.05 dB, 3.47–5.07 dB during 40-Hz flicker; 0.68 dB, 0.46–0.87 dB during random flicker; Cohen’s *d* = 2.05; Fig. 4*E*). By applying Laplacian re-referencing to the LFP, where the average of adjacent contacts’ signals is subtracted from the signal of the channel of interest, we determined this modulation was local and was not because of distant volume conduction. Together, these findings demonstrate that the sensory flicker-induced modulation is reliable, frequency-specific, and efficiently induced.

### Modulation in mice

To assess the effects of flicker in sensory and memory circuits of mice with high temporal and spatial resolution, we recorded local field potentials and single neurons during flicker exposure. Using *in vivo* electrophysiology in awake, head-fixed mice, we quantified sensory flicker-induced changes in neural activity in mouse hippocampus (Fig. 4*F–H*). As previously reported by Martorell et al. (2019) and Iaccarino et al. (2016), we found that LFP power was significantly elevated specifically at 40 Hz during 40-Hz stimulation, but not during no-stimulation baseline or random control conditions (*n* = 8 trials in 1 animal, *p* = 0.010 40 Hz vs baseline, *p* = 0.004 40 Hz vs random, paired *t* test, power at 40-Hz medians and quartiles: 75.18 dB, 71.86–76.76 dB during 40-Hz flicker; 70.30 dB, 68.39–72.31 dB during baseline; 70.39 dB, 68.05–71.11 dB during random; Fig. 4*G*). Furthermore, exposing mice to 40-Hz auditory flicker led to increased modulation of single-neuron spiking, meaning neurons were more likely to fire at a particular phase of the stimulus, in hippocampus (Fig. 4*H*). These effects were not observed in no-stimulation baseline or random control conditions. Similar results were observed in auditory cortex and prefrontal cortex. These deeper regions are more difficult to target with other noninvasive stimuli, such as transcranial magnetic stimulation. These results demonstrate effective, frequency-specific, and noninvasive modulation of neural activity in multiple brain regions simultaneously using simple and customizable BrainWAVE stimulator circuits.

### Stimulation side effects and behavioral controls

One important consideration is whether flicker stimulation is aversive or has unintended effects on behavior. Accordingly, we asked our study participants to report any acute symptoms such as headache, dizziness, negative affect, and more in a survey immediately before and after a 1-h audio and visual flicker stimulation session. Out of eight participants, one reported that the light or sound was intolerable. After stimulation, some participants reported mild negative or positive effects including sleepiness/drowsiness (6), boredom (5), headache (2), increased (1) and decreased (2) ability to focus, and increased relaxation (1) (Fig. 5*A–C*). Some of these effects, such as drowsiness and boredom may be attributed to the experiment procedure which asked participants to remain still for over an hour, rather than being an effect caused by the stimulation itself.

In addition to the acute effects of flicker, we assessed the potential adverse effects of chronic flicker exposure (He et al., 2021). In this study, older participants with prodromal Alzheimer’s disease were exposed to four to eight weeks of flicker stimulation. Overall, longer-term flicker exposure was well tolerated by most subjects. Out of 10 participants, three reported mild adverse events which may be attributed to flicker exposure, including dizziness, tinnitus, headache, and worsened hearing loss. These mild adverse events were relatively rare, reported 5 times over the course of four to eight weeks of daily 1-h flicker exposure.

One consideration is that different types of flicker stimulation indirectly affect neural or immune responses because that particular type of stimulus makes animals move more or less. Our results show that animals respond similarly in terms of the amount of activity and exploration of the environment with flicker at different frequencies and constant light. These results show that neural and immune responses to sensory flicker cannot solely be attributed to changes in activity levels during stimulation, although there may be more subtle behavioral differences that could not be quantified with our assays. To determine whether the effects of sensory stimulation were confounded by changes in mouse behavior, we recorded the activity of mice during a 1-h session of visual stimulation (Garza et al., 2020; Fig. 5*D*,*E*). To do this, we took advantage of the fact that mice were allowed to move freely during stimulation. We quantified the amount of time spent in the center arena of the cage during each stimulation condition as a common measure of anxiety-like behavior in mice. We found no significant differences in both the time spent in the center of the arena and total activity across stimulation conditions [percent time in center: *F*_(3,15)_ = 0.8754, *p* = 0.4757, repeated measures (RM)-ANOVA, *n* = 5 mice; percent time active: *F*_(3,15)_ = 0.9306, *p* = 0.4502, RM-ANOVA; Fig. 5*F*,*G*]. These results show that neural and immune responses to sensory flicker cannot solely be attributed to changes in behavior during stimulation, although there may be more subtle behavioral differences not detected with our assays.

## Discussion

Here, we introduce a newly optimized BrainWAVE stimulator and user-friendly, open-source methods for assembling, testing, and implementing noninvasive sensory flicker across species and experimental designs. Building on our first example use of flicker in modifying disease pathology, here we developed several new methods to aid future research involving flicker. First, we integrated the audio and visual stimulation systems to produce synchronized multimodal stimulation. Second, we created a user-friendly, intuitive GUI to easily control and adjust flicker during experiments and without requiring programming. Third, we designed an MR-compatible flicker device to remotely control flicker during MRI. Fourth, we established a new protocol for designing and administering a blinded flicker study with appropriate control conditions. Finally, we validated the safety of flicker by assessing potential adverse effects in healthy human subjects. Our flicker stimulation produced robust, rapid, and frequency-specific modulation of neural activity in both mice and humans with minimal side effects. We showed that side effects of stimulation were rare and mild. These results were not because of differences in overall activity levels or anxiety-like behavior during stimulation. Thus, this new and improved cross-species simulation tool provides a unique means to study and treat neural activity deficits noninvasively in a wide spectrum of brain regions and diseases.

This optimized BrainWAVE stimulator described in the present work has several advantages to reach a wider scientific and medical community. We substantially improved our design to make our tools user-friendly, accessible, and therefore more impactful in research and medicine. With a simple and flexible design, sensory flicker is easy to integrate into a variety of experimental setups. These methodological improvements build on our previously demonstrated effects of sensory flicker. We previously found that audio and audiovisual sensory flicker noninvasively produces frequency-specific modulation in hippocampus and prefrontal cortex of mice (Martorell et al., 2019). The ability to target deep brain regions noninvasively is important because these regions are involved in memory and disease. Indeed, our prior study shows γ (40 Hz) flicker rescues memory deficits in a mouse model of Alzheimer’s disease (Martorell et al., 2019).

Our ability to flexibly alter the degree of synchronization between multiple sensory stimuli brings additional advantages to studying multimodal associative learning. For most of our previous studies, we have programmed our stimulator to generate auditory and visual signals simultaneously. However, for some experimental questions it is important to consider that the transmission times of auditory and visual information through their respective pathways differ (King and Palmer, 1985) and the magnitude of these differences can vary across species and individuals. Future experiments may examine how changing the onset and phase of these stimuli affect neural activity. Flexibly adjusting the delay between auditory and visual stimulation so that the sensory information reaches their respective cortices at the exact same time may indeed lead to more effective modulation in some brain structures. Furthermore, the BrainWAVE stimulator is capable of adjusting stimuli onset with millisecond precision to suit the researcher’s experimental design or to personalize stimuli to suit an individual or specie’s specific transmission speed. Prior work has shown that phase locking of visual and auditory stimuli enhances and predicts future long-term memory formation (Clouter et al., 2017; D. Wang et al., 2018). While we have not investigated this directly, enhancing phase locking may be part of the mechanism by which flicker improves memory in mice (Martorell et al., 2019).

γ Flicker drastically reduces levels of amyloid β levels, a peptide thought to initiate neurotoxic events in Alzheimer’s disease, in sensory and memory circuits (Iaccarino et al., 2016; Martorell et al., 2019). Noninvasive sensory stimulation is currently being tested in several clinical trials in patients with neurodegenerative diseases (Cimenser et al., 2021; He et al., 2021; Murty et al., 2021). Human participants have successfully used flicker at home with minimal side effects, proving long-term studies are feasible and convenient (Cimenser et al., 2021; He et al., 2021). Future versions of BrainWAVE stimulators may be integrated into existing wearable technology like smartwatches and virtual reality headsets.

When designing a human or animal flicker study, there are a few limitations and considerations to keep in mind. One of the recurring challenges is selecting a “control” condition or other stimuli for comparison. Possible control conditions include no sensory stimulation, alternate frequencies (such as 20 or 80 Hz), constant (nonflickering) stimuli, and random (nonperiodic) frequencies. Each type of stimulus condition controls for different aspects of the stimuli, such as periodicity, frequency, and total duration of stimulus exposure. Given these considerations, an ideal experimental design has multiple control groups with different types of stimulation parameters. When deciding on one or more control groups, group size and feasibility of an experiment may be limiting factors. As an additional limiting factor in human flicker use, potential negative side effects must be considered and minimized. In this work and in a prior study, we excluded participants with a history of light-induced seizures or migraines in case the stimulus exacerbates these conditions (He et al., 2021). Although mild and rare, we noted some adverse side effects of acute flicker stimulation, including boredom, sensitivity to light, and headache which were similar to mild adverse events in our prior study on chronic stimulation (He et al., 2021). To help mitigate potential adverse effects, participants may be given the option to adjust the stimulus intensity to more comfortable levels. However, researchers should keep in mind that lowering the intensity may decrease the degree of modulation, and if lowered below a certain point, neural activity modulation may not be observed. Indeed, studies should establish the degree to which participants’ neural activity modulates to the flicker stimulus before studying subsequent effects and establish baseline levels of acute modulation. In our studies, we first establish light and sound intensity levels at which the subject is comfortable as well as levels that show adequate modulation to the stimulus. We recommend testing multiple ranges of light and sound intensity before an experiment to include participants with robust modulation at tolerated intensities and exclude those reporting discomfort with the stimuli.

While most of our prior work has thus far focused on the effects of 40-Hz flicker in neurodegenerative disease, the effects of stimulation in other diseases and in the healthy brain are currently under investigation (Iaccarino et al., 2016; Martorell et al., 2019; Garza et al., 2020; He et al., 2021; Zheng et al., 2020). One study has reported that 30- to 50-Hz flicker protects hippocampal neurons in an animal model of ischemia (Zheng et al., 2020). Another study showed that 60-Hz light flicker affects microglia remodeling of perineuronal nets, which play a key role in critical period plasticity, in healthy mice (Venturino et al., 2021). Our prior study shows that light flicker has frequency-specific effects on the expression of cytokines, an extracellular immune signaling protein, as well as intracellular immune signaling in healthy adult animals (Garza et al., 2020). These studies reveal that flicker could be used as a novel intervention in a variety of contexts. For example, this noninvasive means of driving brain rhythms is valuable for assessing immune effects of specific activity patterns without the confounding effects of invasive stimulation tools.

Inducing frequency-specific neural activity noninvasively using sensory flicker provides a novel approach to investigating the role of specific frequencies of neural activity in health and disease. Here, we provide an easy-to-follow guide to build and implement such devices in experimental and clinical settings at low cost and with user-friendly software. These tools will be useful to future studies using our devices that will produce novel insights into the mechanisms of brain rhythms and immune function in health and disease.

## Synthesis

Reviewing Editor: Arvind Kumar, KTH Royal Institute of Technology Decisions are customarily a result of the Reviewing Editor and the peer reviewers coming together and discussing their recommendations until a consensus is reached. When revisions are invited, a fact-based synthesis statement explaining their decision and outlining what is needed to prepare a revision will be listed below. The following reviewer(s) agreed to reveal their identity: Boateng Asamoah, Kimron Shapiro.

The two reviewers are happy with the revisions. They do not have any further concerns about the manuscript. So I am happy to inform you that the manuscript can be accepted in its current form.
