# Supplementary figures and images for: BrainWAVE: A Flexible Method for Noninvasive Stimulation of Brain Rhythms across Species (part 1 of 2)
Source: eNeuro. 2023 Feb 23;10(2):ENEURO.0257-22.2022. doi: 10.1523/ENEURO.0257-22.2022 (PMC9979148; doi:10.1523/ENEURO.0257-22.2022)

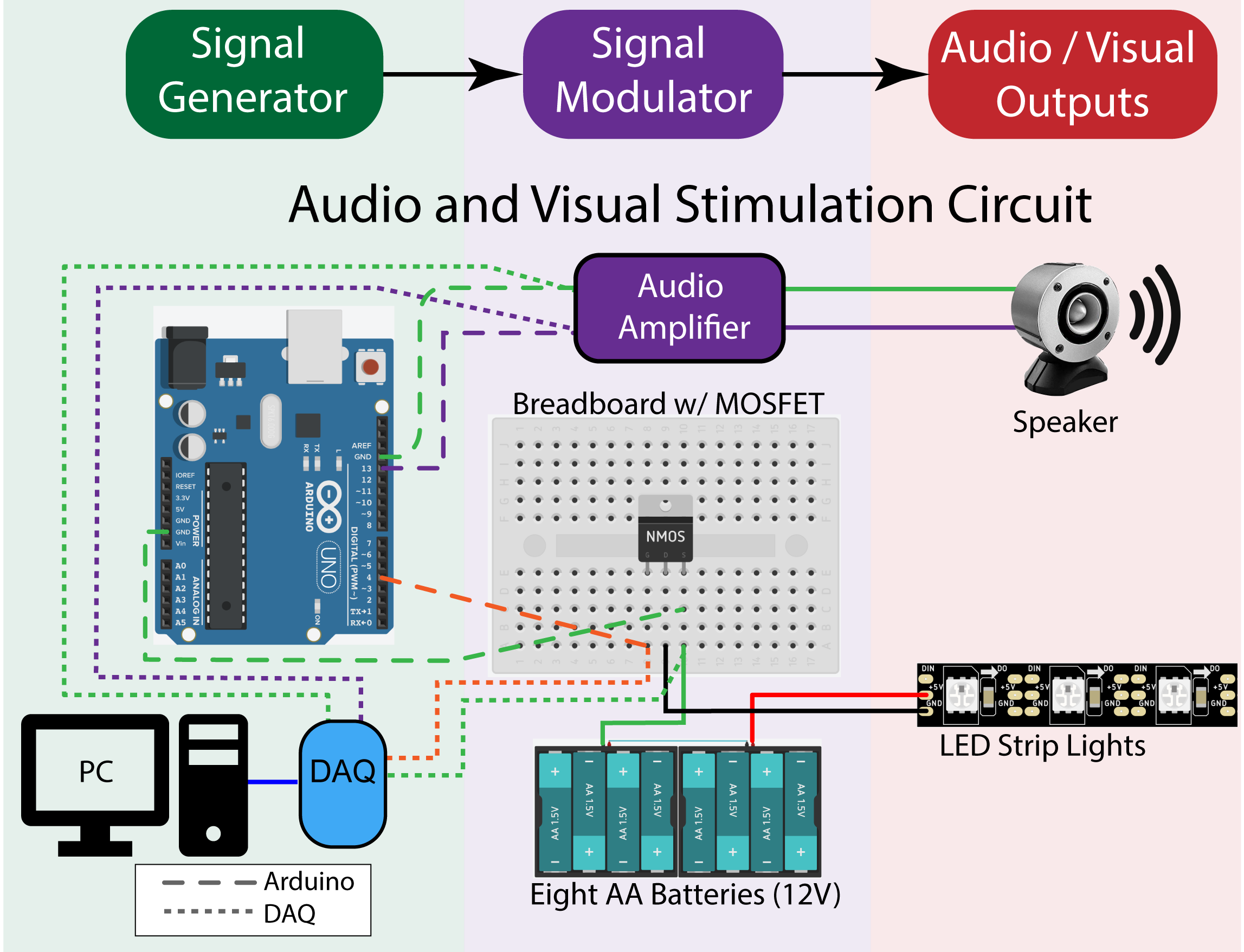

Supplement: Extended Data Figure 1-1 — Diagram of audio-visual BrainWAVE circuit. The audio and visual signal generators (green), an Arduino Uno, and a data acquisition device (DAQ) supply signals to the signal modulators (purple), an audio amplifier and a MOSFET, which then control the outputs (red) for this circuit, a speaker and LED strip lights. Eight 1.5-V AA batteries supply power to a strip of LEDs. Download Figure 1-1, TIF file. [file enu-eN-OTM-0257-22-s03.tif]

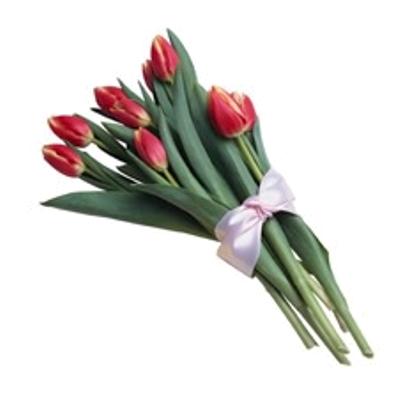

Supplement: Extended Data 1 — BrainWAVE stimulator code. These files contain code to generate and play flicker sensory stimulation with an Arduino Uno or NIDAQ BrainWAVE stimulator device. Download Extended Data 1, ZIP file. [file enu-eN-OTM-0257-22-s05.zip › Code_FliCkER/Code_FliCkER/GUI_FliCkER/functions_and_parameters/FlickerMemoryTask/imageset_1a/Set1_001a.jpg]

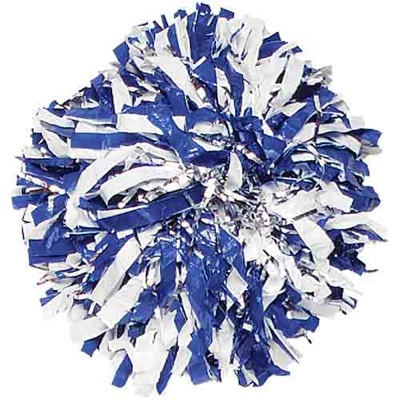

Supplement: Extended Data 1 — BrainWAVE stimulator code. These files contain code to generate and play flicker sensory stimulation with an Arduino Uno or NIDAQ BrainWAVE stimulator device. Download Extended Data 1, ZIP file. [file enu-eN-OTM-0257-22-s05.zip › Code_FliCkER/Code_FliCkER/GUI_FliCkER/functions_and_parameters/FlickerMemoryTask/imageset_1a/Set1_002a.jpg]

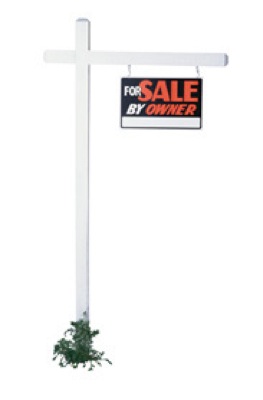

Supplement: Extended Data 1 — BrainWAVE stimulator code. These files contain code to generate and play flicker sensory stimulation with an Arduino Uno or NIDAQ BrainWAVE stimulator device. Download Extended Data 1, ZIP file. [file enu-eN-OTM-0257-22-s05.zip › Code_FliCkER/Code_FliCkER/GUI_FliCkER/functions_and_parameters/FlickerMemoryTask/imageset_1a/Set1_003a.jpg]

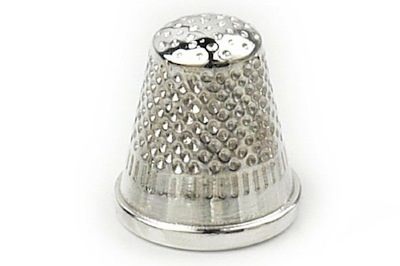

Supplement: Extended Data 1 — BrainWAVE stimulator code. These files contain code to generate and play flicker sensory stimulation with an Arduino Uno or NIDAQ BrainWAVE stimulator device. Download Extended Data 1, ZIP file. [file enu-eN-OTM-0257-22-s05.zip › Code_FliCkER/Code_FliCkER/GUI_FliCkER/functions_and_parameters/FlickerMemoryTask/imageset_1a/Set1_004a.jpg]

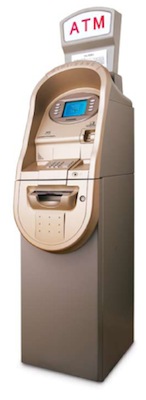

Supplement: Extended Data 1 — BrainWAVE stimulator code. These files contain code to generate and play flicker sensory stimulation with an Arduino Uno or NIDAQ BrainWAVE stimulator device. Download Extended Data 1, ZIP file. [file enu-eN-OTM-0257-22-s05.zip › Code_FliCkER/Code_FliCkER/GUI_FliCkER/functions_and_parameters/FlickerMemoryTask/imageset_1a/Set1_005a.jpg]

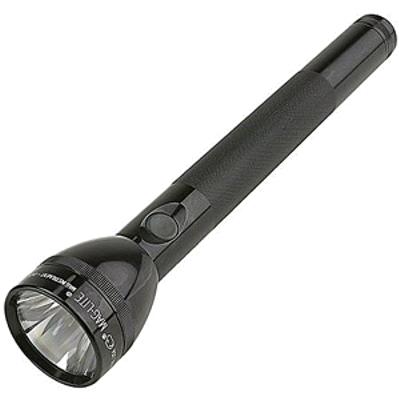

Supplement: Extended Data 1 — BrainWAVE stimulator code. These files contain code to generate and play flicker sensory stimulation with an Arduino Uno or NIDAQ BrainWAVE stimulator device. Download Extended Data 1, ZIP file. [file enu-eN-OTM-0257-22-s05.zip › Code_FliCkER/Code_FliCkER/GUI_FliCkER/functions_and_parameters/FlickerMemoryTask/imageset_1a/Set1_006a.jpg]

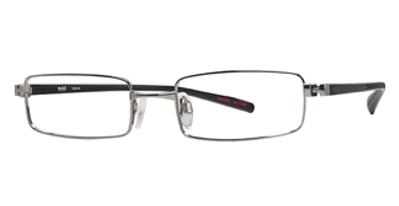

Supplement: Extended Data 1 — BrainWAVE stimulator code. These files contain code to generate and play flicker sensory stimulation with an Arduino Uno or NIDAQ BrainWAVE stimulator device. Download Extended Data 1, ZIP file. [file enu-eN-OTM-0257-22-s05.zip › Code_FliCkER/Code_FliCkER/GUI_FliCkER/functions_and_parameters/FlickerMemoryTask/imageset_1a/Set1_007a.jpg]

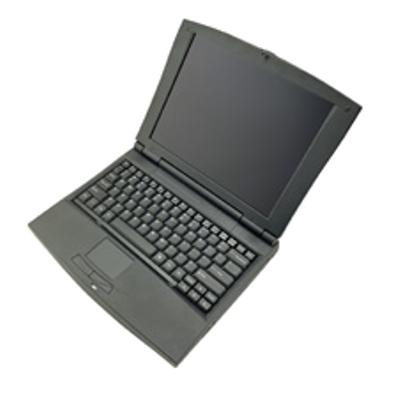

Supplement: Extended Data 1 — BrainWAVE stimulator code. These files contain code to generate and play flicker sensory stimulation with an Arduino Uno or NIDAQ BrainWAVE stimulator device. Download Extended Data 1, ZIP file. [file enu-eN-OTM-0257-22-s05.zip › Code_FliCkER/Code_FliCkER/GUI_FliCkER/functions_and_parameters/FlickerMemoryTask/imageset_1a/Set1_008a.jpg]

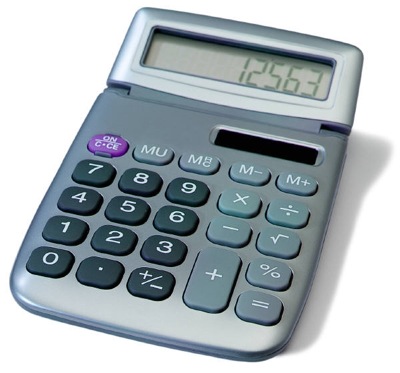

Supplement: Extended Data 1 — BrainWAVE stimulator code. These files contain code to generate and play flicker sensory stimulation with an Arduino Uno or NIDAQ BrainWAVE stimulator device. Download Extended Data 1, ZIP file. [file enu-eN-OTM-0257-22-s05.zip › Code_FliCkER/Code_FliCkER/GUI_FliCkER/functions_and_parameters/FlickerMemoryTask/imageset_1a/Set1_009a.jpg]

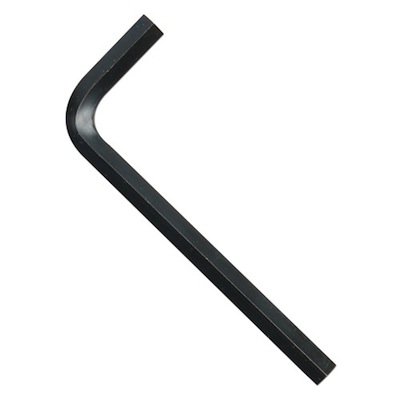

Supplement: Extended Data 1 — BrainWAVE stimulator code. These files contain code to generate and play flicker sensory stimulation with an Arduino Uno or NIDAQ BrainWAVE stimulator device. Download Extended Data 1, ZIP file. [file enu-eN-OTM-0257-22-s05.zip › Code_FliCkER/Code_FliCkER/GUI_FliCkER/functions_and_parameters/FlickerMemoryTask/imageset_1a/Set1_011a.jpg]

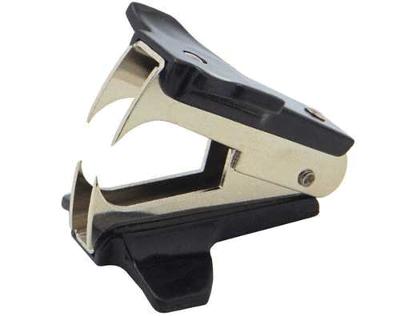

Supplement: Extended Data 1 — BrainWAVE stimulator code. These files contain code to generate and play flicker sensory stimulation with an Arduino Uno or NIDAQ BrainWAVE stimulator device. Download Extended Data 1, ZIP file. [file enu-eN-OTM-0257-22-s05.zip › Code_FliCkER/Code_FliCkER/GUI_FliCkER/functions_and_parameters/FlickerMemoryTask/imageset_1a/Set1_012a.jpg]

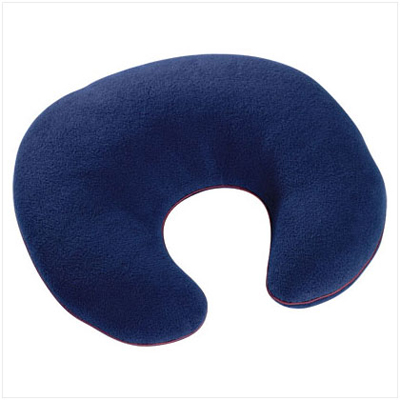

Supplement: Extended Data 1 — BrainWAVE stimulator code. These files contain code to generate and play flicker sensory stimulation with an Arduino Uno or NIDAQ BrainWAVE stimulator device. Download Extended Data 1, ZIP file. [file enu-eN-OTM-0257-22-s05.zip › Code_FliCkER/Code_FliCkER/GUI_FliCkER/functions_and_parameters/FlickerMemoryTask/imageset_1a/Set1_013a.jpg]

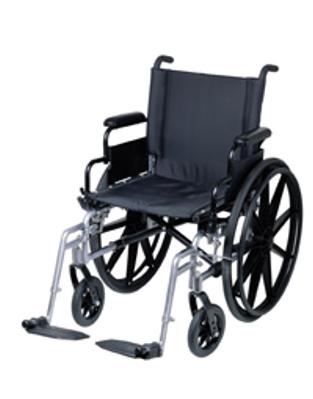

Supplement: Extended Data 1 — BrainWAVE stimulator code. These files contain code to generate and play flicker sensory stimulation with an Arduino Uno or NIDAQ BrainWAVE stimulator device. Download Extended Data 1, ZIP file. [file enu-eN-OTM-0257-22-s05.zip › Code_FliCkER/Code_FliCkER/GUI_FliCkER/functions_and_parameters/FlickerMemoryTask/imageset_1a/Set1_014a.jpg]

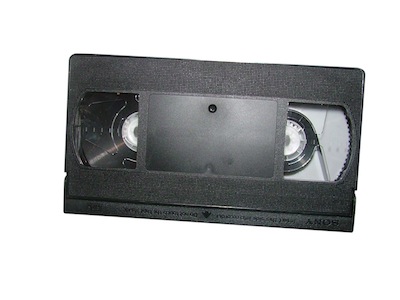

Supplement: Extended Data 1 — BrainWAVE stimulator code. These files contain code to generate and play flicker sensory stimulation with an Arduino Uno or NIDAQ BrainWAVE stimulator device. Download Extended Data 1, ZIP file. [file enu-eN-OTM-0257-22-s05.zip › Code_FliCkER/Code_FliCkER/GUI_FliCkER/functions_and_parameters/FlickerMemoryTask/imageset_1a/Set1_015a.jpg]

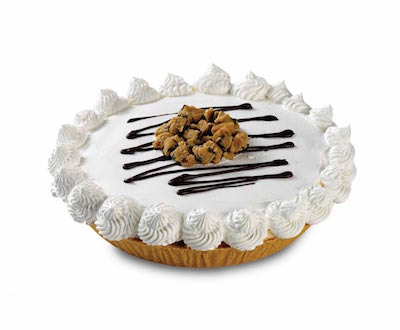

Supplement: Extended Data 1 — BrainWAVE stimulator code. These files contain code to generate and play flicker sensory stimulation with an Arduino Uno or NIDAQ BrainWAVE stimulator device. Download Extended Data 1, ZIP file. [file enu-eN-OTM-0257-22-s05.zip › Code_FliCkER/Code_FliCkER/GUI_FliCkER/functions_and_parameters/FlickerMemoryTask/imageset_1a/Set1_016a.jpg]

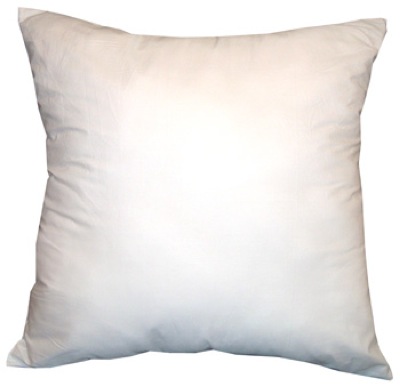

Supplement: Extended Data 1 — BrainWAVE stimulator code. These files contain code to generate and play flicker sensory stimulation with an Arduino Uno or NIDAQ BrainWAVE stimulator device. Download Extended Data 1, ZIP file. [file enu-eN-OTM-0257-22-s05.zip › Code_FliCkER/Code_FliCkER/GUI_FliCkER/functions_and_parameters/FlickerMemoryTask/imageset_1a/Set1_017a.jpg]

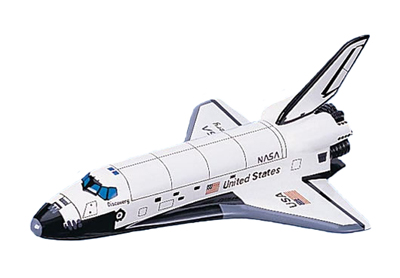

Supplement: Extended Data 1 — BrainWAVE stimulator code. These files contain code to generate and play flicker sensory stimulation with an Arduino Uno or NIDAQ BrainWAVE stimulator device. Download Extended Data 1, ZIP file. [file enu-eN-OTM-0257-22-s05.zip › Code_FliCkER/Code_FliCkER/GUI_FliCkER/functions_and_parameters/FlickerMemoryTask/imageset_1a/Set1_018a.jpg]

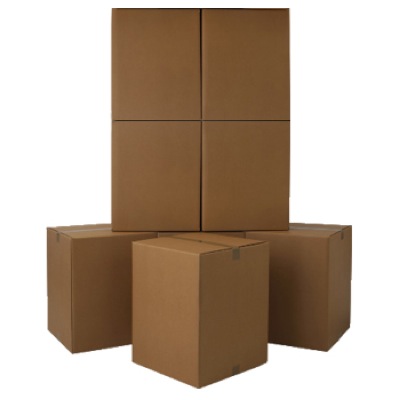

Supplement: Extended Data 1 — BrainWAVE stimulator code. These files contain code to generate and play flicker sensory stimulation with an Arduino Uno or NIDAQ BrainWAVE stimulator device. Download Extended Data 1, ZIP file. [file enu-eN-OTM-0257-22-s05.zip › Code_FliCkER/Code_FliCkER/GUI_FliCkER/functions_and_parameters/FlickerMemoryTask/imageset_1a/Set1_020a.jpg]

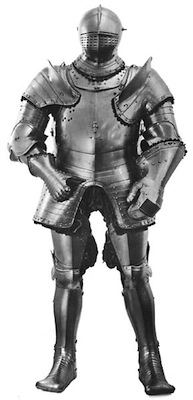

Supplement: Extended Data 1 — BrainWAVE stimulator code. These files contain code to generate and play flicker sensory stimulation with an Arduino Uno or NIDAQ BrainWAVE stimulator device. Download Extended Data 1, ZIP file. [file enu-eN-OTM-0257-22-s05.zip › Code_FliCkER/Code_FliCkER/GUI_FliCkER/functions_and_parameters/FlickerMemoryTask/imageset_1a/Set1_021a.jpg]

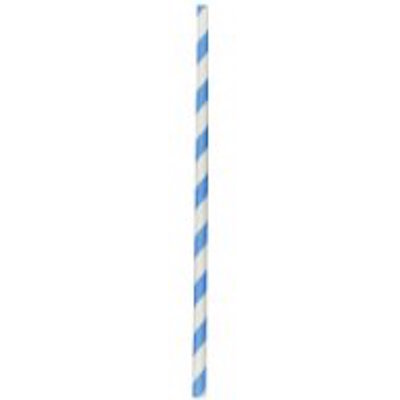

Supplement: Extended Data 1 — BrainWAVE stimulator code. These files contain code to generate and play flicker sensory stimulation with an Arduino Uno or NIDAQ BrainWAVE stimulator device. Download Extended Data 1, ZIP file. [file enu-eN-OTM-0257-22-s05.zip › Code_FliCkER/Code_FliCkER/GUI_FliCkER/functions_and_parameters/FlickerMemoryTask/imageset_1a/Set1_022a.jpg]

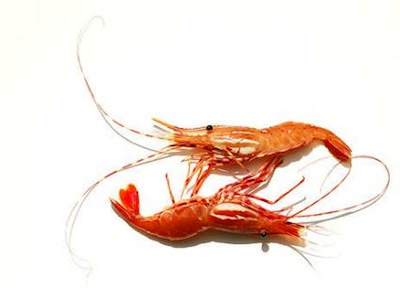

Supplement: Extended Data 1 — BrainWAVE stimulator code. These files contain code to generate and play flicker sensory stimulation with an Arduino Uno or NIDAQ BrainWAVE stimulator device. Download Extended Data 1, ZIP file. [file enu-eN-OTM-0257-22-s05.zip › Code_FliCkER/Code_FliCkER/GUI_FliCkER/functions_and_parameters/FlickerMemoryTask/imageset_1a/Set1_023a.jpg]

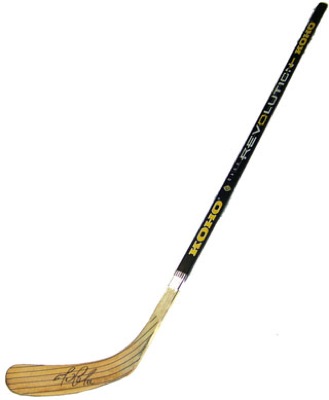

Supplement: Extended Data 1 — BrainWAVE stimulator code. These files contain code to generate and play flicker sensory stimulation with an Arduino Uno or NIDAQ BrainWAVE stimulator device. Download Extended Data 1, ZIP file. [file enu-eN-OTM-0257-22-s05.zip › Code_FliCkER/Code_FliCkER/GUI_FliCkER/functions_and_parameters/FlickerMemoryTask/imageset_1a/Set1_024a.jpg]

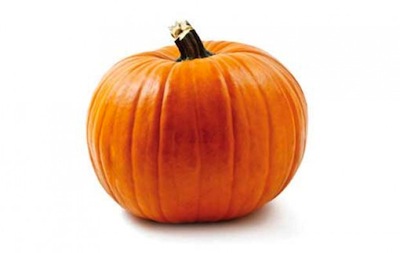

Supplement: Extended Data 1 — BrainWAVE stimulator code. These files contain code to generate and play flicker sensory stimulation with an Arduino Uno or NIDAQ BrainWAVE stimulator device. Download Extended Data 1, ZIP file. [file enu-eN-OTM-0257-22-s05.zip › Code_FliCkER/Code_FliCkER/GUI_FliCkER/functions_and_parameters/FlickerMemoryTask/imageset_1a/Set1_025a.jpg]

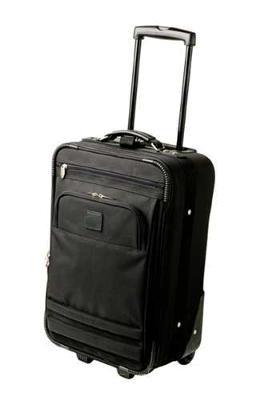

Supplement: Extended Data 1 — BrainWAVE stimulator code. These files contain code to generate and play flicker sensory stimulation with an Arduino Uno or NIDAQ BrainWAVE stimulator device. Download Extended Data 1, ZIP file. [file enu-eN-OTM-0257-22-s05.zip › Code_FliCkER/Code_FliCkER/GUI_FliCkER/functions_and_parameters/FlickerMemoryTask/imageset_1a/Set1_026a.jpg]

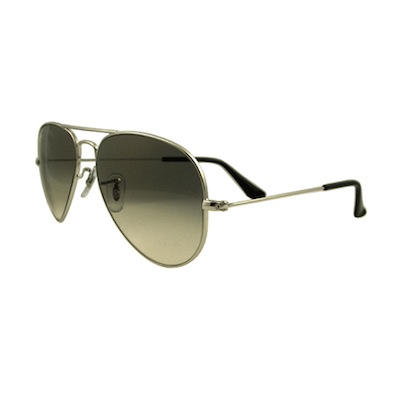

Supplement: Extended Data 1 — BrainWAVE stimulator code. These files contain code to generate and play flicker sensory stimulation with an Arduino Uno or NIDAQ BrainWAVE stimulator device. Download Extended Data 1, ZIP file. [file enu-eN-OTM-0257-22-s05.zip › Code_FliCkER/Code_FliCkER/GUI_FliCkER/functions_and_parameters/FlickerMemoryTask/imageset_1a/Set1_027a.jpg]

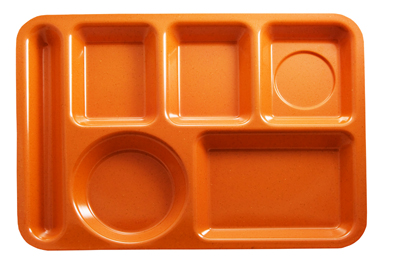

Supplement: Extended Data 1 — BrainWAVE stimulator code. These files contain code to generate and play flicker sensory stimulation with an Arduino Uno or NIDAQ BrainWAVE stimulator device. Download Extended Data 1, ZIP file. [file enu-eN-OTM-0257-22-s05.zip › Code_FliCkER/Code_FliCkER/GUI_FliCkER/functions_and_parameters/FlickerMemoryTask/imageset_1a/Set1_028a.jpg]

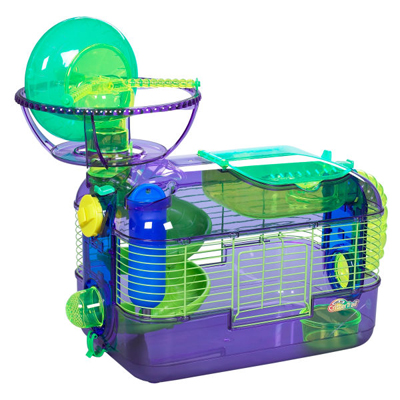

Supplement: Extended Data 1 — BrainWAVE stimulator code. These files contain code to generate and play flicker sensory stimulation with an Arduino Uno or NIDAQ BrainWAVE stimulator device. Download Extended Data 1, ZIP file. [file enu-eN-OTM-0257-22-s05.zip › Code_FliCkER/Code_FliCkER/GUI_FliCkER/functions_and_parameters/FlickerMemoryTask/imageset_1a/Set1_029a.jpg]

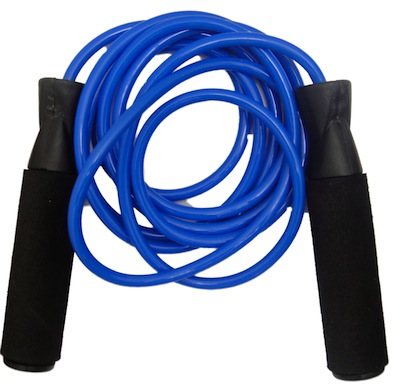

Supplement: Extended Data 1 — BrainWAVE stimulator code. These files contain code to generate and play flicker sensory stimulation with an Arduino Uno or NIDAQ BrainWAVE stimulator device. Download Extended Data 1, ZIP file. [file enu-eN-OTM-0257-22-s05.zip › Code_FliCkER/Code_FliCkER/GUI_FliCkER/functions_and_parameters/FlickerMemoryTask/imageset_1a/Set1_030a.jpg]

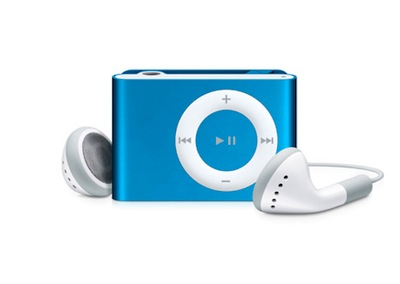

Supplement: Extended Data 1 — BrainWAVE stimulator code. These files contain code to generate and play flicker sensory stimulation with an Arduino Uno or NIDAQ BrainWAVE stimulator device. Download Extended Data 1, ZIP file. [file enu-eN-OTM-0257-22-s05.zip › Code_FliCkER/Code_FliCkER/GUI_FliCkER/functions_and_parameters/FlickerMemoryTask/imageset_1a/Set1_031a.jpg]

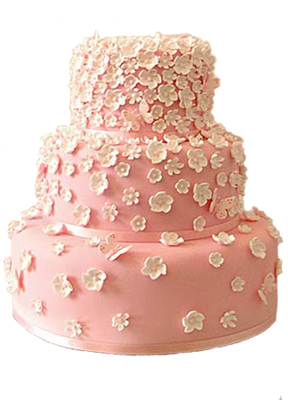

Supplement: Extended Data 1 — BrainWAVE stimulator code. These files contain code to generate and play flicker sensory stimulation with an Arduino Uno or NIDAQ BrainWAVE stimulator device. Download Extended Data 1, ZIP file. [file enu-eN-OTM-0257-22-s05.zip › Code_FliCkER/Code_FliCkER/GUI_FliCkER/functions_and_parameters/FlickerMemoryTask/imageset_1a/Set1_032a.jpg]

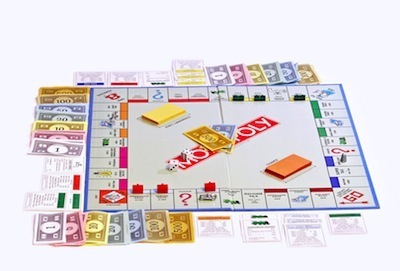

Supplement: Extended Data 1 — BrainWAVE stimulator code. These files contain code to generate and play flicker sensory stimulation with an Arduino Uno or NIDAQ BrainWAVE stimulator device. Download Extended Data 1, ZIP file. [file enu-eN-OTM-0257-22-s05.zip › Code_FliCkER/Code_FliCkER/GUI_FliCkER/functions_and_parameters/FlickerMemoryTask/imageset_1a/Set1_033a.jpg]

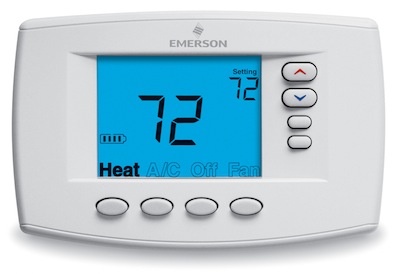

Supplement: Extended Data 1 — BrainWAVE stimulator code. These files contain code to generate and play flicker sensory stimulation with an Arduino Uno or NIDAQ BrainWAVE stimulator device. Download Extended Data 1, ZIP file. [file enu-eN-OTM-0257-22-s05.zip › Code_FliCkER/Code_FliCkER/GUI_FliCkER/functions_and_parameters/FlickerMemoryTask/imageset_1a/Set1_034a.jpg]

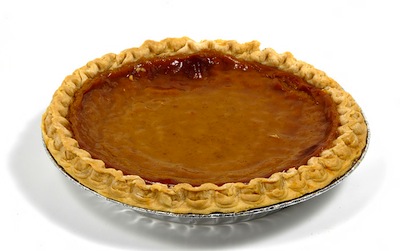

Supplement: Extended Data 1 — BrainWAVE stimulator code. These files contain code to generate and play flicker sensory stimulation with an Arduino Uno or NIDAQ BrainWAVE stimulator device. Download Extended Data 1, ZIP file. [file enu-eN-OTM-0257-22-s05.zip › Code_FliCkER/Code_FliCkER/GUI_FliCkER/functions_and_parameters/FlickerMemoryTask/imageset_1a/Set1_035a.jpg]

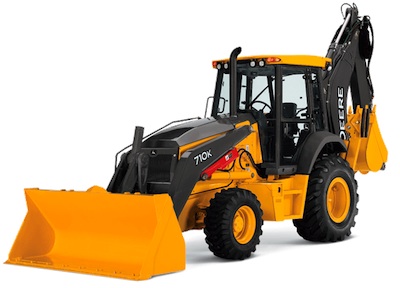

Supplement: Extended Data 1 — BrainWAVE stimulator code. These files contain code to generate and play flicker sensory stimulation with an Arduino Uno or NIDAQ BrainWAVE stimulator device. Download Extended Data 1, ZIP file. [file enu-eN-OTM-0257-22-s05.zip › Code_FliCkER/Code_FliCkER/GUI_FliCkER/functions_and_parameters/FlickerMemoryTask/imageset_1a/Set1_036a.jpg]

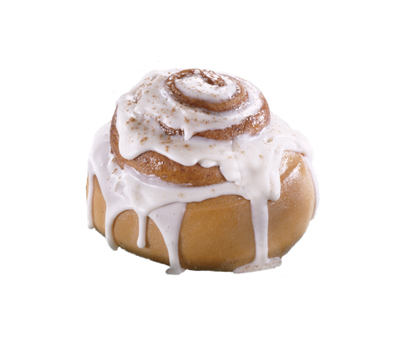

Supplement: Extended Data 1 — BrainWAVE stimulator code. These files contain code to generate and play flicker sensory stimulation with an Arduino Uno or NIDAQ BrainWAVE stimulator device. Download Extended Data 1, ZIP file. [file enu-eN-OTM-0257-22-s05.zip › Code_FliCkER/Code_FliCkER/GUI_FliCkER/functions_and_parameters/FlickerMemoryTask/imageset_1a/Set1_038a.jpg]

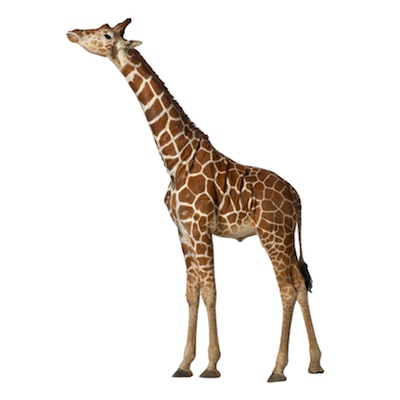

Supplement: Extended Data 1 — BrainWAVE stimulator code. These files contain code to generate and play flicker sensory stimulation with an Arduino Uno or NIDAQ BrainWAVE stimulator device. Download Extended Data 1, ZIP file. [file enu-eN-OTM-0257-22-s05.zip › Code_FliCkER/Code_FliCkER/GUI_FliCkER/functions_and_parameters/FlickerMemoryTask/imageset_1a/Set1_039a.jpg]

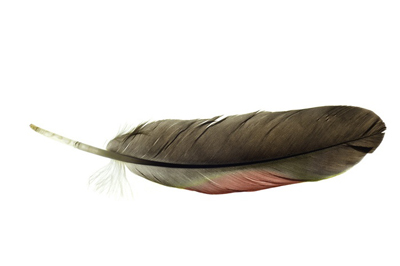

Supplement: Extended Data 1 — BrainWAVE stimulator code. These files contain code to generate and play flicker sensory stimulation with an Arduino Uno or NIDAQ BrainWAVE stimulator device. Download Extended Data 1, ZIP file. [file enu-eN-OTM-0257-22-s05.zip › Code_FliCkER/Code_FliCkER/GUI_FliCkER/functions_and_parameters/FlickerMemoryTask/imageset_1a/Set1_040a.jpg]

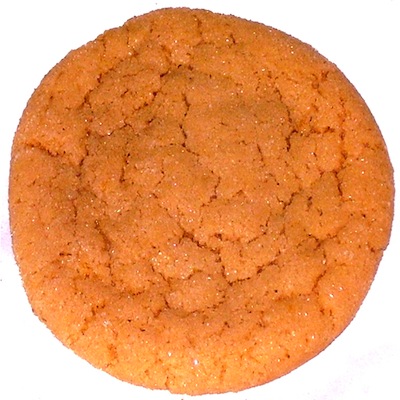

Supplement: Extended Data 1 — BrainWAVE stimulator code. These files contain code to generate and play flicker sensory stimulation with an Arduino Uno or NIDAQ BrainWAVE stimulator device. Download Extended Data 1, ZIP file. [file enu-eN-OTM-0257-22-s05.zip › Code_FliCkER/Code_FliCkER/GUI_FliCkER/functions_and_parameters/FlickerMemoryTask/imageset_1a/Set1_041a.jpg]

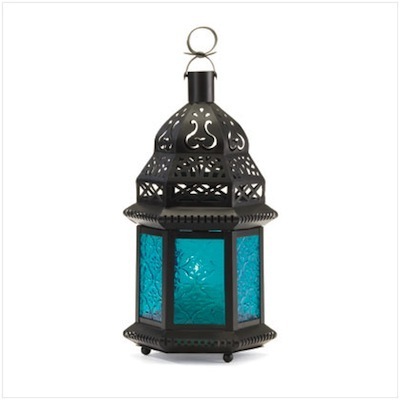

Supplement: Extended Data 1 — BrainWAVE stimulator code. These files contain code to generate and play flicker sensory stimulation with an Arduino Uno or NIDAQ BrainWAVE stimulator device. Download Extended Data 1, ZIP file. [file enu-eN-OTM-0257-22-s05.zip › Code_FliCkER/Code_FliCkER/GUI_FliCkER/functions_and_parameters/FlickerMemoryTask/imageset_1a/Set1_042a.jpg]

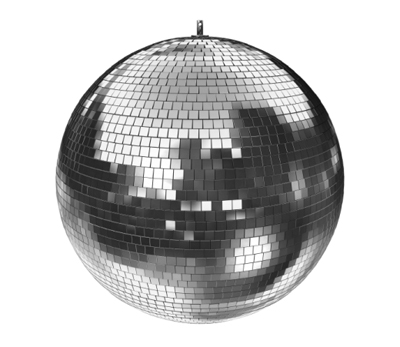

Supplement: Extended Data 1 — BrainWAVE stimulator code. These files contain code to generate and play flicker sensory stimulation with an Arduino Uno or NIDAQ BrainWAVE stimulator device. Download Extended Data 1, ZIP file. [file enu-eN-OTM-0257-22-s05.zip › Code_FliCkER/Code_FliCkER/GUI_FliCkER/functions_and_parameters/FlickerMemoryTask/imageset_1a/Set1_043a.jpg]

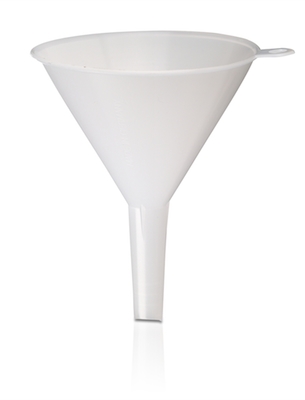

Supplement: Extended Data 1 — BrainWAVE stimulator code. These files contain code to generate and play flicker sensory stimulation with an Arduino Uno or NIDAQ BrainWAVE stimulator device. Download Extended Data 1, ZIP file. [file enu-eN-OTM-0257-22-s05.zip › Code_FliCkER/Code_FliCkER/GUI_FliCkER/functions_and_parameters/FlickerMemoryTask/imageset_1a/Set1_044a.jpg]

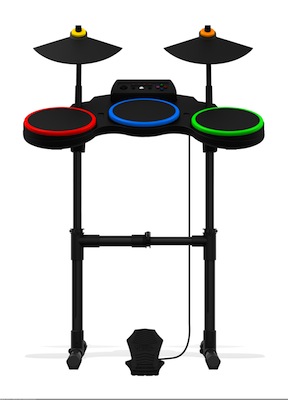

Supplement: Extended Data 1 — BrainWAVE stimulator code. These files contain code to generate and play flicker sensory stimulation with an Arduino Uno or NIDAQ BrainWAVE stimulator device. Download Extended Data 1, ZIP file. [file enu-eN-OTM-0257-22-s05.zip › Code_FliCkER/Code_FliCkER/GUI_FliCkER/functions_and_parameters/FlickerMemoryTask/imageset_1a/Set1_045a.jpg]

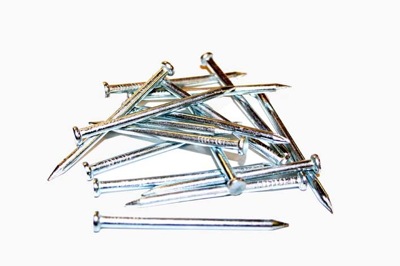

Supplement: Extended Data 1 — BrainWAVE stimulator code. These files contain code to generate and play flicker sensory stimulation with an Arduino Uno or NIDAQ BrainWAVE stimulator device. Download Extended Data 1, ZIP file. [file enu-eN-OTM-0257-22-s05.zip › Code_FliCkER/Code_FliCkER/GUI_FliCkER/functions_and_parameters/FlickerMemoryTask/imageset_1a/Set1_046a.jpg]

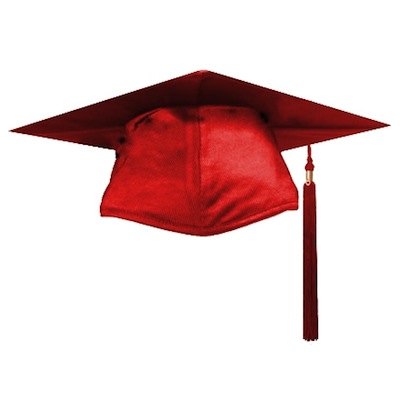

Supplement: Extended Data 1 — BrainWAVE stimulator code. These files contain code to generate and play flicker sensory stimulation with an Arduino Uno or NIDAQ BrainWAVE stimulator device. Download Extended Data 1, ZIP file. [file enu-eN-OTM-0257-22-s05.zip › Code_FliCkER/Code_FliCkER/GUI_FliCkER/functions_and_parameters/FlickerMemoryTask/imageset_1a/Set1_047a.jpg]

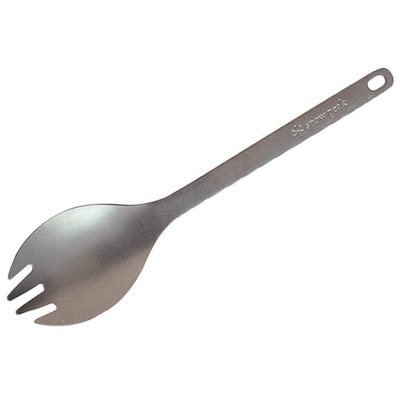

Supplement: Extended Data 1 — BrainWAVE stimulator code. These files contain code to generate and play flicker sensory stimulation with an Arduino Uno or NIDAQ BrainWAVE stimulator device. Download Extended Data 1, ZIP file. [file enu-eN-OTM-0257-22-s05.zip › Code_FliCkER/Code_FliCkER/GUI_FliCkER/functions_and_parameters/FlickerMemoryTask/imageset_1a/Set1_048a.jpg]

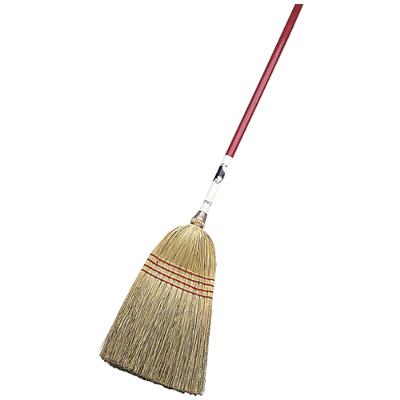

Supplement: Extended Data 1 — BrainWAVE stimulator code. These files contain code to generate and play flicker sensory stimulation with an Arduino Uno or NIDAQ BrainWAVE stimulator device. Download Extended Data 1, ZIP file. [file enu-eN-OTM-0257-22-s05.zip › Code_FliCkER/Code_FliCkER/GUI_FliCkER/functions_and_parameters/FlickerMemoryTask/imageset_1a/Set1_049a.jpg]

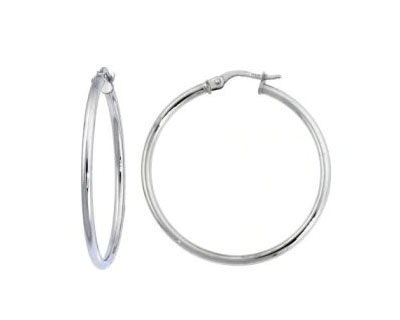

Supplement: Extended Data 1 — BrainWAVE stimulator code. These files contain code to generate and play flicker sensory stimulation with an Arduino Uno or NIDAQ BrainWAVE stimulator device. Download Extended Data 1, ZIP file. [file enu-eN-OTM-0257-22-s05.zip › Code_FliCkER/Code_FliCkER/GUI_FliCkER/functions_and_parameters/FlickerMemoryTask/imageset_1a/Set1_050a.jpg]

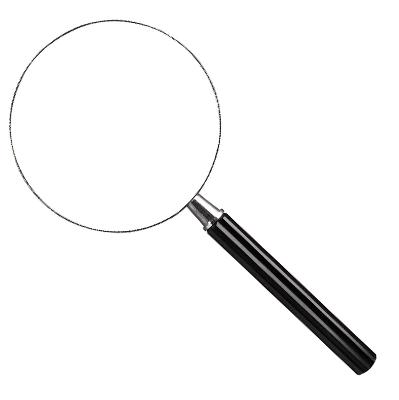

Supplement: Extended Data 1 — BrainWAVE stimulator code. These files contain code to generate and play flicker sensory stimulation with an Arduino Uno or NIDAQ BrainWAVE stimulator device. Download Extended Data 1, ZIP file. [file enu-eN-OTM-0257-22-s05.zip › Code_FliCkER/Code_FliCkER/GUI_FliCkER/functions_and_parameters/FlickerMemoryTask/imageset_1a/Set1_051a.jpg]

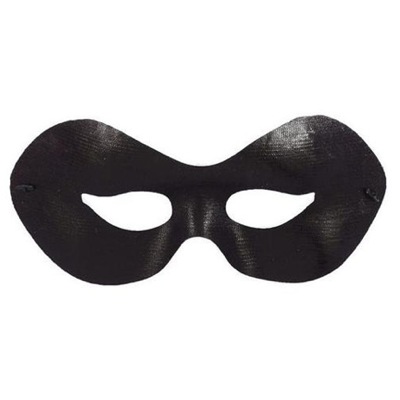

Supplement: Extended Data 1 — BrainWAVE stimulator code. These files contain code to generate and play flicker sensory stimulation with an Arduino Uno or NIDAQ BrainWAVE stimulator device. Download Extended Data 1, ZIP file. [file enu-eN-OTM-0257-22-s05.zip › Code_FliCkER/Code_FliCkER/GUI_FliCkER/functions_and_parameters/FlickerMemoryTask/imageset_1a/Set1_052a.jpg]

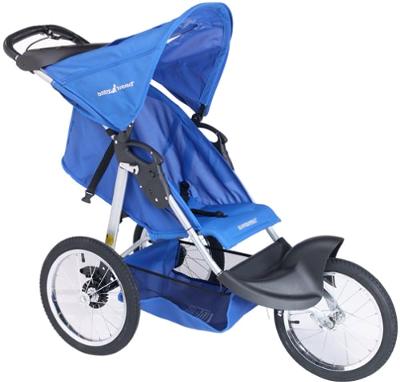

Supplement: Extended Data 1 — BrainWAVE stimulator code. These files contain code to generate and play flicker sensory stimulation with an Arduino Uno or NIDAQ BrainWAVE stimulator device. Download Extended Data 1, ZIP file. [file enu-eN-OTM-0257-22-s05.zip › Code_FliCkER/Code_FliCkER/GUI_FliCkER/functions_and_parameters/FlickerMemoryTask/imageset_1a/Set1_053a.jpg]

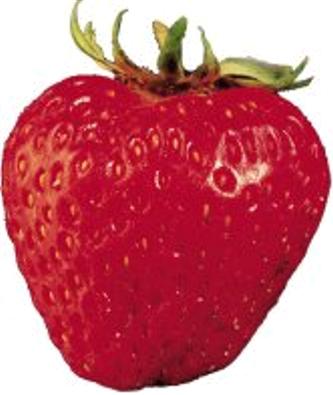

Supplement: Extended Data 1 — BrainWAVE stimulator code. These files contain code to generate and play flicker sensory stimulation with an Arduino Uno or NIDAQ BrainWAVE stimulator device. Download Extended Data 1, ZIP file. [file enu-eN-OTM-0257-22-s05.zip › Code_FliCkER/Code_FliCkER/GUI_FliCkER/functions_and_parameters/FlickerMemoryTask/imageset_1a/Set1_054a.jpg]

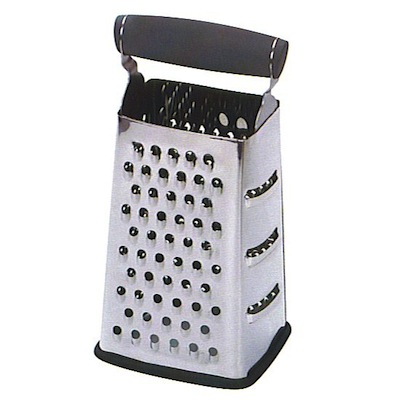

Supplement: Extended Data 1 — BrainWAVE stimulator code. These files contain code to generate and play flicker sensory stimulation with an Arduino Uno or NIDAQ BrainWAVE stimulator device. Download Extended Data 1, ZIP file. [file enu-eN-OTM-0257-22-s05.zip › Code_FliCkER/Code_FliCkER/GUI_FliCkER/functions_and_parameters/FlickerMemoryTask/imageset_1a/Set1_055a.jpg]

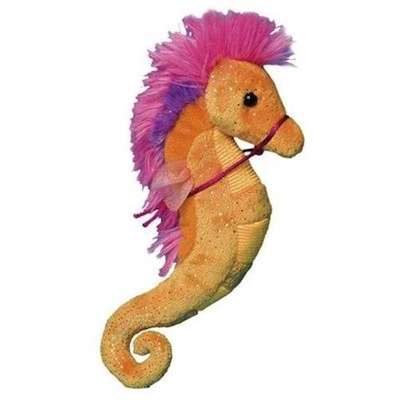

Supplement: Extended Data 1 — BrainWAVE stimulator code. These files contain code to generate and play flicker sensory stimulation with an Arduino Uno or NIDAQ BrainWAVE stimulator device. Download Extended Data 1, ZIP file. [file enu-eN-OTM-0257-22-s05.zip › Code_FliCkER/Code_FliCkER/GUI_FliCkER/functions_and_parameters/FlickerMemoryTask/imageset_1a/Set1_056a.jpg]

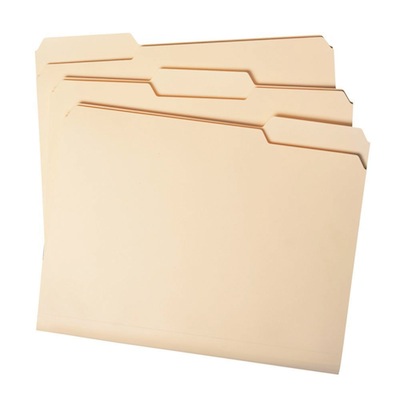

Supplement: Extended Data 1 — BrainWAVE stimulator code. These files contain code to generate and play flicker sensory stimulation with an Arduino Uno or NIDAQ BrainWAVE stimulator device. Download Extended Data 1, ZIP file. [file enu-eN-OTM-0257-22-s05.zip › Code_FliCkER/Code_FliCkER/GUI_FliCkER/functions_and_parameters/FlickerMemoryTask/imageset_1a/Set1_057a.jpg]

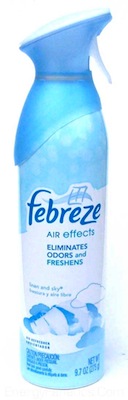

Supplement: Extended Data 1 — BrainWAVE stimulator code. These files contain code to generate and play flicker sensory stimulation with an Arduino Uno or NIDAQ BrainWAVE stimulator device. Download Extended Data 1, ZIP file. [file enu-eN-OTM-0257-22-s05.zip › Code_FliCkER/Code_FliCkER/GUI_FliCkER/functions_and_parameters/FlickerMemoryTask/imageset_1a/Set1_058a.jpg]

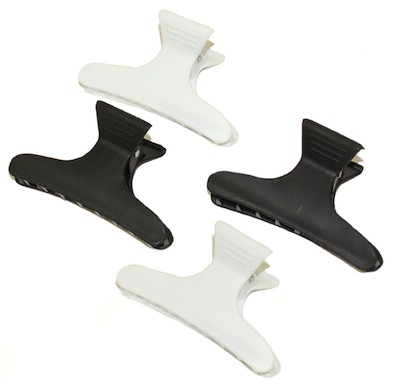

Supplement: Extended Data 1 — BrainWAVE stimulator code. These files contain code to generate and play flicker sensory stimulation with an Arduino Uno or NIDAQ BrainWAVE stimulator device. Download Extended Data 1, ZIP file. [file enu-eN-OTM-0257-22-s05.zip › Code_FliCkER/Code_FliCkER/GUI_FliCkER/functions_and_parameters/FlickerMemoryTask/imageset_1a/Set1_059a.jpg]

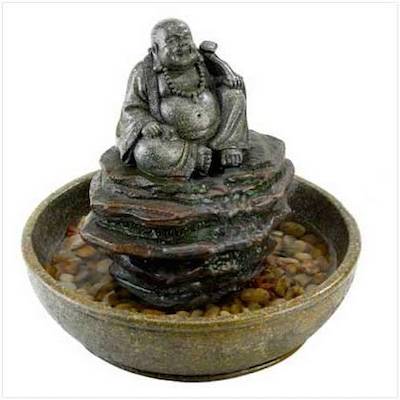

Supplement: Extended Data 1 — BrainWAVE stimulator code. These files contain code to generate and play flicker sensory stimulation with an Arduino Uno or NIDAQ BrainWAVE stimulator device. Download Extended Data 1, ZIP file. [file enu-eN-OTM-0257-22-s05.zip › Code_FliCkER/Code_FliCkER/GUI_FliCkER/functions_and_parameters/FlickerMemoryTask/imageset_1a/Set1_060a.jpg]

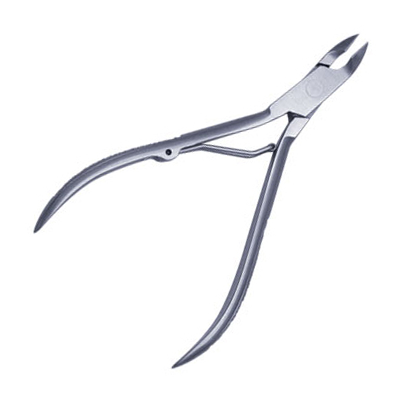

Supplement: Extended Data 1 — BrainWAVE stimulator code. These files contain code to generate and play flicker sensory stimulation with an Arduino Uno or NIDAQ BrainWAVE stimulator device. Download Extended Data 1, ZIP file. [file enu-eN-OTM-0257-22-s05.zip › Code_FliCkER/Code_FliCkER/GUI_FliCkER/functions_and_parameters/FlickerMemoryTask/imageset_1a/Set1_061a.jpg]

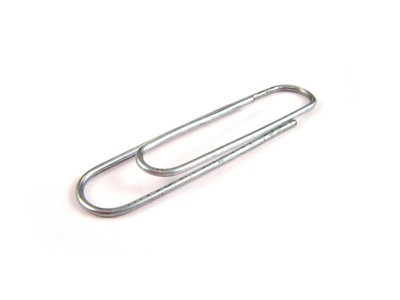

Supplement: Extended Data 1 — BrainWAVE stimulator code. These files contain code to generate and play flicker sensory stimulation with an Arduino Uno or NIDAQ BrainWAVE stimulator device. Download Extended Data 1, ZIP file. [file enu-eN-OTM-0257-22-s05.zip › Code_FliCkER/Code_FliCkER/GUI_FliCkER/functions_and_parameters/FlickerMemoryTask/imageset_1a/Set1_062a.jpg]

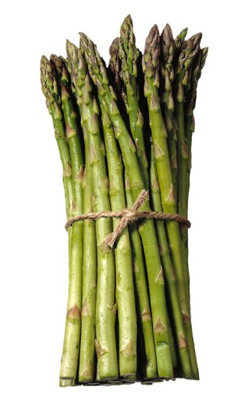

Supplement: Extended Data 1 — BrainWAVE stimulator code. These files contain code to generate and play flicker sensory stimulation with an Arduino Uno or NIDAQ BrainWAVE stimulator device. Download Extended Data 1, ZIP file. [file enu-eN-OTM-0257-22-s05.zip › Code_FliCkER/Code_FliCkER/GUI_FliCkER/functions_and_parameters/FlickerMemoryTask/imageset_1a/Set1_063a.jpg]

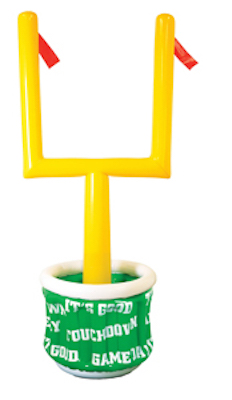

Supplement: Extended Data 1 — BrainWAVE stimulator code. These files contain code to generate and play flicker sensory stimulation with an Arduino Uno or NIDAQ BrainWAVE stimulator device. Download Extended Data 1, ZIP file. [file enu-eN-OTM-0257-22-s05.zip › Code_FliCkER/Code_FliCkER/GUI_FliCkER/functions_and_parameters/FlickerMemoryTask/imageset_1a/Set1_064a.jpg]

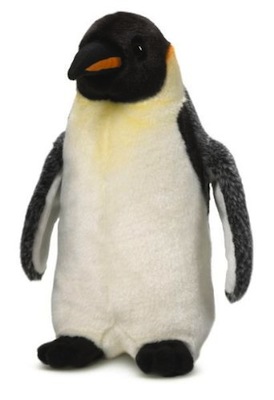

Supplement: Extended Data 1 — BrainWAVE stimulator code. These files contain code to generate and play flicker sensory stimulation with an Arduino Uno or NIDAQ BrainWAVE stimulator device. Download Extended Data 1, ZIP file. [file enu-eN-OTM-0257-22-s05.zip › Code_FliCkER/Code_FliCkER/GUI_FliCkER/functions_and_parameters/FlickerMemoryTask/imageset_1a/Set1_065a.jpg]

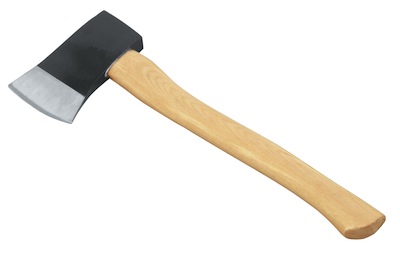

Supplement: Extended Data 1 — BrainWAVE stimulator code. These files contain code to generate and play flicker sensory stimulation with an Arduino Uno or NIDAQ BrainWAVE stimulator device. Download Extended Data 1, ZIP file. [file enu-eN-OTM-0257-22-s05.zip › Code_FliCkER/Code_FliCkER/GUI_FliCkER/functions_and_parameters/FlickerMemoryTask/imageset_1a/Set1_066a.jpg]

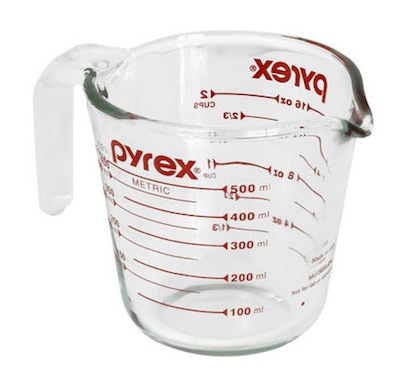

Supplement: Extended Data 1 — BrainWAVE stimulator code. These files contain code to generate and play flicker sensory stimulation with an Arduino Uno or NIDAQ BrainWAVE stimulator device. Download Extended Data 1, ZIP file. [file enu-eN-OTM-0257-22-s05.zip › Code_FliCkER/Code_FliCkER/GUI_FliCkER/functions_and_parameters/FlickerMemoryTask/imageset_1a/Set1_067a.jpg]

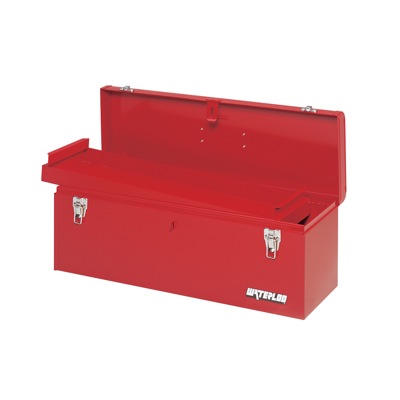

Supplement: Extended Data 1 — BrainWAVE stimulator code. These files contain code to generate and play flicker sensory stimulation with an Arduino Uno or NIDAQ BrainWAVE stimulator device. Download Extended Data 1, ZIP file. [file enu-eN-OTM-0257-22-s05.zip › Code_FliCkER/Code_FliCkER/GUI_FliCkER/functions_and_parameters/FlickerMemoryTask/imageset_1a/Set1_068a.jpg]

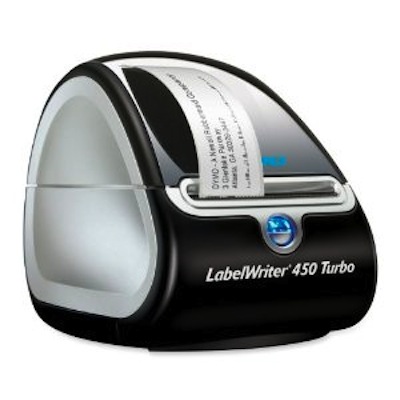

Supplement: Extended Data 1 — BrainWAVE stimulator code. These files contain code to generate and play flicker sensory stimulation with an Arduino Uno or NIDAQ BrainWAVE stimulator device. Download Extended Data 1, ZIP file. [file enu-eN-OTM-0257-22-s05.zip › Code_FliCkER/Code_FliCkER/GUI_FliCkER/functions_and_parameters/FlickerMemoryTask/imageset_1a/Set1_069a.jpg]

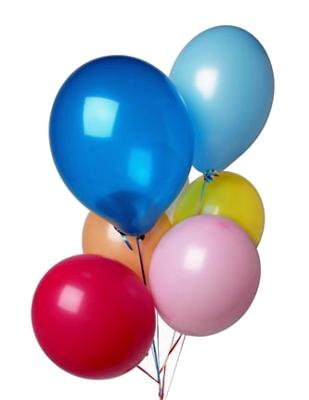

Supplement: Extended Data 1 — BrainWAVE stimulator code. These files contain code to generate and play flicker sensory stimulation with an Arduino Uno or NIDAQ BrainWAVE stimulator device. Download Extended Data 1, ZIP file. [file enu-eN-OTM-0257-22-s05.zip › Code_FliCkER/Code_FliCkER/GUI_FliCkER/functions_and_parameters/FlickerMemoryTask/imageset_1a/Set1_070a.jpg]

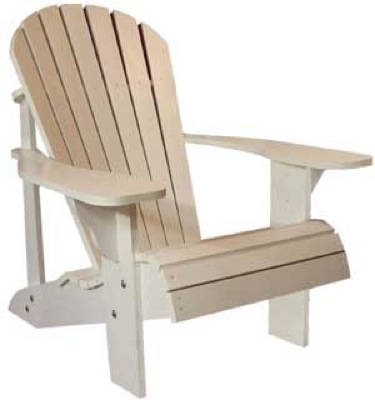

Supplement: Extended Data 1 — BrainWAVE stimulator code. These files contain code to generate and play flicker sensory stimulation with an Arduino Uno or NIDAQ BrainWAVE stimulator device. Download Extended Data 1, ZIP file. [file enu-eN-OTM-0257-22-s05.zip › Code_FliCkER/Code_FliCkER/GUI_FliCkER/functions_and_parameters/FlickerMemoryTask/imageset_1a/Set1_071a.jpg]

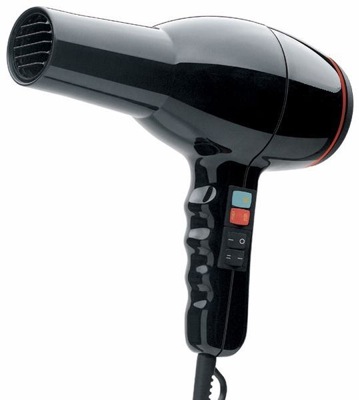

Supplement: Extended Data 1 — BrainWAVE stimulator code. These files contain code to generate and play flicker sensory stimulation with an Arduino Uno or NIDAQ BrainWAVE stimulator device. Download Extended Data 1, ZIP file. [file enu-eN-OTM-0257-22-s05.zip › Code_FliCkER/Code_FliCkER/GUI_FliCkER/functions_and_parameters/FlickerMemoryTask/imageset_1a/Set1_072a.jpg]

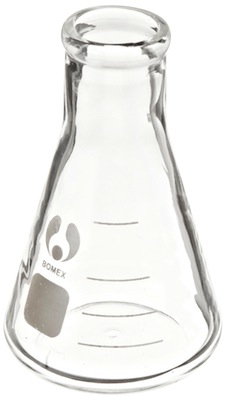

Supplement: Extended Data 1 — BrainWAVE stimulator code. These files contain code to generate and play flicker sensory stimulation with an Arduino Uno or NIDAQ BrainWAVE stimulator device. Download Extended Data 1, ZIP file. [file enu-eN-OTM-0257-22-s05.zip › Code_FliCkER/Code_FliCkER/GUI_FliCkER/functions_and_parameters/FlickerMemoryTask/imageset_1a/Set1_073a.jpg]

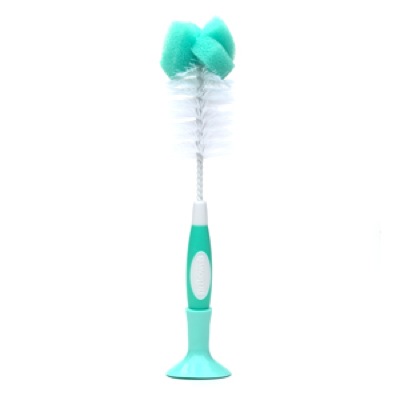

Supplement: Extended Data 1 — BrainWAVE stimulator code. These files contain code to generate and play flicker sensory stimulation with an Arduino Uno or NIDAQ BrainWAVE stimulator device. Download Extended Data 1, ZIP file. [file enu-eN-OTM-0257-22-s05.zip › Code_FliCkER/Code_FliCkER/GUI_FliCkER/functions_and_parameters/FlickerMemoryTask/imageset_1a/Set1_074a.jpg]

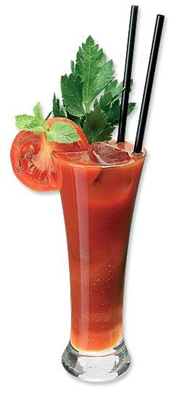

Supplement: Extended Data 1 — BrainWAVE stimulator code. These files contain code to generate and play flicker sensory stimulation with an Arduino Uno or NIDAQ BrainWAVE stimulator device. Download Extended Data 1, ZIP file. [file enu-eN-OTM-0257-22-s05.zip › Code_FliCkER/Code_FliCkER/GUI_FliCkER/functions_and_parameters/FlickerMemoryTask/imageset_1a/Set1_075a.jpg]

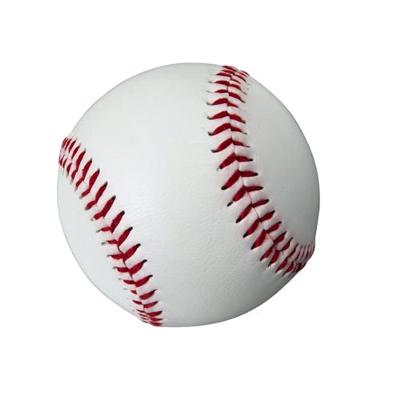

Supplement: Extended Data 1 — BrainWAVE stimulator code. These files contain code to generate and play flicker sensory stimulation with an Arduino Uno or NIDAQ BrainWAVE stimulator device. Download Extended Data 1, ZIP file. [file enu-eN-OTM-0257-22-s05.zip › Code_FliCkER/Code_FliCkER/GUI_FliCkER/functions_and_parameters/FlickerMemoryTask/imageset_1a/Set1_076a.jpg]

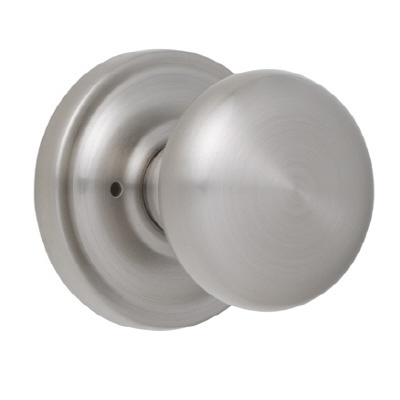

Supplement: Extended Data 1 — BrainWAVE stimulator code. These files contain code to generate and play flicker sensory stimulation with an Arduino Uno or NIDAQ BrainWAVE stimulator device. Download Extended Data 1, ZIP file. [file enu-eN-OTM-0257-22-s05.zip › Code_FliCkER/Code_FliCkER/GUI_FliCkER/functions_and_parameters/FlickerMemoryTask/imageset_1a/Set1_077a.jpg]

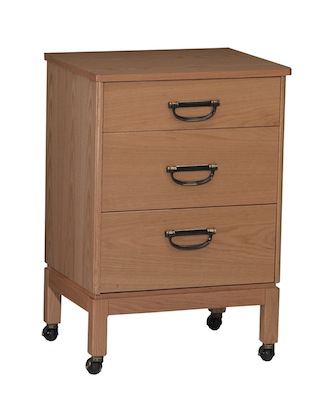

Supplement: Extended Data 1 — BrainWAVE stimulator code. These files contain code to generate and play flicker sensory stimulation with an Arduino Uno or NIDAQ BrainWAVE stimulator device. Download Extended Data 1, ZIP file. [file enu-eN-OTM-0257-22-s05.zip › Code_FliCkER/Code_FliCkER/GUI_FliCkER/functions_and_parameters/FlickerMemoryTask/imageset_1a/Set1_078a.jpg]

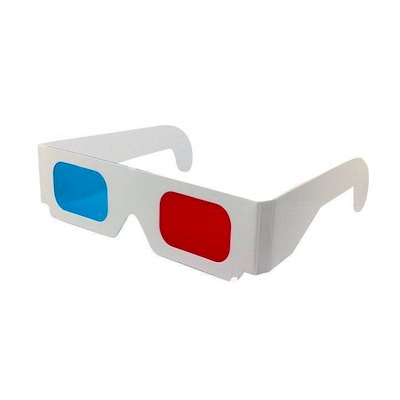

Supplement: Extended Data 1 — BrainWAVE stimulator code. These files contain code to generate and play flicker sensory stimulation with an Arduino Uno or NIDAQ BrainWAVE stimulator device. Download Extended Data 1, ZIP file. [file enu-eN-OTM-0257-22-s05.zip › Code_FliCkER/Code_FliCkER/GUI_FliCkER/functions_and_parameters/FlickerMemoryTask/imageset_1a/Set1_079a.jpg]

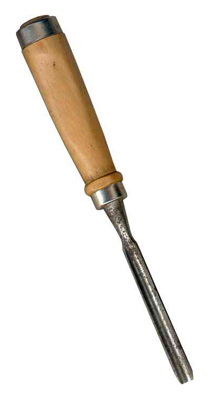

Supplement: Extended Data 1 — BrainWAVE stimulator code. These files contain code to generate and play flicker sensory stimulation with an Arduino Uno or NIDAQ BrainWAVE stimulator device. Download Extended Data 1, ZIP file. [file enu-eN-OTM-0257-22-s05.zip › Code_FliCkER/Code_FliCkER/GUI_FliCkER/functions_and_parameters/FlickerMemoryTask/imageset_1a/Set1_080a.jpg]

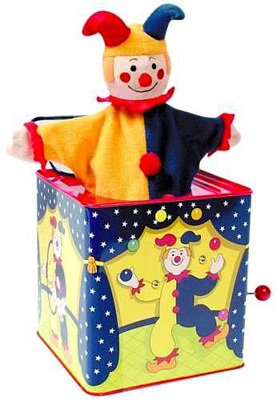

Supplement: Extended Data 1 — BrainWAVE stimulator code. These files contain code to generate and play flicker sensory stimulation with an Arduino Uno or NIDAQ BrainWAVE stimulator device. Download Extended Data 1, ZIP file. [file enu-eN-OTM-0257-22-s05.zip › Code_FliCkER/Code_FliCkER/GUI_FliCkER/functions_and_parameters/FlickerMemoryTask/imageset_1a/Set1_081a.jpg]

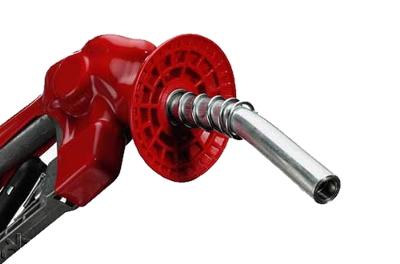

Supplement: Extended Data 1 — BrainWAVE stimulator code. These files contain code to generate and play flicker sensory stimulation with an Arduino Uno or NIDAQ BrainWAVE stimulator device. Download Extended Data 1, ZIP file. [file enu-eN-OTM-0257-22-s05.zip › Code_FliCkER/Code_FliCkER/GUI_FliCkER/functions_and_parameters/FlickerMemoryTask/imageset_1a/Set1_082a.jpg]

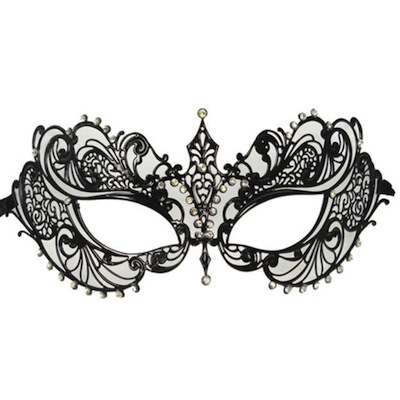

Supplement: Extended Data 1 — BrainWAVE stimulator code. These files contain code to generate and play flicker sensory stimulation with an Arduino Uno or NIDAQ BrainWAVE stimulator device. Download Extended Data 1, ZIP file. [file enu-eN-OTM-0257-22-s05.zip › Code_FliCkER/Code_FliCkER/GUI_FliCkER/functions_and_parameters/FlickerMemoryTask/imageset_1a/Set1_084a.jpg]

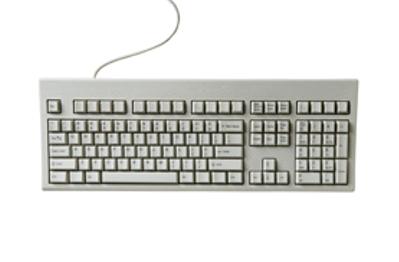

Supplement: Extended Data 1 — BrainWAVE stimulator code. These files contain code to generate and play flicker sensory stimulation with an Arduino Uno or NIDAQ BrainWAVE stimulator device. Download Extended Data 1, ZIP file. [file enu-eN-OTM-0257-22-s05.zip › Code_FliCkER/Code_FliCkER/GUI_FliCkER/functions_and_parameters/FlickerMemoryTask/imageset_1a/Set1_085a.jpg]

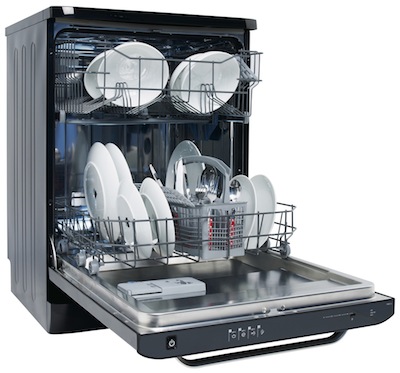

Supplement: Extended Data 1 — BrainWAVE stimulator code. These files contain code to generate and play flicker sensory stimulation with an Arduino Uno or NIDAQ BrainWAVE stimulator device. Download Extended Data 1, ZIP file. [file enu-eN-OTM-0257-22-s05.zip › Code_FliCkER/Code_FliCkER/GUI_FliCkER/functions_and_parameters/FlickerMemoryTask/imageset_1a/Set1_086a.jpg]

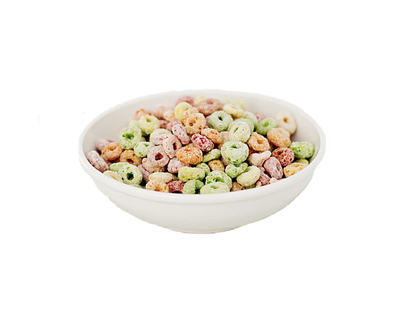

Supplement: Extended Data 1 — BrainWAVE stimulator code. These files contain code to generate and play flicker sensory stimulation with an Arduino Uno or NIDAQ BrainWAVE stimulator device. Download Extended Data 1, ZIP file. [file enu-eN-OTM-0257-22-s05.zip › Code_FliCkER/Code_FliCkER/GUI_FliCkER/functions_and_parameters/FlickerMemoryTask/imageset_1a/Set1_087a.jpg]

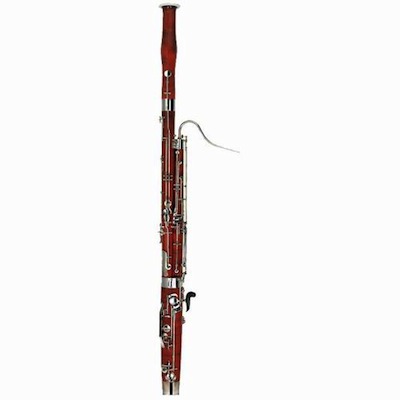

Supplement: Extended Data 1 — BrainWAVE stimulator code. These files contain code to generate and play flicker sensory stimulation with an Arduino Uno or NIDAQ BrainWAVE stimulator device. Download Extended Data 1, ZIP file. [file enu-eN-OTM-0257-22-s05.zip › Code_FliCkER/Code_FliCkER/GUI_FliCkER/functions_and_parameters/FlickerMemoryTask/imageset_1a/Set1_089a.jpg]

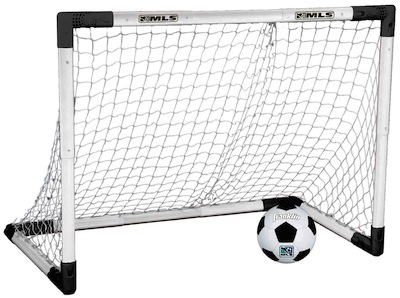

Supplement: Extended Data 1 — BrainWAVE stimulator code. These files contain code to generate and play flicker sensory stimulation with an Arduino Uno or NIDAQ BrainWAVE stimulator device. Download Extended Data 1, ZIP file. [file enu-eN-OTM-0257-22-s05.zip › Code_FliCkER/Code_FliCkER/GUI_FliCkER/functions_and_parameters/FlickerMemoryTask/imageset_1a/Set1_090a.jpg]

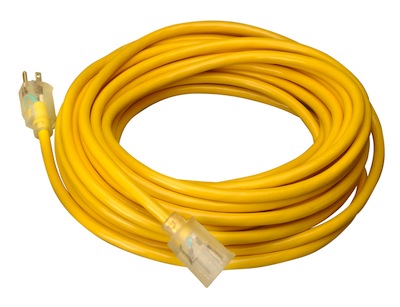

Supplement: Extended Data 1 — BrainWAVE stimulator code. These files contain code to generate and play flicker sensory stimulation with an Arduino Uno or NIDAQ BrainWAVE stimulator device. Download Extended Data 1, ZIP file. [file enu-eN-OTM-0257-22-s05.zip › Code_FliCkER/Code_FliCkER/GUI_FliCkER/functions_and_parameters/FlickerMemoryTask/imageset_1a/Set1_091a.jpg]

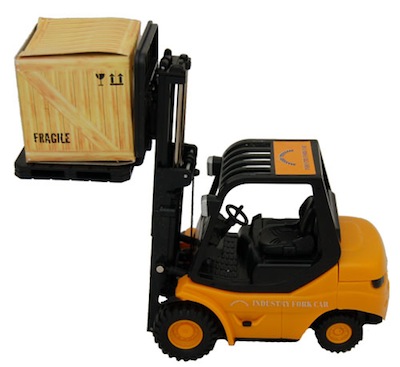

Supplement: Extended Data 1 — BrainWAVE stimulator code. These files contain code to generate and play flicker sensory stimulation with an Arduino Uno or NIDAQ BrainWAVE stimulator device. Download Extended Data 1, ZIP file. [file enu-eN-OTM-0257-22-s05.zip › Code_FliCkER/Code_FliCkER/GUI_FliCkER/functions_and_parameters/FlickerMemoryTask/imageset_1a/Set1_092a.jpg]

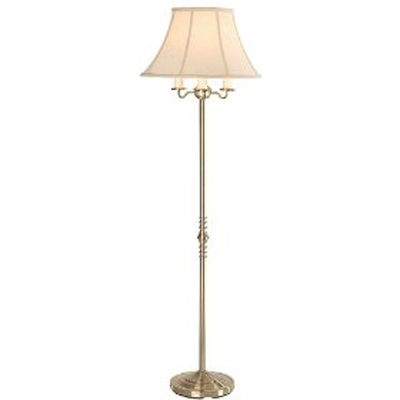

Supplement: Extended Data 1 — BrainWAVE stimulator code. These files contain code to generate and play flicker sensory stimulation with an Arduino Uno or NIDAQ BrainWAVE stimulator device. Download Extended Data 1, ZIP file. [file enu-eN-OTM-0257-22-s05.zip › Code_FliCkER/Code_FliCkER/GUI_FliCkER/functions_and_parameters/FlickerMemoryTask/imageset_1a/Set1_094a.jpg]

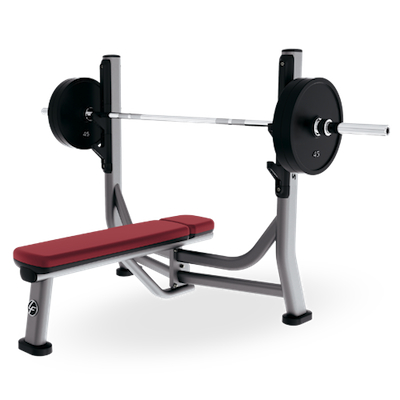

Supplement: Extended Data 1 — BrainWAVE stimulator code. These files contain code to generate and play flicker sensory stimulation with an Arduino Uno or NIDAQ BrainWAVE stimulator device. Download Extended Data 1, ZIP file. [file enu-eN-OTM-0257-22-s05.zip › Code_FliCkER/Code_FliCkER/GUI_FliCkER/functions_and_parameters/FlickerMemoryTask/imageset_1a/Set1_095a.jpg]

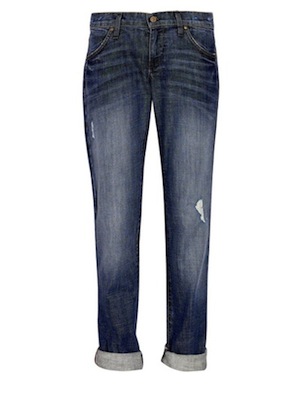

Supplement: Extended Data 1 — BrainWAVE stimulator code. These files contain code to generate and play flicker sensory stimulation with an Arduino Uno or NIDAQ BrainWAVE stimulator device. Download Extended Data 1, ZIP file. [file enu-eN-OTM-0257-22-s05.zip › Code_FliCkER/Code_FliCkER/GUI_FliCkER/functions_and_parameters/FlickerMemoryTask/imageset_1a/Set1_097a.jpg]

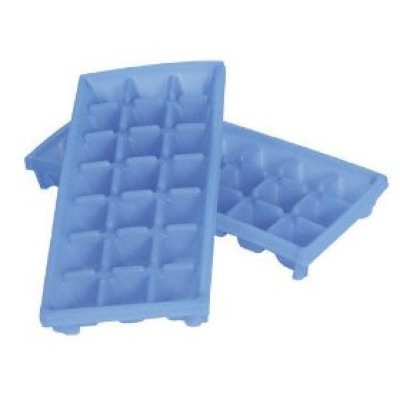

Supplement: Extended Data 1 — BrainWAVE stimulator code. These files contain code to generate and play flicker sensory stimulation with an Arduino Uno or NIDAQ BrainWAVE stimulator device. Download Extended Data 1, ZIP file. [file enu-eN-OTM-0257-22-s05.zip › Code_FliCkER/Code_FliCkER/GUI_FliCkER/functions_and_parameters/FlickerMemoryTask/imageset_1a/Set1_098a.jpg]

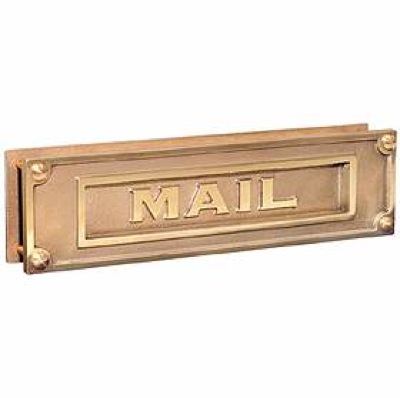

Supplement: Extended Data 1 — BrainWAVE stimulator code. These files contain code to generate and play flicker sensory stimulation with an Arduino Uno or NIDAQ BrainWAVE stimulator device. Download Extended Data 1, ZIP file. [file enu-eN-OTM-0257-22-s05.zip › Code_FliCkER/Code_FliCkER/GUI_FliCkER/functions_and_parameters/FlickerMemoryTask/imageset_1a/Set1_099a.jpg]

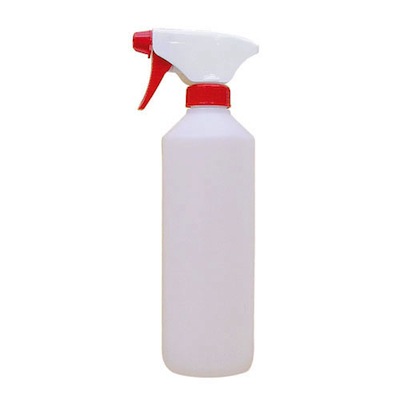

Supplement: Extended Data 1 — BrainWAVE stimulator code. These files contain code to generate and play flicker sensory stimulation with an Arduino Uno or NIDAQ BrainWAVE stimulator device. Download Extended Data 1, ZIP file. [file enu-eN-OTM-0257-22-s05.zip › Code_FliCkER/Code_FliCkER/GUI_FliCkER/functions_and_parameters/FlickerMemoryTask/imageset_1a/Set1_100a.jpg]

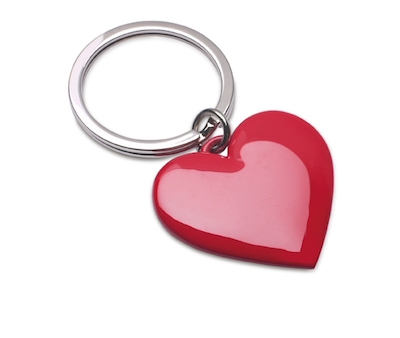

Supplement: Extended Data 1 — BrainWAVE stimulator code. These files contain code to generate and play flicker sensory stimulation with an Arduino Uno or NIDAQ BrainWAVE stimulator device. Download Extended Data 1, ZIP file. [file enu-eN-OTM-0257-22-s05.zip › Code_FliCkER/Code_FliCkER/GUI_FliCkER/functions_and_parameters/FlickerMemoryTask/imageset_1a/Set1_101a.jpg]

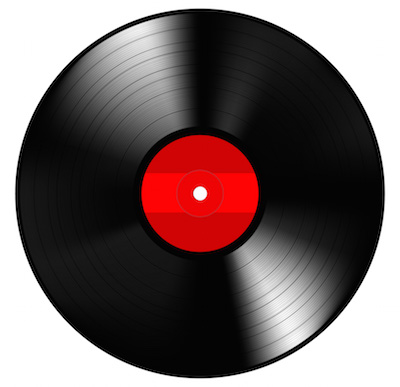

Supplement: Extended Data 1 — BrainWAVE stimulator code. These files contain code to generate and play flicker sensory stimulation with an Arduino Uno or NIDAQ BrainWAVE stimulator device. Download Extended Data 1, ZIP file. [file enu-eN-OTM-0257-22-s05.zip › Code_FliCkER/Code_FliCkER/GUI_FliCkER/functions_and_parameters/FlickerMemoryTask/imageset_1a/Set1_102a.jpg]

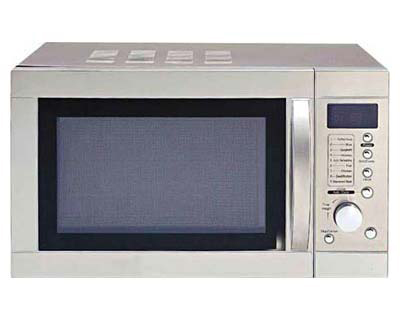

Supplement: Extended Data 1 — BrainWAVE stimulator code. These files contain code to generate and play flicker sensory stimulation with an Arduino Uno or NIDAQ BrainWAVE stimulator device. Download Extended Data 1, ZIP file. [file enu-eN-OTM-0257-22-s05.zip › Code_FliCkER/Code_FliCkER/GUI_FliCkER/functions_and_parameters/FlickerMemoryTask/imageset_1a/Set1_103a.jpg]

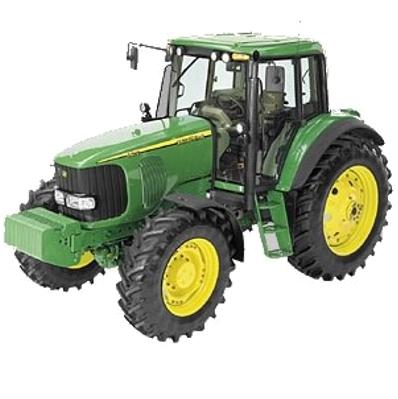

Supplement: Extended Data 1 — BrainWAVE stimulator code. These files contain code to generate and play flicker sensory stimulation with an Arduino Uno or NIDAQ BrainWAVE stimulator device. Download Extended Data 1, ZIP file. [file enu-eN-OTM-0257-22-s05.zip › Code_FliCkER/Code_FliCkER/GUI_FliCkER/functions_and_parameters/FlickerMemoryTask/imageset_1a/Set1_104a.jpg]

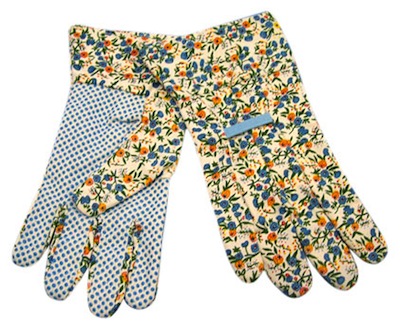

Supplement: Extended Data 1 — BrainWAVE stimulator code. These files contain code to generate and play flicker sensory stimulation with an Arduino Uno or NIDAQ BrainWAVE stimulator device. Download Extended Data 1, ZIP file. [file enu-eN-OTM-0257-22-s05.zip › Code_FliCkER/Code_FliCkER/GUI_FliCkER/functions_and_parameters/FlickerMemoryTask/imageset_1a/Set1_105a.jpg]

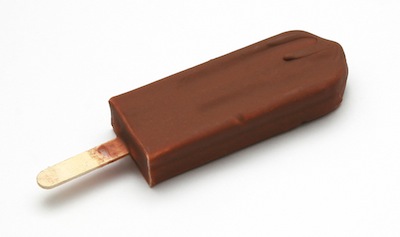

Supplement: Extended Data 1 — BrainWAVE stimulator code. These files contain code to generate and play flicker sensory stimulation with an Arduino Uno or NIDAQ BrainWAVE stimulator device. Download Extended Data 1, ZIP file. [file enu-eN-OTM-0257-22-s05.zip › Code_FliCkER/Code_FliCkER/GUI_FliCkER/functions_and_parameters/FlickerMemoryTask/imageset_1a/Set1_106a.jpg]
