# Supplementary material for: BrainWAVE: A Flexible Method for Noninvasive Stimulation of Brain Rhythms across Species
Source: eNeuro. 2023 Feb 23;10(2):ENEURO.0257-22.2022. doi: 10.1523/ENEURO.0257-22.2022 (PMC9979148; doi:10.1523/ENEURO.0257-22.2022)
Supplement: Extended Data Table 1-1 — Parts list. Components for signal generation, modulation, and sensory stimulation are needed to assemble and BrainWAVE stimulator. This table lists components that can be used and their estimated price. Download Table 1-1, DOCX file. [file enu-eN-OTM-0257-22-s02.docx]

***BrainWAVE Circuit Parts List***

| Table 1-1. *Parts List* | |  |  |  |
| --- | --- | --- | --- | --- |
|  | Part | Purpose | Part Number | Cost |
| ***Signal Generation*** *(select one)* | |  |  |  |
|  | Arduino Uno | Generates flicker signal | E.g., Arduino CC:7630049200074 | $21.90 |
|  | Data Acquisition Device | Generates flicker signal | E.g., National Instruments: USB-6212 | $1,357.00 |
| ***Signal Modulation*** | |  |  |  |
|  | n-channel MOSFET | Switches LED signal on and off | E.g., Digikey IRFZ44N | $1.02 |
|  | Eight AA Battery Holder | Supplies power to 12 V LED lights | E.g., Digikey: BH48AAW-ND | $3.24 |
|  | LED Dimmer | Allow adjustment of LED brightness | superbrightleds.com: LDK-8A | $9.95 |
|  | Audio Amplifier | Allows adjustment of audio volume | E.g., Amazon: B00ULRFQ1A | $53.99 |
| ***Visual Output*** | |  |  |  |
|  | LED Strip Lights | Delivers visual stimuli | E.g., superbrightleds.com: STN-A40K80-B3A-08B5M-12V | $28.95 |
| ***Audio Output*** | |  |  |  |
|  | Speaker | Delivers audio stimuli | Amazon: B0007L8A7M | $29.99 |
| ***Circuit Components*** | |  |  |  |
|  | 12 V ACDC Power Adapter | Supplies power to the Arduino | E.g., Digikey: 364-1268-ND | $7.96 |
|  | Breadboard | Hold circuits components and wires | E.g., Digikey: 2183-4000-ND | $2.55 |
|  | Jumper Wires | Connects components | E.g., Digikey: BKWK-3-ND | $3.90 |
