# Supplementary figures and images for: BrainWAVE: A Flexible Method for Noninvasive Stimulation of Brain Rhythms across Species (part 2 of 2)
Source: eNeuro. 2023 Feb 23;10(2):ENEURO.0257-22.2022. doi: 10.1523/ENEURO.0257-22.2022 (PMC9979148; doi:10.1523/ENEURO.0257-22.2022)

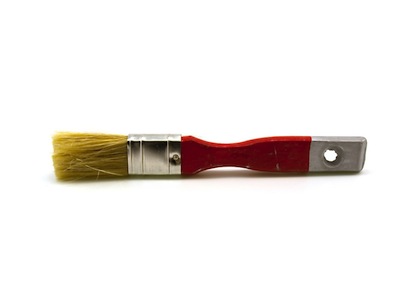

Supplement: Extended Data 1 — BrainWAVE stimulator code. These files contain code to generate and play flicker sensory stimulation with an Arduino Uno or NIDAQ BrainWAVE stimulator device. Download Extended Data 1, ZIP file. [file enu-eN-OTM-0257-22-s05.zip › Code_FliCkER/Code_FliCkER/GUI_FliCkER/functions_and_parameters/FlickerMemoryTask/imageset_1a/Set1_107a.jpg]

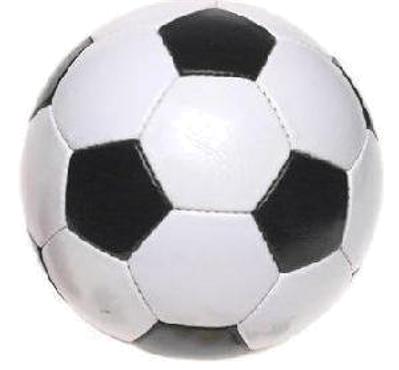

Supplement: Extended Data 1 — BrainWAVE stimulator code. These files contain code to generate and play flicker sensory stimulation with an Arduino Uno or NIDAQ BrainWAVE stimulator device. Download Extended Data 1, ZIP file. [file enu-eN-OTM-0257-22-s05.zip › Code_FliCkER/Code_FliCkER/GUI_FliCkER/functions_and_parameters/FlickerMemoryTask/imageset_1a/Set1_108a.jpg]

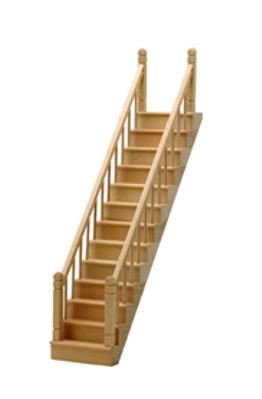

Supplement: Extended Data 1 — BrainWAVE stimulator code. These files contain code to generate and play flicker sensory stimulation with an Arduino Uno or NIDAQ BrainWAVE stimulator device. Download Extended Data 1, ZIP file. [file enu-eN-OTM-0257-22-s05.zip › Code_FliCkER/Code_FliCkER/GUI_FliCkER/functions_and_parameters/FlickerMemoryTask/imageset_1a/Set1_109a.jpg]

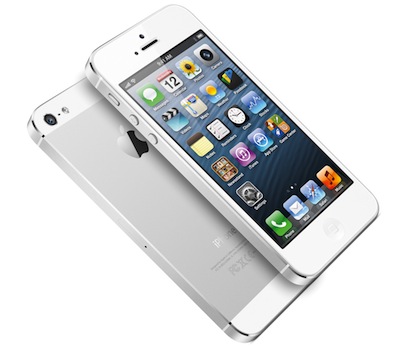

Supplement: Extended Data 1 — BrainWAVE stimulator code. These files contain code to generate and play flicker sensory stimulation with an Arduino Uno or NIDAQ BrainWAVE stimulator device. Download Extended Data 1, ZIP file. [file enu-eN-OTM-0257-22-s05.zip › Code_FliCkER/Code_FliCkER/GUI_FliCkER/functions_and_parameters/FlickerMemoryTask/imageset_1a/Set1_110a.jpg]

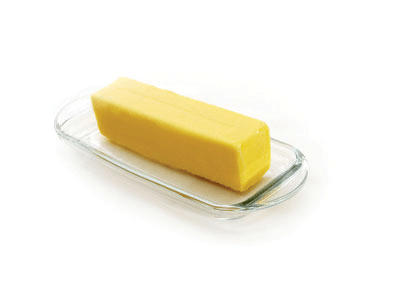

Supplement: Extended Data 1 — BrainWAVE stimulator code. These files contain code to generate and play flicker sensory stimulation with an Arduino Uno or NIDAQ BrainWAVE stimulator device. Download Extended Data 1, ZIP file. [file enu-eN-OTM-0257-22-s05.zip › Code_FliCkER/Code_FliCkER/GUI_FliCkER/functions_and_parameters/FlickerMemoryTask/imageset_1a/Set1_111a.jpg]

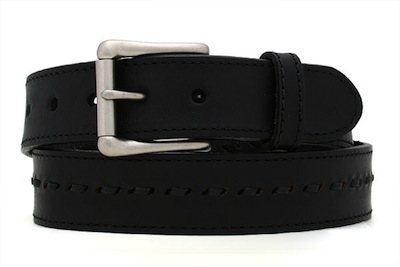

Supplement: Extended Data 1 — BrainWAVE stimulator code. These files contain code to generate and play flicker sensory stimulation with an Arduino Uno or NIDAQ BrainWAVE stimulator device. Download Extended Data 1, ZIP file. [file enu-eN-OTM-0257-22-s05.zip › Code_FliCkER/Code_FliCkER/GUI_FliCkER/functions_and_parameters/FlickerMemoryTask/imageset_1a/Set1_112a.jpg]

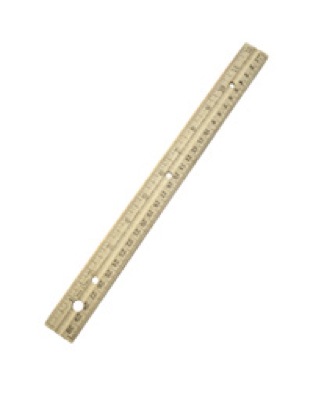

Supplement: Extended Data 1 — BrainWAVE stimulator code. These files contain code to generate and play flicker sensory stimulation with an Arduino Uno or NIDAQ BrainWAVE stimulator device. Download Extended Data 1, ZIP file. [file enu-eN-OTM-0257-22-s05.zip › Code_FliCkER/Code_FliCkER/GUI_FliCkER/functions_and_parameters/FlickerMemoryTask/imageset_1a/Set1_113a.jpg]

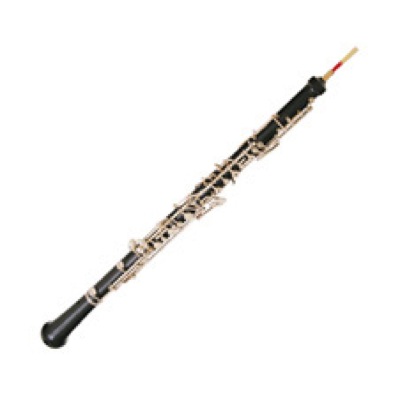

Supplement: Extended Data 1 — BrainWAVE stimulator code. These files contain code to generate and play flicker sensory stimulation with an Arduino Uno or NIDAQ BrainWAVE stimulator device. Download Extended Data 1, ZIP file. [file enu-eN-OTM-0257-22-s05.zip › Code_FliCkER/Code_FliCkER/GUI_FliCkER/functions_and_parameters/FlickerMemoryTask/imageset_1a/Set1_114a.jpg]

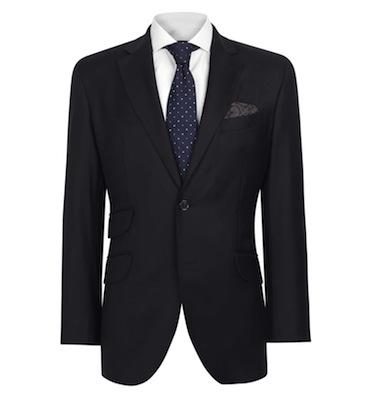

Supplement: Extended Data 1 — BrainWAVE stimulator code. These files contain code to generate and play flicker sensory stimulation with an Arduino Uno or NIDAQ BrainWAVE stimulator device. Download Extended Data 1, ZIP file. [file enu-eN-OTM-0257-22-s05.zip › Code_FliCkER/Code_FliCkER/GUI_FliCkER/functions_and_parameters/FlickerMemoryTask/imageset_1a/Set1_116a.jpg]

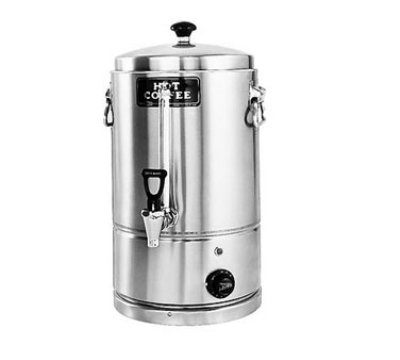

Supplement: Extended Data 1 — BrainWAVE stimulator code. These files contain code to generate and play flicker sensory stimulation with an Arduino Uno or NIDAQ BrainWAVE stimulator device. Download Extended Data 1, ZIP file. [file enu-eN-OTM-0257-22-s05.zip › Code_FliCkER/Code_FliCkER/GUI_FliCkER/functions_and_parameters/FlickerMemoryTask/imageset_1a/Set1_117a.jpg]

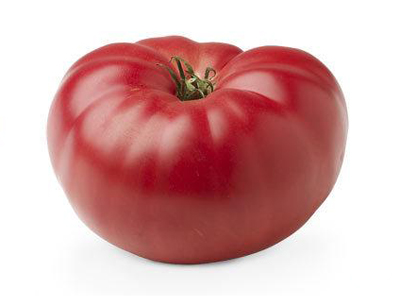

Supplement: Extended Data 1 — BrainWAVE stimulator code. These files contain code to generate and play flicker sensory stimulation with an Arduino Uno or NIDAQ BrainWAVE stimulator device. Download Extended Data 1, ZIP file. [file enu-eN-OTM-0257-22-s05.zip › Code_FliCkER/Code_FliCkER/GUI_FliCkER/functions_and_parameters/FlickerMemoryTask/imageset_1a/Set1_118a.jpg]

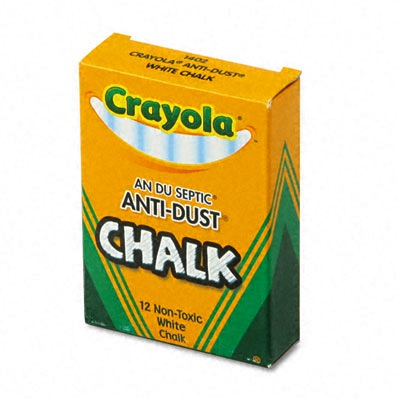

Supplement: Extended Data 1 — BrainWAVE stimulator code. These files contain code to generate and play flicker sensory stimulation with an Arduino Uno or NIDAQ BrainWAVE stimulator device. Download Extended Data 1, ZIP file. [file enu-eN-OTM-0257-22-s05.zip › Code_FliCkER/Code_FliCkER/GUI_FliCkER/functions_and_parameters/FlickerMemoryTask/imageset_1a/Set1_119a.jpg]

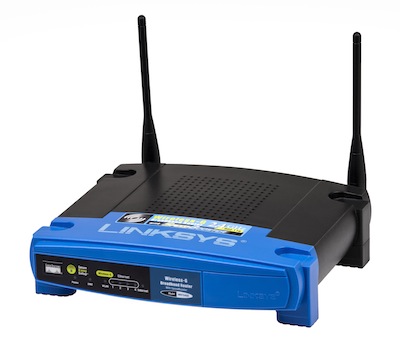

Supplement: Extended Data 1 — BrainWAVE stimulator code. These files contain code to generate and play flicker sensory stimulation with an Arduino Uno or NIDAQ BrainWAVE stimulator device. Download Extended Data 1, ZIP file. [file enu-eN-OTM-0257-22-s05.zip › Code_FliCkER/Code_FliCkER/GUI_FliCkER/functions_and_parameters/FlickerMemoryTask/imageset_1a/Set1_120a.jpg]

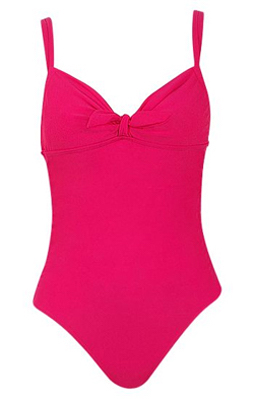

Supplement: Extended Data 1 — BrainWAVE stimulator code. These files contain code to generate and play flicker sensory stimulation with an Arduino Uno or NIDAQ BrainWAVE stimulator device. Download Extended Data 1, ZIP file. [file enu-eN-OTM-0257-22-s05.zip › Code_FliCkER/Code_FliCkER/GUI_FliCkER/functions_and_parameters/FlickerMemoryTask/imageset_1a/Set1_121a.jpg]

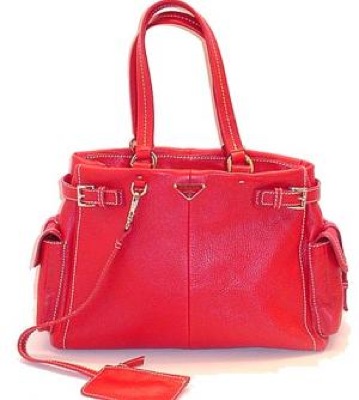

Supplement: Extended Data 1 — BrainWAVE stimulator code. These files contain code to generate and play flicker sensory stimulation with an Arduino Uno or NIDAQ BrainWAVE stimulator device. Download Extended Data 1, ZIP file. [file enu-eN-OTM-0257-22-s05.zip › Code_FliCkER/Code_FliCkER/GUI_FliCkER/functions_and_parameters/FlickerMemoryTask/imageset_1a/Set1_122a.jpg]

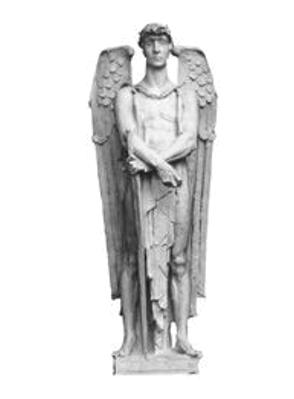

Supplement: Extended Data 1 — BrainWAVE stimulator code. These files contain code to generate and play flicker sensory stimulation with an Arduino Uno or NIDAQ BrainWAVE stimulator device. Download Extended Data 1, ZIP file. [file enu-eN-OTM-0257-22-s05.zip › Code_FliCkER/Code_FliCkER/GUI_FliCkER/functions_and_parameters/FlickerMemoryTask/imageset_1a/Set1_123a.jpg]

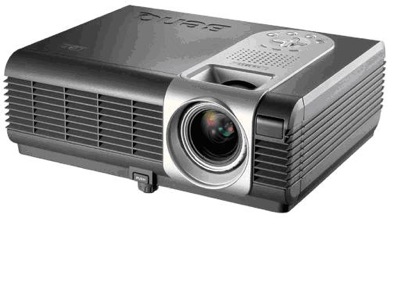

Supplement: Extended Data 1 — BrainWAVE stimulator code. These files contain code to generate and play flicker sensory stimulation with an Arduino Uno or NIDAQ BrainWAVE stimulator device. Download Extended Data 1, ZIP file. [file enu-eN-OTM-0257-22-s05.zip › Code_FliCkER/Code_FliCkER/GUI_FliCkER/functions_and_parameters/FlickerMemoryTask/imageset_1a/Set1_124a.jpg]

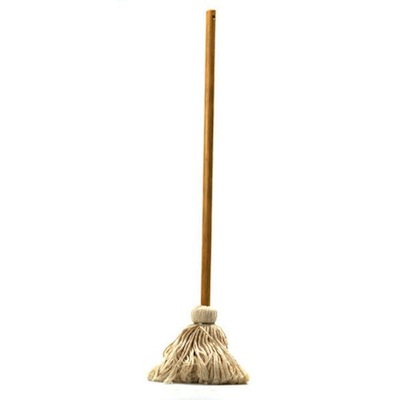

Supplement: Extended Data 1 — BrainWAVE stimulator code. These files contain code to generate and play flicker sensory stimulation with an Arduino Uno or NIDAQ BrainWAVE stimulator device. Download Extended Data 1, ZIP file. [file enu-eN-OTM-0257-22-s05.zip › Code_FliCkER/Code_FliCkER/GUI_FliCkER/functions_and_parameters/FlickerMemoryTask/imageset_1a/Set1_125a.jpg]

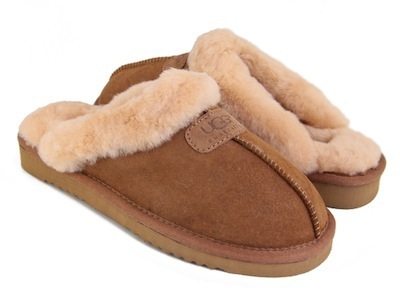

Supplement: Extended Data 1 — BrainWAVE stimulator code. These files contain code to generate and play flicker sensory stimulation with an Arduino Uno or NIDAQ BrainWAVE stimulator device. Download Extended Data 1, ZIP file. [file enu-eN-OTM-0257-22-s05.zip › Code_FliCkER/Code_FliCkER/GUI_FliCkER/functions_and_parameters/FlickerMemoryTask/imageset_1a/Set1_126a.jpg]

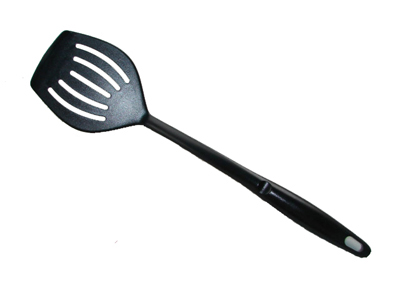

Supplement: Extended Data 1 — BrainWAVE stimulator code. These files contain code to generate and play flicker sensory stimulation with an Arduino Uno or NIDAQ BrainWAVE stimulator device. Download Extended Data 1, ZIP file. [file enu-eN-OTM-0257-22-s05.zip › Code_FliCkER/Code_FliCkER/GUI_FliCkER/functions_and_parameters/FlickerMemoryTask/imageset_1a/Set1_127a.jpg]

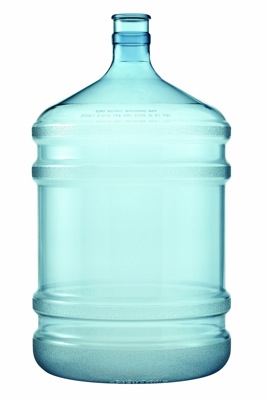

Supplement: Extended Data 1 — BrainWAVE stimulator code. These files contain code to generate and play flicker sensory stimulation with an Arduino Uno or NIDAQ BrainWAVE stimulator device. Download Extended Data 1, ZIP file. [file enu-eN-OTM-0257-22-s05.zip › Code_FliCkER/Code_FliCkER/GUI_FliCkER/functions_and_parameters/FlickerMemoryTask/imageset_1a/Set1_128a.jpg]

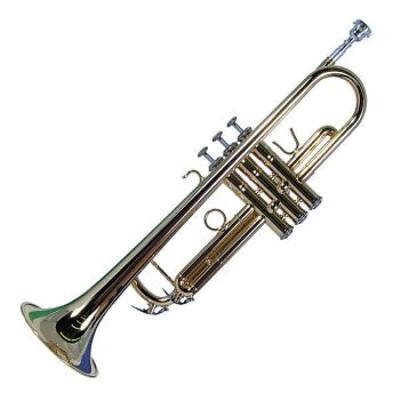

Supplement: Extended Data 1 — BrainWAVE stimulator code. These files contain code to generate and play flicker sensory stimulation with an Arduino Uno or NIDAQ BrainWAVE stimulator device. Download Extended Data 1, ZIP file. [file enu-eN-OTM-0257-22-s05.zip › Code_FliCkER/Code_FliCkER/GUI_FliCkER/functions_and_parameters/FlickerMemoryTask/imageset_1a/Set1_129a.jpg]

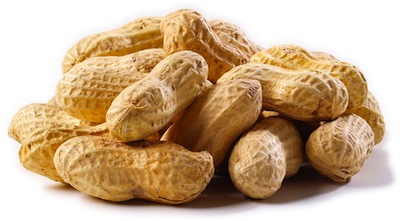

Supplement: Extended Data 1 — BrainWAVE stimulator code. These files contain code to generate and play flicker sensory stimulation with an Arduino Uno or NIDAQ BrainWAVE stimulator device. Download Extended Data 1, ZIP file. [file enu-eN-OTM-0257-22-s05.zip › Code_FliCkER/Code_FliCkER/GUI_FliCkER/functions_and_parameters/FlickerMemoryTask/imageset_1a/Set1_130a.jpg]

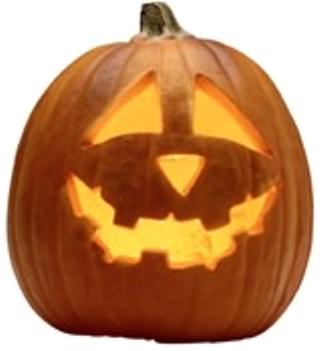

Supplement: Extended Data 1 — BrainWAVE stimulator code. These files contain code to generate and play flicker sensory stimulation with an Arduino Uno or NIDAQ BrainWAVE stimulator device. Download Extended Data 1, ZIP file. [file enu-eN-OTM-0257-22-s05.zip › Code_FliCkER/Code_FliCkER/GUI_FliCkER/functions_and_parameters/FlickerMemoryTask/imageset_1a/Set1_131a.jpg]

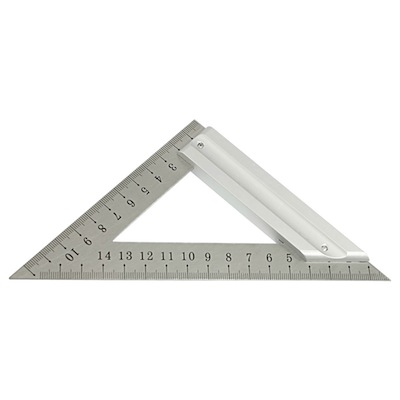

Supplement: Extended Data 1 — BrainWAVE stimulator code. These files contain code to generate and play flicker sensory stimulation with an Arduino Uno or NIDAQ BrainWAVE stimulator device. Download Extended Data 1, ZIP file. [file enu-eN-OTM-0257-22-s05.zip › Code_FliCkER/Code_FliCkER/GUI_FliCkER/functions_and_parameters/FlickerMemoryTask/imageset_1a/Set1_132a.jpg]

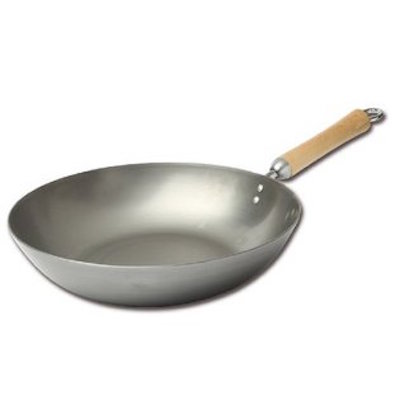

Supplement: Extended Data 1 — BrainWAVE stimulator code. These files contain code to generate and play flicker sensory stimulation with an Arduino Uno or NIDAQ BrainWAVE stimulator device. Download Extended Data 1, ZIP file. [file enu-eN-OTM-0257-22-s05.zip › Code_FliCkER/Code_FliCkER/GUI_FliCkER/functions_and_parameters/FlickerMemoryTask/imageset_1a/Set1_133a.jpg]

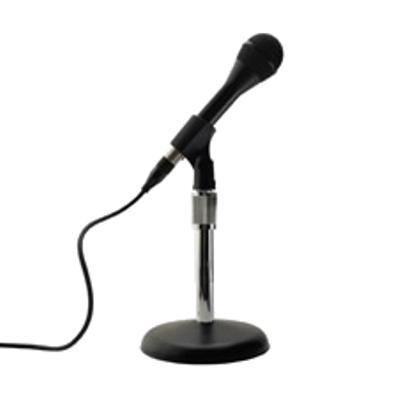

Supplement: Extended Data 1 — BrainWAVE stimulator code. These files contain code to generate and play flicker sensory stimulation with an Arduino Uno or NIDAQ BrainWAVE stimulator device. Download Extended Data 1, ZIP file. [file enu-eN-OTM-0257-22-s05.zip › Code_FliCkER/Code_FliCkER/GUI_FliCkER/functions_and_parameters/FlickerMemoryTask/imageset_1a/Set1_134a.jpg]

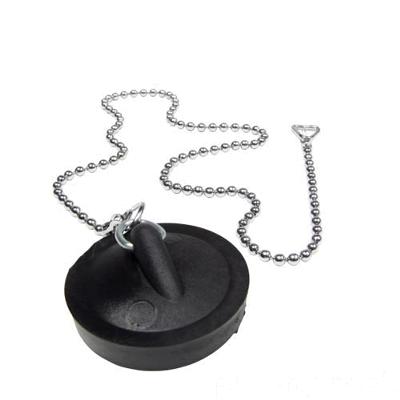

Supplement: Extended Data 1 — BrainWAVE stimulator code. These files contain code to generate and play flicker sensory stimulation with an Arduino Uno or NIDAQ BrainWAVE stimulator device. Download Extended Data 1, ZIP file. [file enu-eN-OTM-0257-22-s05.zip › Code_FliCkER/Code_FliCkER/GUI_FliCkER/functions_and_parameters/FlickerMemoryTask/imageset_1a/Set1_135a.jpg]

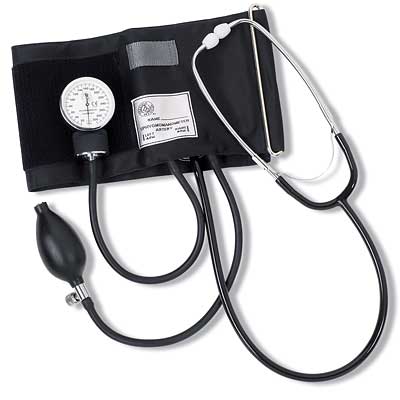

Supplement: Extended Data 1 — BrainWAVE stimulator code. These files contain code to generate and play flicker sensory stimulation with an Arduino Uno or NIDAQ BrainWAVE stimulator device. Download Extended Data 1, ZIP file. [file enu-eN-OTM-0257-22-s05.zip › Code_FliCkER/Code_FliCkER/GUI_FliCkER/functions_and_parameters/FlickerMemoryTask/imageset_1a/Set1_136a.jpg]

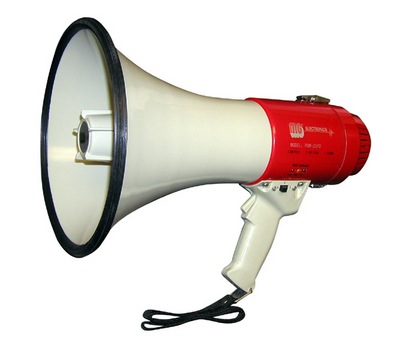

Supplement: Extended Data 1 — BrainWAVE stimulator code. These files contain code to generate and play flicker sensory stimulation with an Arduino Uno or NIDAQ BrainWAVE stimulator device. Download Extended Data 1, ZIP file. [file enu-eN-OTM-0257-22-s05.zip › Code_FliCkER/Code_FliCkER/GUI_FliCkER/functions_and_parameters/FlickerMemoryTask/imageset_1a/Set1_137a.jpg]

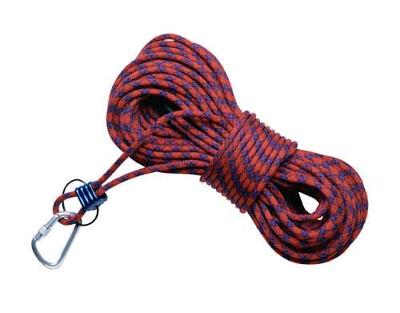

Supplement: Extended Data 1 — BrainWAVE stimulator code. These files contain code to generate and play flicker sensory stimulation with an Arduino Uno or NIDAQ BrainWAVE stimulator device. Download Extended Data 1, ZIP file. [file enu-eN-OTM-0257-22-s05.zip › Code_FliCkER/Code_FliCkER/GUI_FliCkER/functions_and_parameters/FlickerMemoryTask/imageset_1a/Set1_138a.jpg]

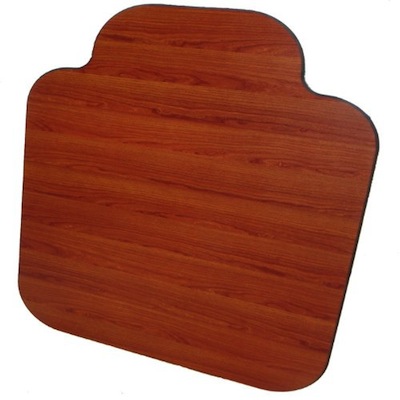

Supplement: Extended Data 1 — BrainWAVE stimulator code. These files contain code to generate and play flicker sensory stimulation with an Arduino Uno or NIDAQ BrainWAVE stimulator device. Download Extended Data 1, ZIP file. [file enu-eN-OTM-0257-22-s05.zip › Code_FliCkER/Code_FliCkER/GUI_FliCkER/functions_and_parameters/FlickerMemoryTask/imageset_1a/Set1_139a.jpg]

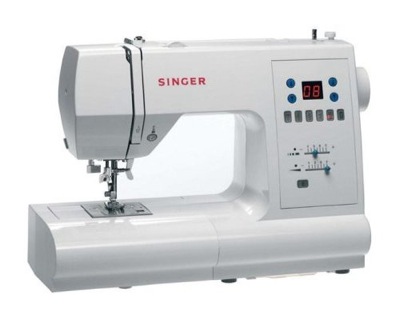

Supplement: Extended Data 1 — BrainWAVE stimulator code. These files contain code to generate and play flicker sensory stimulation with an Arduino Uno or NIDAQ BrainWAVE stimulator device. Download Extended Data 1, ZIP file. [file enu-eN-OTM-0257-22-s05.zip › Code_FliCkER/Code_FliCkER/GUI_FliCkER/functions_and_parameters/FlickerMemoryTask/imageset_1a/Set1_140a.jpg]

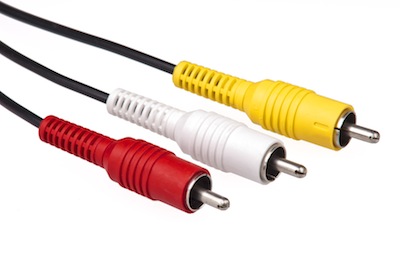

Supplement: Extended Data 1 — BrainWAVE stimulator code. These files contain code to generate and play flicker sensory stimulation with an Arduino Uno or NIDAQ BrainWAVE stimulator device. Download Extended Data 1, ZIP file. [file enu-eN-OTM-0257-22-s05.zip › Code_FliCkER/Code_FliCkER/GUI_FliCkER/functions_and_parameters/FlickerMemoryTask/imageset_1a/Set1_141a.jpg]

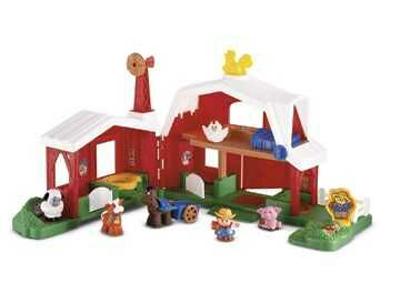

Supplement: Extended Data 1 — BrainWAVE stimulator code. These files contain code to generate and play flicker sensory stimulation with an Arduino Uno or NIDAQ BrainWAVE stimulator device. Download Extended Data 1, ZIP file. [file enu-eN-OTM-0257-22-s05.zip › Code_FliCkER/Code_FliCkER/GUI_FliCkER/functions_and_parameters/FlickerMemoryTask/imageset_1a/Set1_143a.jpg]

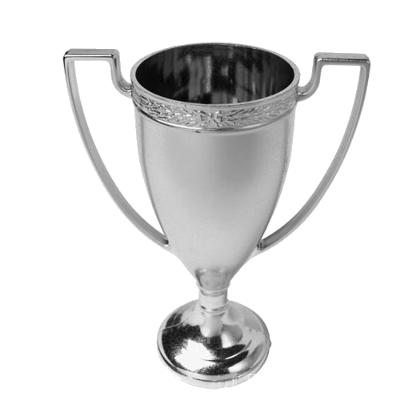

Supplement: Extended Data 1 — BrainWAVE stimulator code. These files contain code to generate and play flicker sensory stimulation with an Arduino Uno or NIDAQ BrainWAVE stimulator device. Download Extended Data 1, ZIP file. [file enu-eN-OTM-0257-22-s05.zip › Code_FliCkER/Code_FliCkER/GUI_FliCkER/functions_and_parameters/FlickerMemoryTask/imageset_1a/Set1_145a.jpg]

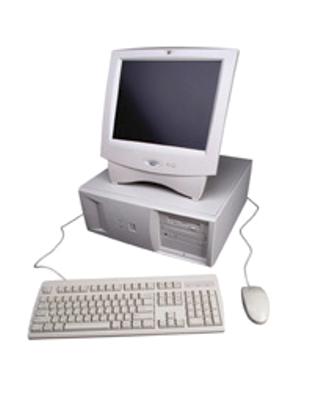

Supplement: Extended Data 1 — BrainWAVE stimulator code. These files contain code to generate and play flicker sensory stimulation with an Arduino Uno or NIDAQ BrainWAVE stimulator device. Download Extended Data 1, ZIP file. [file enu-eN-OTM-0257-22-s05.zip › Code_FliCkER/Code_FliCkER/GUI_FliCkER/functions_and_parameters/FlickerMemoryTask/imageset_1a/Set1_146a.jpg]

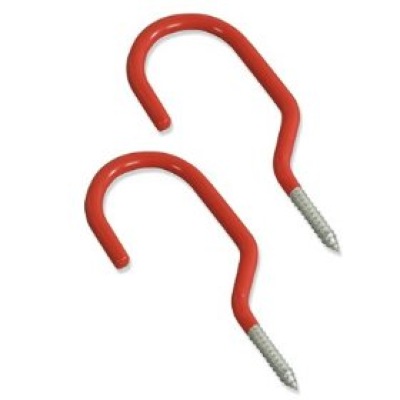

Supplement: Extended Data 1 — BrainWAVE stimulator code. These files contain code to generate and play flicker sensory stimulation with an Arduino Uno or NIDAQ BrainWAVE stimulator device. Download Extended Data 1, ZIP file. [file enu-eN-OTM-0257-22-s05.zip › Code_FliCkER/Code_FliCkER/GUI_FliCkER/functions_and_parameters/FlickerMemoryTask/imageset_1a/Set1_147a.jpg]

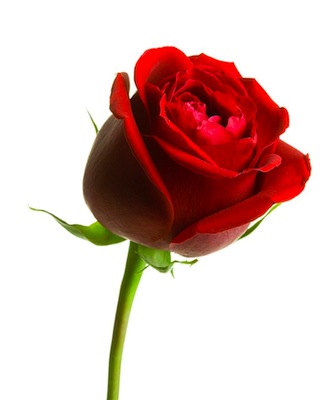

Supplement: Extended Data 1 — BrainWAVE stimulator code. These files contain code to generate and play flicker sensory stimulation with an Arduino Uno or NIDAQ BrainWAVE stimulator device. Download Extended Data 1, ZIP file. [file enu-eN-OTM-0257-22-s05.zip › Code_FliCkER/Code_FliCkER/GUI_FliCkER/functions_and_parameters/FlickerMemoryTask/imageset_1a/Set1_148a.jpg]

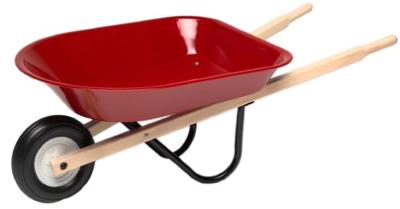

Supplement: Extended Data 1 — BrainWAVE stimulator code. These files contain code to generate and play flicker sensory stimulation with an Arduino Uno or NIDAQ BrainWAVE stimulator device. Download Extended Data 1, ZIP file. [file enu-eN-OTM-0257-22-s05.zip › Code_FliCkER/Code_FliCkER/GUI_FliCkER/functions_and_parameters/FlickerMemoryTask/imageset_1a/Set1_149a.jpg]

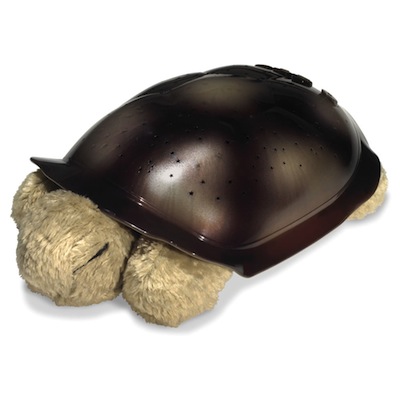

Supplement: Extended Data 1 — BrainWAVE stimulator code. These files contain code to generate and play flicker sensory stimulation with an Arduino Uno or NIDAQ BrainWAVE stimulator device. Download Extended Data 1, ZIP file. [file enu-eN-OTM-0257-22-s05.zip › Code_FliCkER/Code_FliCkER/GUI_FliCkER/functions_and_parameters/FlickerMemoryTask/imageset_1a/Set1_150a.jpg]

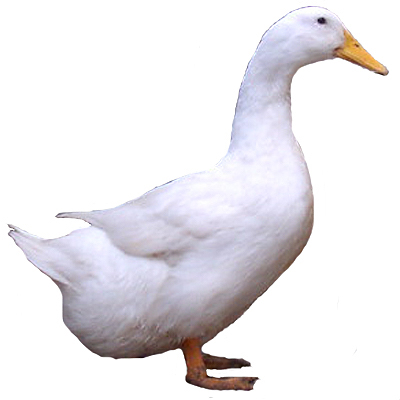

Supplement: Extended Data 1 — BrainWAVE stimulator code. These files contain code to generate and play flicker sensory stimulation with an Arduino Uno or NIDAQ BrainWAVE stimulator device. Download Extended Data 1, ZIP file. [file enu-eN-OTM-0257-22-s05.zip › Code_FliCkER/Code_FliCkER/GUI_FliCkER/functions_and_parameters/FlickerMemoryTask/imageset_1a/Set1_152a.jpg]

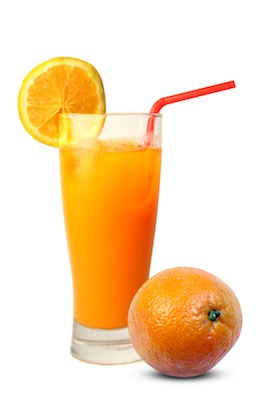

Supplement: Extended Data 1 — BrainWAVE stimulator code. These files contain code to generate and play flicker sensory stimulation with an Arduino Uno or NIDAQ BrainWAVE stimulator device. Download Extended Data 1, ZIP file. [file enu-eN-OTM-0257-22-s05.zip › Code_FliCkER/Code_FliCkER/GUI_FliCkER/functions_and_parameters/FlickerMemoryTask/imageset_1a/Set1_153a.jpg]

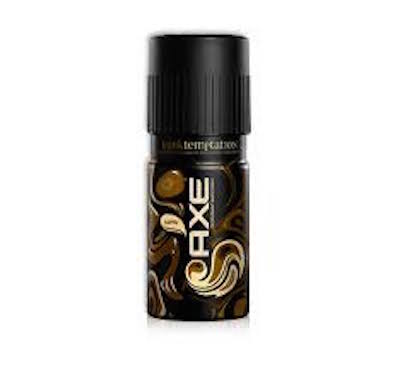

Supplement: Extended Data 1 — BrainWAVE stimulator code. These files contain code to generate and play flicker sensory stimulation with an Arduino Uno or NIDAQ BrainWAVE stimulator device. Download Extended Data 1, ZIP file. [file enu-eN-OTM-0257-22-s05.zip › Code_FliCkER/Code_FliCkER/GUI_FliCkER/functions_and_parameters/FlickerMemoryTask/imageset_1a/Set1_154a.jpg]

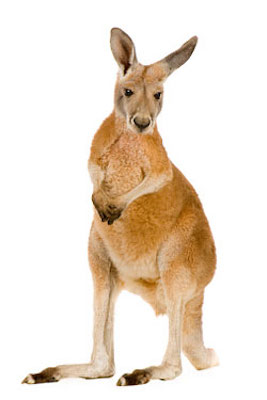

Supplement: Extended Data 1 — BrainWAVE stimulator code. These files contain code to generate and play flicker sensory stimulation with an Arduino Uno or NIDAQ BrainWAVE stimulator device. Download Extended Data 1, ZIP file. [file enu-eN-OTM-0257-22-s05.zip › Code_FliCkER/Code_FliCkER/GUI_FliCkER/functions_and_parameters/FlickerMemoryTask/imageset_1a/Set1_155a.jpg]

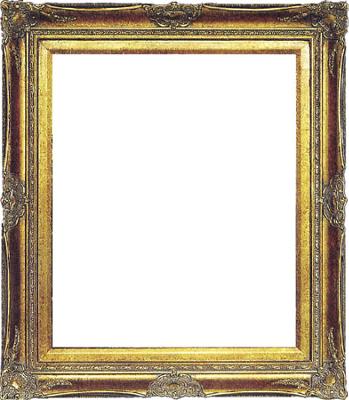

Supplement: Extended Data 1 — BrainWAVE stimulator code. These files contain code to generate and play flicker sensory stimulation with an Arduino Uno or NIDAQ BrainWAVE stimulator device. Download Extended Data 1, ZIP file. [file enu-eN-OTM-0257-22-s05.zip › Code_FliCkER/Code_FliCkER/GUI_FliCkER/functions_and_parameters/FlickerMemoryTask/imageset_1a/Set1_156a.jpg]

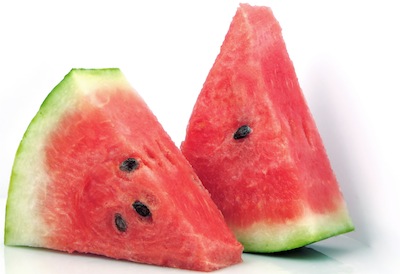

Supplement: Extended Data 1 — BrainWAVE stimulator code. These files contain code to generate and play flicker sensory stimulation with an Arduino Uno or NIDAQ BrainWAVE stimulator device. Download Extended Data 1, ZIP file. [file enu-eN-OTM-0257-22-s05.zip › Code_FliCkER/Code_FliCkER/GUI_FliCkER/functions_and_parameters/FlickerMemoryTask/imageset_1a/Set1_157a.jpg]

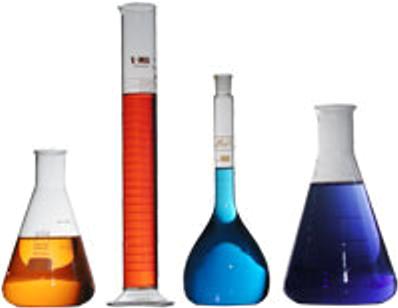

Supplement: Extended Data 1 — BrainWAVE stimulator code. These files contain code to generate and play flicker sensory stimulation with an Arduino Uno or NIDAQ BrainWAVE stimulator device. Download Extended Data 1, ZIP file. [file enu-eN-OTM-0257-22-s05.zip › Code_FliCkER/Code_FliCkER/GUI_FliCkER/functions_and_parameters/FlickerMemoryTask/imageset_1a/Set1_158a.jpg]

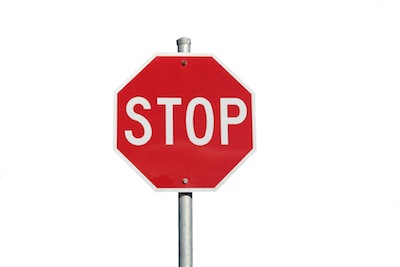

Supplement: Extended Data 1 — BrainWAVE stimulator code. These files contain code to generate and play flicker sensory stimulation with an Arduino Uno or NIDAQ BrainWAVE stimulator device. Download Extended Data 1, ZIP file. [file enu-eN-OTM-0257-22-s05.zip › Code_FliCkER/Code_FliCkER/GUI_FliCkER/functions_and_parameters/FlickerMemoryTask/imageset_1a/Set1_159a.jpg]

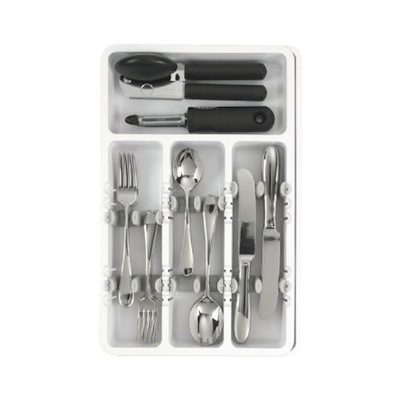

Supplement: Extended Data 1 — BrainWAVE stimulator code. These files contain code to generate and play flicker sensory stimulation with an Arduino Uno or NIDAQ BrainWAVE stimulator device. Download Extended Data 1, ZIP file. [file enu-eN-OTM-0257-22-s05.zip › Code_FliCkER/Code_FliCkER/GUI_FliCkER/functions_and_parameters/FlickerMemoryTask/imageset_1a/Set1_160a.jpg]

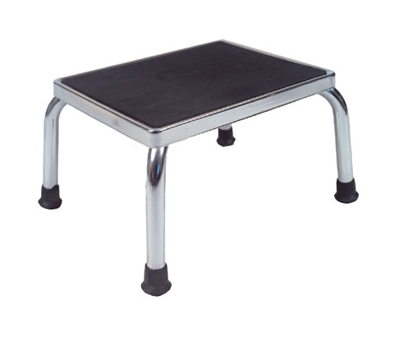

Supplement: Extended Data 1 — BrainWAVE stimulator code. These files contain code to generate and play flicker sensory stimulation with an Arduino Uno or NIDAQ BrainWAVE stimulator device. Download Extended Data 1, ZIP file. [file enu-eN-OTM-0257-22-s05.zip › Code_FliCkER/Code_FliCkER/GUI_FliCkER/functions_and_parameters/FlickerMemoryTask/imageset_1a/Set1_161a.jpg]

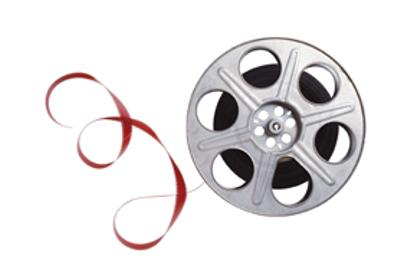

Supplement: Extended Data 1 — BrainWAVE stimulator code. These files contain code to generate and play flicker sensory stimulation with an Arduino Uno or NIDAQ BrainWAVE stimulator device. Download Extended Data 1, ZIP file. [file enu-eN-OTM-0257-22-s05.zip › Code_FliCkER/Code_FliCkER/GUI_FliCkER/functions_and_parameters/FlickerMemoryTask/imageset_1a/Set1_162a.jpg]

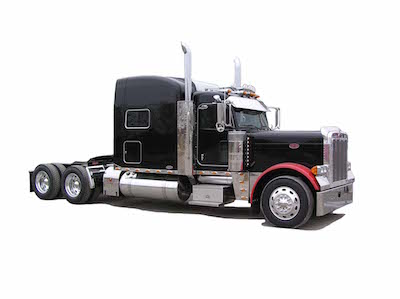

Supplement: Extended Data 1 — BrainWAVE stimulator code. These files contain code to generate and play flicker sensory stimulation with an Arduino Uno or NIDAQ BrainWAVE stimulator device. Download Extended Data 1, ZIP file. [file enu-eN-OTM-0257-22-s05.zip › Code_FliCkER/Code_FliCkER/GUI_FliCkER/functions_and_parameters/FlickerMemoryTask/imageset_1a/Set1_163a.jpg]

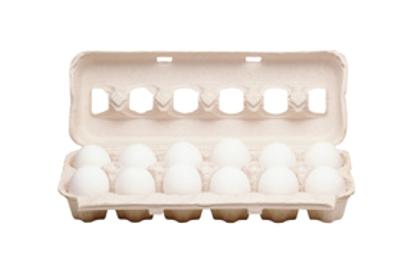

Supplement: Extended Data 1 — BrainWAVE stimulator code. These files contain code to generate and play flicker sensory stimulation with an Arduino Uno or NIDAQ BrainWAVE stimulator device. Download Extended Data 1, ZIP file. [file enu-eN-OTM-0257-22-s05.zip › Code_FliCkER/Code_FliCkER/GUI_FliCkER/functions_and_parameters/FlickerMemoryTask/imageset_1a/Set1_164a.jpg]

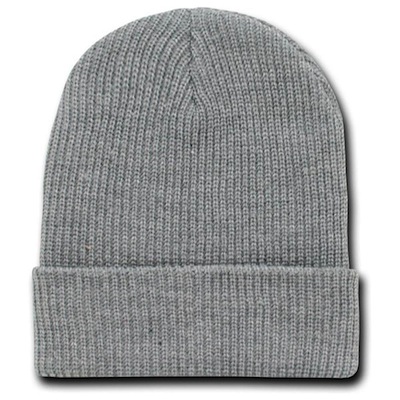

Supplement: Extended Data 1 — BrainWAVE stimulator code. These files contain code to generate and play flicker sensory stimulation with an Arduino Uno or NIDAQ BrainWAVE stimulator device. Download Extended Data 1, ZIP file. [file enu-eN-OTM-0257-22-s05.zip › Code_FliCkER/Code_FliCkER/GUI_FliCkER/functions_and_parameters/FlickerMemoryTask/imageset_1a/Set1_165a.jpg]

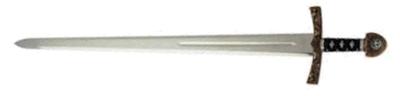

Supplement: Extended Data 1 — BrainWAVE stimulator code. These files contain code to generate and play flicker sensory stimulation with an Arduino Uno or NIDAQ BrainWAVE stimulator device. Download Extended Data 1, ZIP file. [file enu-eN-OTM-0257-22-s05.zip › Code_FliCkER/Code_FliCkER/GUI_FliCkER/functions_and_parameters/FlickerMemoryTask/imageset_1a/Set1_166a.jpg]

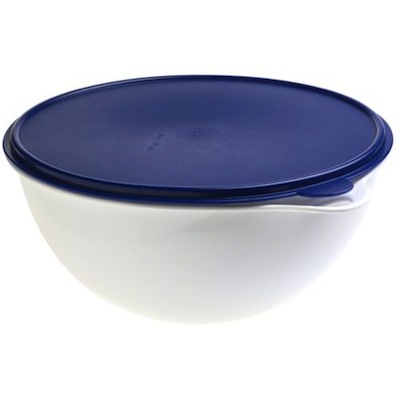

Supplement: Extended Data 1 — BrainWAVE stimulator code. These files contain code to generate and play flicker sensory stimulation with an Arduino Uno or NIDAQ BrainWAVE stimulator device. Download Extended Data 1, ZIP file. [file enu-eN-OTM-0257-22-s05.zip › Code_FliCkER/Code_FliCkER/GUI_FliCkER/functions_and_parameters/FlickerMemoryTask/imageset_1a/Set1_167a.jpg]

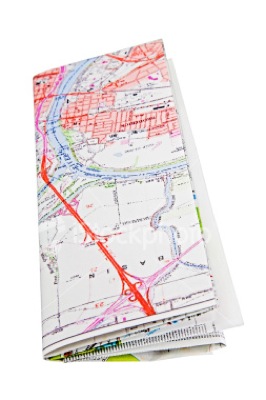

Supplement: Extended Data 1 — BrainWAVE stimulator code. These files contain code to generate and play flicker sensory stimulation with an Arduino Uno or NIDAQ BrainWAVE stimulator device. Download Extended Data 1, ZIP file. [file enu-eN-OTM-0257-22-s05.zip › Code_FliCkER/Code_FliCkER/GUI_FliCkER/functions_and_parameters/FlickerMemoryTask/imageset_1a/Set1_168a.jpg]

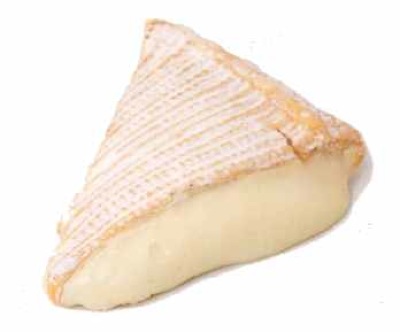

Supplement: Extended Data 1 — BrainWAVE stimulator code. These files contain code to generate and play flicker sensory stimulation with an Arduino Uno or NIDAQ BrainWAVE stimulator device. Download Extended Data 1, ZIP file. [file enu-eN-OTM-0257-22-s05.zip › Code_FliCkER/Code_FliCkER/GUI_FliCkER/functions_and_parameters/FlickerMemoryTask/imageset_1a/Set1_170a.jpg]

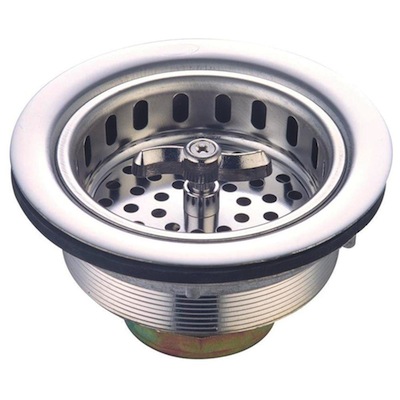

Supplement: Extended Data 1 — BrainWAVE stimulator code. These files contain code to generate and play flicker sensory stimulation with an Arduino Uno or NIDAQ BrainWAVE stimulator device. Download Extended Data 1, ZIP file. [file enu-eN-OTM-0257-22-s05.zip › Code_FliCkER/Code_FliCkER/GUI_FliCkER/functions_and_parameters/FlickerMemoryTask/imageset_1a/Set1_171a.jpg]

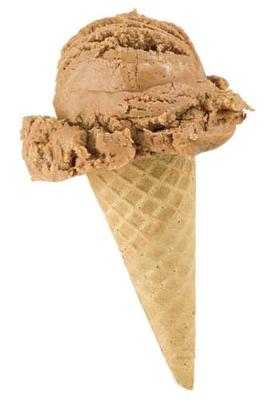

Supplement: Extended Data 1 — BrainWAVE stimulator code. These files contain code to generate and play flicker sensory stimulation with an Arduino Uno or NIDAQ BrainWAVE stimulator device. Download Extended Data 1, ZIP file. [file enu-eN-OTM-0257-22-s05.zip › Code_FliCkER/Code_FliCkER/GUI_FliCkER/functions_and_parameters/FlickerMemoryTask/imageset_1a/Set1_172a.jpg]

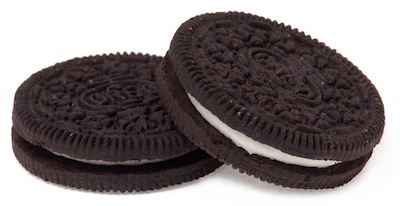

Supplement: Extended Data 1 — BrainWAVE stimulator code. These files contain code to generate and play flicker sensory stimulation with an Arduino Uno or NIDAQ BrainWAVE stimulator device. Download Extended Data 1, ZIP file. [file enu-eN-OTM-0257-22-s05.zip › Code_FliCkER/Code_FliCkER/GUI_FliCkER/functions_and_parameters/FlickerMemoryTask/imageset_1a/Set1_173a.jpg]

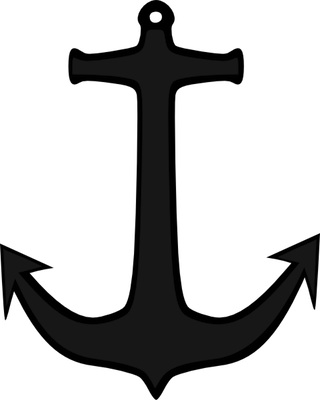

Supplement: Extended Data 1 — BrainWAVE stimulator code. These files contain code to generate and play flicker sensory stimulation with an Arduino Uno or NIDAQ BrainWAVE stimulator device. Download Extended Data 1, ZIP file. [file enu-eN-OTM-0257-22-s05.zip › Code_FliCkER/Code_FliCkER/GUI_FliCkER/functions_and_parameters/FlickerMemoryTask/imageset_1a/Set1_174a.jpg]

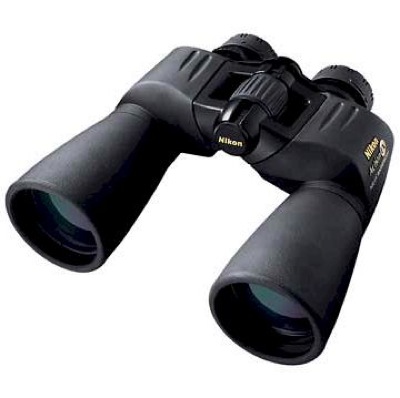

Supplement: Extended Data 1 — BrainWAVE stimulator code. These files contain code to generate and play flicker sensory stimulation with an Arduino Uno or NIDAQ BrainWAVE stimulator device. Download Extended Data 1, ZIP file. [file enu-eN-OTM-0257-22-s05.zip › Code_FliCkER/Code_FliCkER/GUI_FliCkER/functions_and_parameters/FlickerMemoryTask/imageset_1a/Set1_175a.jpg]

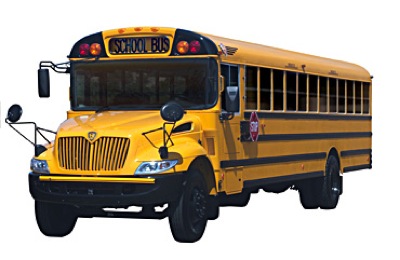

Supplement: Extended Data 1 — BrainWAVE stimulator code. These files contain code to generate and play flicker sensory stimulation with an Arduino Uno or NIDAQ BrainWAVE stimulator device. Download Extended Data 1, ZIP file. [file enu-eN-OTM-0257-22-s05.zip › Code_FliCkER/Code_FliCkER/GUI_FliCkER/functions_and_parameters/FlickerMemoryTask/imageset_1a/Set1_176a.jpg]

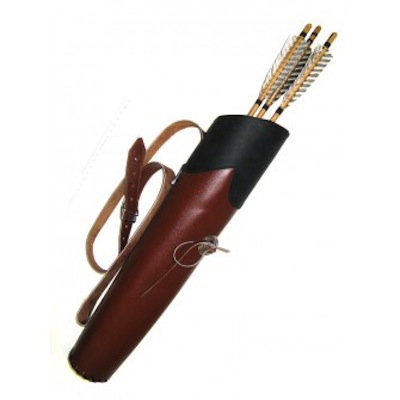

Supplement: Extended Data 1 — BrainWAVE stimulator code. These files contain code to generate and play flicker sensory stimulation with an Arduino Uno or NIDAQ BrainWAVE stimulator device. Download Extended Data 1, ZIP file. [file enu-eN-OTM-0257-22-s05.zip › Code_FliCkER/Code_FliCkER/GUI_FliCkER/functions_and_parameters/FlickerMemoryTask/imageset_1a/Set1_177a.jpg]

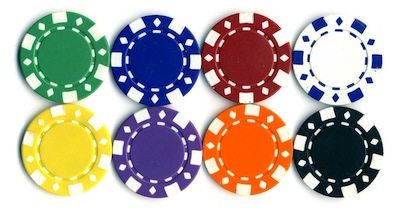

Supplement: Extended Data 1 — BrainWAVE stimulator code. These files contain code to generate and play flicker sensory stimulation with an Arduino Uno or NIDAQ BrainWAVE stimulator device. Download Extended Data 1, ZIP file. [file enu-eN-OTM-0257-22-s05.zip › Code_FliCkER/Code_FliCkER/GUI_FliCkER/functions_and_parameters/FlickerMemoryTask/imageset_1a/Set1_178a.jpg]

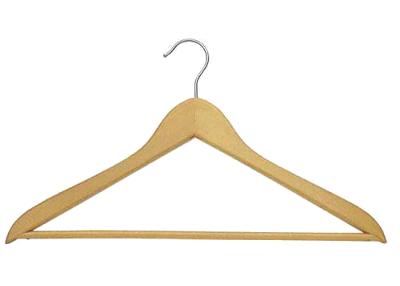

Supplement: Extended Data 1 — BrainWAVE stimulator code. These files contain code to generate and play flicker sensory stimulation with an Arduino Uno or NIDAQ BrainWAVE stimulator device. Download Extended Data 1, ZIP file. [file enu-eN-OTM-0257-22-s05.zip › Code_FliCkER/Code_FliCkER/GUI_FliCkER/functions_and_parameters/FlickerMemoryTask/imageset_1a/Set1_180a.jpg]

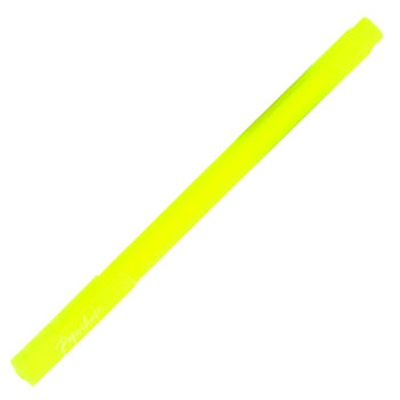

Supplement: Extended Data 1 — BrainWAVE stimulator code. These files contain code to generate and play flicker sensory stimulation with an Arduino Uno or NIDAQ BrainWAVE stimulator device. Download Extended Data 1, ZIP file. [file enu-eN-OTM-0257-22-s05.zip › Code_FliCkER/Code_FliCkER/GUI_FliCkER/functions_and_parameters/FlickerMemoryTask/imageset_1a/Set1_181a.jpg]

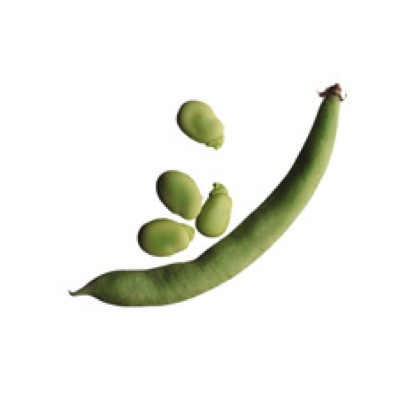

Supplement: Extended Data 1 — BrainWAVE stimulator code. These files contain code to generate and play flicker sensory stimulation with an Arduino Uno or NIDAQ BrainWAVE stimulator device. Download Extended Data 1, ZIP file. [file enu-eN-OTM-0257-22-s05.zip › Code_FliCkER/Code_FliCkER/GUI_FliCkER/functions_and_parameters/FlickerMemoryTask/imageset_1a/Set1_182a.jpg]

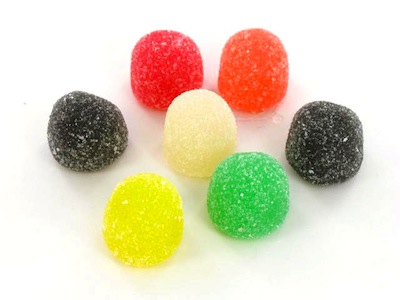

Supplement: Extended Data 1 — BrainWAVE stimulator code. These files contain code to generate and play flicker sensory stimulation with an Arduino Uno or NIDAQ BrainWAVE stimulator device. Download Extended Data 1, ZIP file. [file enu-eN-OTM-0257-22-s05.zip › Code_FliCkER/Code_FliCkER/GUI_FliCkER/functions_and_parameters/FlickerMemoryTask/imageset_1a/Set1_184a.jpg]

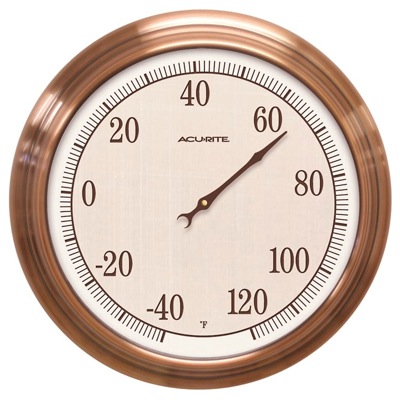

Supplement: Extended Data 1 — BrainWAVE stimulator code. These files contain code to generate and play flicker sensory stimulation with an Arduino Uno or NIDAQ BrainWAVE stimulator device. Download Extended Data 1, ZIP file. [file enu-eN-OTM-0257-22-s05.zip › Code_FliCkER/Code_FliCkER/GUI_FliCkER/functions_and_parameters/FlickerMemoryTask/imageset_1a/Set1_185a.jpg]

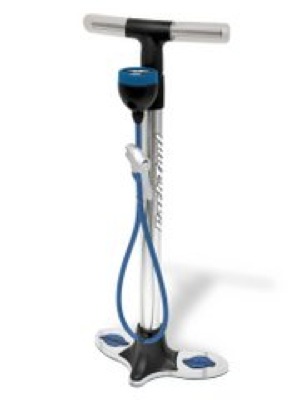

Supplement: Extended Data 1 — BrainWAVE stimulator code. These files contain code to generate and play flicker sensory stimulation with an Arduino Uno or NIDAQ BrainWAVE stimulator device. Download Extended Data 1, ZIP file. [file enu-eN-OTM-0257-22-s05.zip › Code_FliCkER/Code_FliCkER/GUI_FliCkER/functions_and_parameters/FlickerMemoryTask/imageset_1a/Set1_187a.jpg]

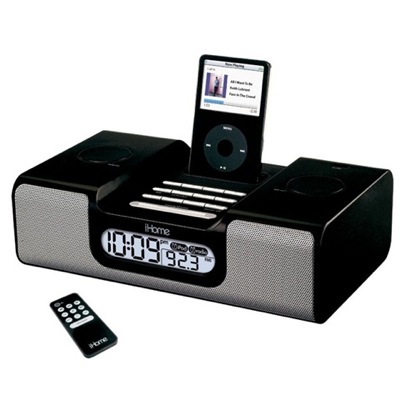

Supplement: Extended Data 1 — BrainWAVE stimulator code. These files contain code to generate and play flicker sensory stimulation with an Arduino Uno or NIDAQ BrainWAVE stimulator device. Download Extended Data 1, ZIP file. [file enu-eN-OTM-0257-22-s05.zip › Code_FliCkER/Code_FliCkER/GUI_FliCkER/functions_and_parameters/FlickerMemoryTask/imageset_1a/Set1_188a.jpg]

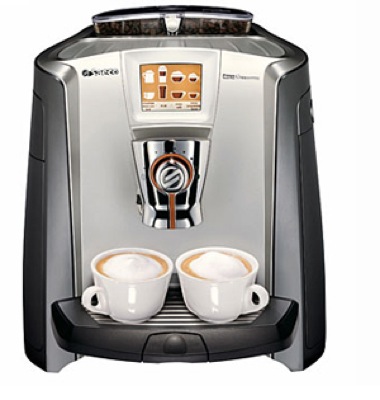

Supplement: Extended Data 1 — BrainWAVE stimulator code. These files contain code to generate and play flicker sensory stimulation with an Arduino Uno or NIDAQ BrainWAVE stimulator device. Download Extended Data 1, ZIP file. [file enu-eN-OTM-0257-22-s05.zip › Code_FliCkER/Code_FliCkER/GUI_FliCkER/functions_and_parameters/FlickerMemoryTask/imageset_1a/Set1_189a.jpg]

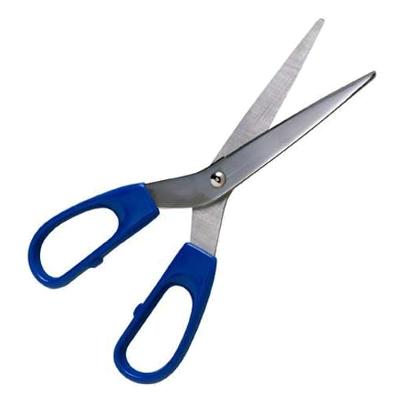

Supplement: Extended Data 1 — BrainWAVE stimulator code. These files contain code to generate and play flicker sensory stimulation with an Arduino Uno or NIDAQ BrainWAVE stimulator device. Download Extended Data 1, ZIP file. [file enu-eN-OTM-0257-22-s05.zip › Code_FliCkER/Code_FliCkER/GUI_FliCkER/functions_and_parameters/FlickerMemoryTask/imageset_1a/Set1_190a.jpg]

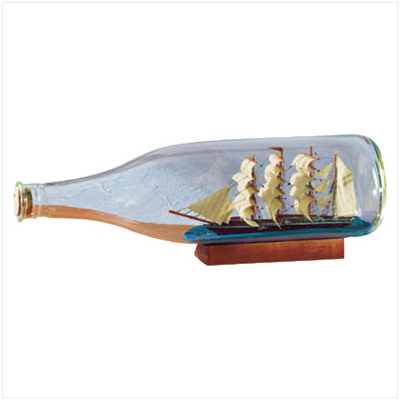

Supplement: Extended Data 1 — BrainWAVE stimulator code. These files contain code to generate and play flicker sensory stimulation with an Arduino Uno or NIDAQ BrainWAVE stimulator device. Download Extended Data 1, ZIP file. [file enu-eN-OTM-0257-22-s05.zip › Code_FliCkER/Code_FliCkER/GUI_FliCkER/functions_and_parameters/FlickerMemoryTask/imageset_1a/Set1_191a.jpg]

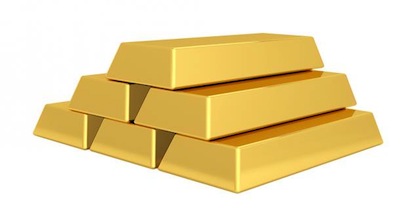

Supplement: Extended Data 1 — BrainWAVE stimulator code. These files contain code to generate and play flicker sensory stimulation with an Arduino Uno or NIDAQ BrainWAVE stimulator device. Download Extended Data 1, ZIP file. [file enu-eN-OTM-0257-22-s05.zip › Code_FliCkER/Code_FliCkER/GUI_FliCkER/functions_and_parameters/FlickerMemoryTask/imageset_1a/Set1_192a.jpg]

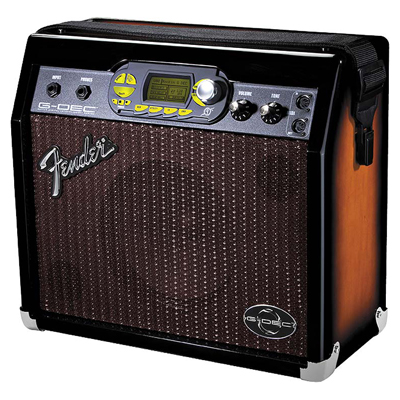

Supplement: Extended Data 1 — BrainWAVE stimulator code. These files contain code to generate and play flicker sensory stimulation with an Arduino Uno or NIDAQ BrainWAVE stimulator device. Download Extended Data 1, ZIP file. [file enu-eN-OTM-0257-22-s05.zip › Code_FliCkER/Code_FliCkER/GUI_FliCkER/functions_and_parameters/FlickerMemoryTask/imageset_1a/Set2_002a.jpg]

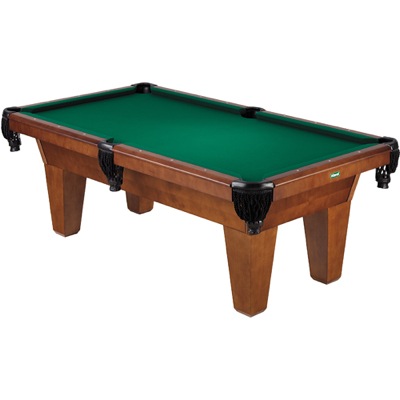

Supplement: Extended Data 1 — BrainWAVE stimulator code. These files contain code to generate and play flicker sensory stimulation with an Arduino Uno or NIDAQ BrainWAVE stimulator device. Download Extended Data 1, ZIP file. [file enu-eN-OTM-0257-22-s05.zip › Code_FliCkER/Code_FliCkER/GUI_FliCkER/functions_and_parameters/FlickerMemoryTask/imageset_1a/Set2_004a.jpg]

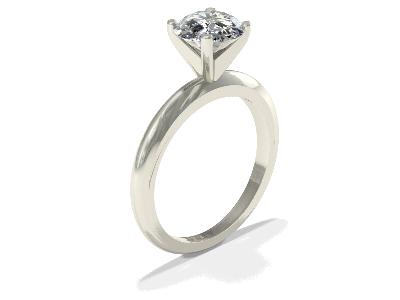

Supplement: Extended Data 1 — BrainWAVE stimulator code. These files contain code to generate and play flicker sensory stimulation with an Arduino Uno or NIDAQ BrainWAVE stimulator device. Download Extended Data 1, ZIP file. [file enu-eN-OTM-0257-22-s05.zip › Code_FliCkER/Code_FliCkER/GUI_FliCkER/functions_and_parameters/FlickerMemoryTask/imageset_1a/Set2_005a.jpg]

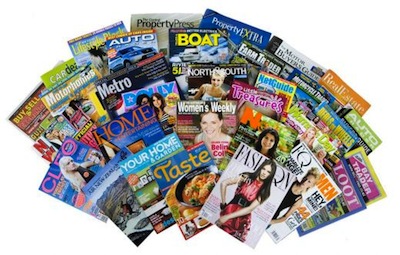

Supplement: Extended Data 1 — BrainWAVE stimulator code. These files contain code to generate and play flicker sensory stimulation with an Arduino Uno or NIDAQ BrainWAVE stimulator device. Download Extended Data 1, ZIP file. [file enu-eN-OTM-0257-22-s05.zip › Code_FliCkER/Code_FliCkER/GUI_FliCkER/functions_and_parameters/FlickerMemoryTask/imageset_1a/Set2_006a.jpg]

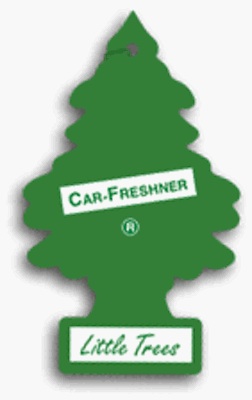

Supplement: Extended Data 1 — BrainWAVE stimulator code. These files contain code to generate and play flicker sensory stimulation with an Arduino Uno or NIDAQ BrainWAVE stimulator device. Download Extended Data 1, ZIP file. [file enu-eN-OTM-0257-22-s05.zip › Code_FliCkER/Code_FliCkER/GUI_FliCkER/functions_and_parameters/FlickerMemoryTask/imageset_1a/Set2_007a.jpg]

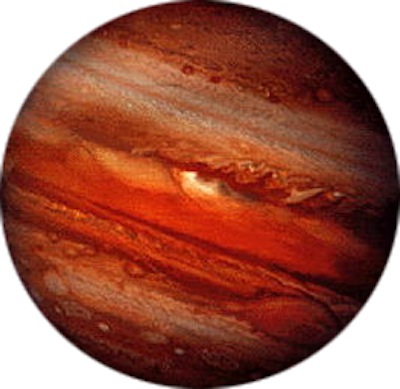

Supplement: Extended Data 1 — BrainWAVE stimulator code. These files contain code to generate and play flicker sensory stimulation with an Arduino Uno or NIDAQ BrainWAVE stimulator device. Download Extended Data 1, ZIP file. [file enu-eN-OTM-0257-22-s05.zip › Code_FliCkER/Code_FliCkER/GUI_FliCkER/functions_and_parameters/FlickerMemoryTask/imageset_1a/Set2_008a.jpg]

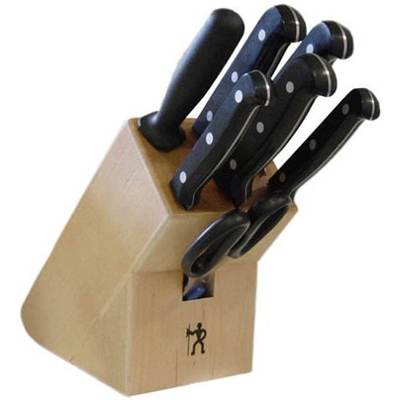

Supplement: Extended Data 1 — BrainWAVE stimulator code. These files contain code to generate and play flicker sensory stimulation with an Arduino Uno or NIDAQ BrainWAVE stimulator device. Download Extended Data 1, ZIP file. [file enu-eN-OTM-0257-22-s05.zip › Code_FliCkER/Code_FliCkER/GUI_FliCkER/functions_and_parameters/FlickerMemoryTask/imageset_1a/Set2_009a.jpg]

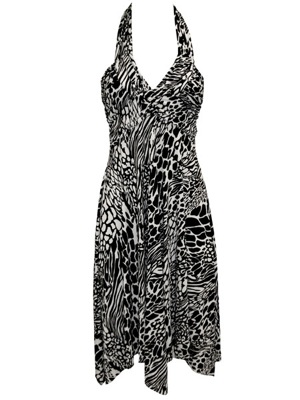

Supplement: Extended Data 1 — BrainWAVE stimulator code. These files contain code to generate and play flicker sensory stimulation with an Arduino Uno or NIDAQ BrainWAVE stimulator device. Download Extended Data 1, ZIP file. [file enu-eN-OTM-0257-22-s05.zip › Code_FliCkER/Code_FliCkER/GUI_FliCkER/functions_and_parameters/FlickerMemoryTask/imageset_1a/Set2_010a.jpg]

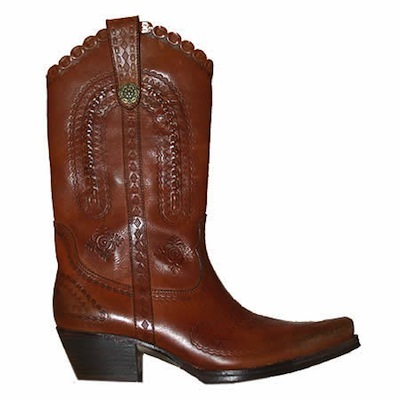

Supplement: Extended Data 1 — BrainWAVE stimulator code. These files contain code to generate and play flicker sensory stimulation with an Arduino Uno or NIDAQ BrainWAVE stimulator device. Download Extended Data 1, ZIP file. [file enu-eN-OTM-0257-22-s05.zip › Code_FliCkER/Code_FliCkER/GUI_FliCkER/functions_and_parameters/FlickerMemoryTask/imageset_1a/Set2_011a.jpg]

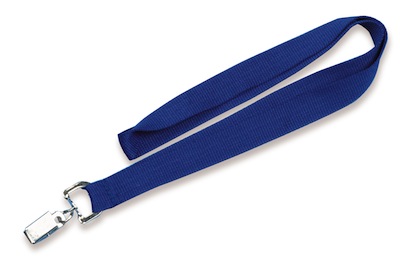

Supplement: Extended Data 1 — BrainWAVE stimulator code. These files contain code to generate and play flicker sensory stimulation with an Arduino Uno or NIDAQ BrainWAVE stimulator device. Download Extended Data 1, ZIP file. [file enu-eN-OTM-0257-22-s05.zip › Code_FliCkER/Code_FliCkER/GUI_FliCkER/functions_and_parameters/FlickerMemoryTask/imageset_1a/Set2_012a.jpg]

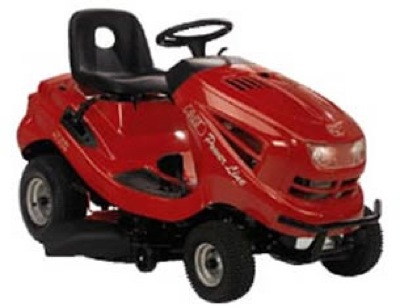

Supplement: Extended Data 1 — BrainWAVE stimulator code. These files contain code to generate and play flicker sensory stimulation with an Arduino Uno or NIDAQ BrainWAVE stimulator device. Download Extended Data 1, ZIP file. [file enu-eN-OTM-0257-22-s05.zip › Code_FliCkER/Code_FliCkER/GUI_FliCkER/functions_and_parameters/FlickerMemoryTask/imageset_1a/Set2_013a.jpg]
