# Supplementary material for: BrainWAVE: A Flexible Method for Noninvasive Stimulation of Brain Rhythms across Species
Source: eNeuro. 2023 Feb 23;10(2):ENEURO.0257-22.2022. doi: 10.1523/ENEURO.0257-22.2022 (PMC9979148; doi:10.1523/ENEURO.0257-22.2022)
Supplement: Extended Data 2 — BrainWAVE stimulator guide. This guide provides more information on how to assemble a BrainWAVE device and set-up the software to produce the stimulation. The guide also provides information on how to measure the stimulation and troubleshooting tips. Download Extended Data 2, RTF file. [file enu-eN-OTM-0257-22-s06.rtf]

BrainWAVE Stimulator Guide


Table of Contents
	Page	
1. Building a BrainWAVE Stimulator	2-4	
2. Detailed Components List	5-6	
3. Physical Diagrams	7-8	
4. Brightness Control	9	
5. Code List	10	
6. GUI_BrainWAVE Software	11-14	
7. Measuring Signals	15	
8. Interpreting & Troubleshooting Signals	16-17	

Building a BrainWAVE Stimulator

When using LEDs of 12 V or greater as visual outputs, the output signal typically required a MOSFET circuit to amplify the signal enough for the LEDs to reach a desired level of brightness. A BrainWAVE MOSFET circuit for visual sensory stimulation includes a breadboard, an N-channel MOSFET, wires and a power source (see detailed parts list on page 5). We used either a DC power supply or another AC/DC wall power adapter as a power source. For experiments sensitive to electrical noise, we recommend batteries as the voltage source for the LEDs.
Components:
Arduino Uno
USB cable (USB type B male to USB type A male) - Typically comes with the Arduino. Battery holder for eight AA batteries
Wires
Solderless breadboard Strip of LEDs
n-channel MOSFET See page 5 for detailed list.

Instructions:
Upload the Flicker Signal Code onto your Arduino Uno:
Install the Arduino IDE software on your PC
Open the 40Hz_Flicker file in the Arduino software.
Plug in your Arduino to the PC via USB type B cable
Click upload (right arrow in circle button)
Confirm that your Arduino Code was successfully uploaded. Lights on Arduino Uno board should flash and IDE will say "Done uploading." at the bottom.

Example of the Arduino IDE and flicker code:


Instructions (cont.):
Assembling the Circuit (See diagram below):
Place your MOSFET into the breadboard so that each of the three prongs are in a different column.
In the image below, the three pongs of the MOSFET are all in row E. The gate, drain and source pins are in columns 8, 9, and 10 respectively. The specific column and rows do not matter as long as the each prong is in a different column.
Connect the ground wire of your battery holder to the breadboard in the same column as the Source (right) pin of the MOSFET
Connect the positive wire of your battery holder to the positive terminal of your LEDs.
Connect the negative end of the LEDs to the breadboard in the same column as the Drain (middle) pin of the MOSFET.
Use a wire to connect a ground pin of the Arduino to the breadboard in the same column as the Source (right) pin of the MOSFET.
Use a wire to connect the signal pin (Pin 12) of your Arduino to the breadboard in the same column as the Gate (left) pin of the MOSFET
Finally, plug the male USB type-B side of the cable into the Arduino. The other end of the USB cable can be connected to a PC or wall adapter

More Physical Diagrams for alternate BrainWAVE circuits can be found on TinkerCAD: [link redacted]


Detailed Components List

Component Name
and Info	Physical Diagram Symbol (TinkerCAD)	Circuit Diagram Symbol	Actual Image	Link to purchase	
n-MOSFET

Though only one is needed, get multiple in case one is or becomes defective.	
	
	
	Bridgold-N-Channel- Transistor- International- Rectifier	
12V DC Voltage Source

Select one of the options below for your 12V DC Voltage Supply can be either:
Eight AA batteries in a holder (recommended)
A 12V wall outlet adapter
Voltage supply	Varies.
See images below.	
	See images below.		
Eight AA Battery Holder

Batteries need to be replaced or recharged, however they will not produce 60Hz noise.	
	
	
	eight 1.5V AA Battery Pack (12V)	
12V ACDC Power Adapter Supply
Plugs into wall outlet.
The 12V adapter has a barrel plug that plugs directly into the Arduino	NA	
	
	https://www.amazon.com
/s?k=12
+volt+acdc+adapter&crid
=
3EHRBGUYKA2UJ&sprefix
=12+acdc+%2Caps% 2C174&ref=nb_sb_ss_i_2_ 8	
Power Supply

This item often found in an electronics/circuits lab. It is very nice, but it is overkill for a simple circuit. It is also large. Turning the voltage too high above 12V can damage your LEDs and/or your Arduino	
	
	
		

Component Name
and Info	Physical Diagram Symbol (TinkerCAD)	Circuit Diagram Symbol	Actual Image	Link to purchase	
Breadboard
Good for building a quick first circuit. Also great for testing and troubleshooting.
Components can easily be inserted or removed. Not as durable as a soldered circuit board.	


	NA	
	Mini
Arduino Shield	
Circuit Board

Can be used instead of a solderless breadboard to create a more durable circuit.		NA	
	https://www.amazon.c om/s? k=PCB&i=industrial&r ef=nb_sb_noss_2	
LED Lights	
	
	
	Superbrightleds.com	
Signal Generator (e.g. NiDAQ or Arduino)
Sends 40Hz signal to circuit and LEDs.
Can use either an Arduino, NI- DAQ, or signal generator.	
	
			
Arduino Uno	
	
	
	https://www.amazon.c om/s? k=arduino+uno&i=ind ustrial&ref=nb_sb_nos s_1	
Wires	
	(lines)	
	circuit wire kit on Amazon	
LED dimmer
In case your LEDs are too bright, a dimmer switch can be used to lower the brightness. See page 8 for more details on using a dimmer.		
	
	https://www.superbrig htleds.com/cat/single- color-dimmer- switches/	

Physical Diagrams

You can tailor your BrainWAVE circuit based on your needs. Below is a physical diagram of an audio and visual flicker BrainWAVE circuit that uses a single speaker to deliver audio stimulation. The circuit also uses a power supply to supply constant voltage to the LEDs instead of a battery pack.
Headphones or ear buds can be used to replace the speaker and an AC/DC power adapter with a barrel plug can be used to replace the power supply.

Physical Diagrams for alternate circuits can be found on TinkerCAD: [link redacted]

Physical Diagrams (cont.)

Simplified LED Flicker circuit:
Uses a function generator to deliver 40Hz Square wave signal
The left oscilloscope is measuring the signal from the function generator. The right oscilloscope is measuring signal that is turning the LEDs on and off.
The LEDs are being powered by a 8-pack of AA batteries which is suppling 12V


Brightness Control
Some ways your circuit may affect the brightness of your LEDs:
Adding a dimmer switch to your circuit
Changing the power being sent to your circuit
This may work, but it is not recommended since LEDs are often designed to be powered at a specific voltage or range of voltages. Powering LEDs at a lower voltage may cause unexpected issues. Powering LEDs at a high voltage may reduce their lifespan.
The brightness of LEDs powered by batteries may decrease as batteries drain over the course of their life span.
Increase the resistance in your circuit using a potentiometer or resistors.
Increasing resistance in your circuit may cause more heat to be produced. A resistor does not change the amount of power that the circuit consumes, instead less of that power is sent to the LEDs and is released as heat.
A heat sink may help mitigate any heating issues.

Product website for dimmer: https://www.superbrightleds.com/moreinfo/single-color-dimmer-switches/ldk-8a-12-24-volt-dc- single-color-led-dimmer/62/365/

Adding a dimmer switch to your circuit:
First disconnect the LEDs from your circuit.
Connect the wires that were connected to the LEDs to the "IN" screw terminals of the dimmer switch.
The wire that is connected to your power source (battery, wall adapter, etc.) should be plugged into IN V+.
The wire that is connected to the drain pin of your MOSFET should be connected to IN V-.
Connected your LEDs to the OUT screw terminals on the dimmer
The positive wire of your LEDs should be plugged into OUT V+.
The ground wire of your LEDs should be plugged into OUT V-.
Tighten all the screw terminal so that the wires will not fall out.


Code List


Code_BrainWAVE Folder\File	Description	
ArduinoUno\_40HzStim_LEDAudio.ino	Runs 40 Hz audio and visual flicker stimulation on an Arduino	
ArduinoUno\RandomStim_LED.ino	Runs a random visual stimulation on an Arduino.	
NIDAQ\visual_audio_stimuli_generation.m	Creates a flicker stimulation files according to user’s parameters	
NIDAQ\playStimuli.m	Reads a flicker stimulation file and sends the signals to a NIDAQ	
GUI_BrainWAVE	This folder contains an app (FlickerMasterTask.mlapp) and its functions and parameters that runs a user-friendly BrainWAVE graphical user interface (GUI) to perform many types of experiments.	

GUI_BrainWAVE Software


Components:
Computer on which the GUI will be run. Computer requirements and software:
Windows platform.
National Instruments Device Monitor software
Matlab 2019b software.
Matlab Data Acquisition Toolbox add-on.
Flicker device, including the following connected components:
Digital acquisition board USB-6212 (National Instruments).
BrainWAVE stimulator circuit.
LED glasses (Mind Alive, Inc).
Earbuds (SONY MDR-EX15LP).
Sleep mask and earplugs (for the occluded part of the experiment).
A lux meter and decibel meter (to measure brightness and volume of stimuli).

Instructions:
Connect the hardware:
Connect the digital acquisition board USB-6212 to the computer.
Connect the digital acquisition board BNC cables for TTL pulses ‘SyncPulse’ and ‘VisualOut’ to the neurophysiology recording system. This setup may differ depending on the recording system used. For the Blackrock NeuroPort system (Blackrock Microsystems, UTSW), one can connect the BNC cables to analog inputs, and set the recording configuration file to record continuously from those channels. More details on the types of TTL pulses:
‘SyncPulse’: square-wave pulse whose duration defines events, including the start of various operations (i.e. the start, pausing, or ending of the experiment), or the start and condition of a trial within the task. The relationship between pulse duration and event identity can be found in the outputted .mat file (more details below).
‘VisualOut’: square-wave pulse which matches the timing and duration of visual pulses presented on the glasses containing LEDs. This pulse provides redundant information, for example, to double-check post hoc the timing and condition of given trials.
Turn on the BrainWAVE stimulator device.

Set up the software
Open the GUI application using Matlab 2019b and on the left side of the GUI:
Enter SubjectID, which is of the format ‘FL###’, where ‘###’ is the subject’s ID number.
Designate the behavioral data output folder by clicking on ‘Change’. The outputted data will be saved under the ‘task-<task name>/sub-<subjectID>’ subfolder. More details on outputted data can be found below. The default output folder is the one where the GUI application resides.
Pick the task to run from the scroll-down menu; tasks include:
FlickerNeurophysTask: involves 15 x 10s trials from various conditions, including visual, auditory, and audio-visual modalities, at 5.5Hz, 40Hz, 80Hz, as well as random stimulation and baseline (no stimulation) conditions.
FlickerFrequencyTask: involves 10 x 10s trials from various conditions, including visual or auditory modalities, at 26 different frequencies from 5.5-80Hz, as well as random stimulation and baseline (no stimulation) conditions.
PulseEvokedPotentialsTask: involves 200 x 1s trials of single pulses of stimulation of the visual, auditory and audiovisual modalities.
One can change some of the default parameters of the task by clicking on ‘Change’. Default
parameters include: light and sound duty cycle of 0.5 (i.e. 50%), sound frequency (i.e. tone) of
7kHz, type of burst ‘ramp_round’ (where the amplitude at the beginning and end of each burst is smoothed so as to avoid clicking noises), data acquisition board sample rate for producing sensory stimuli of 250kH. In case of the PulseEvokedPotentialsTask, the default pulse duration is 12.5ms (to match pulse duration in the 40Hz flicker condition).
Under ‘Hardware Setup’, ‘Platform’ will indicate which computer platform was detected (i.e. Windows or Mac). Note: currently, the data acquisition board API used is only supported on the Windows platform. ‘NIDAQ detected’ will indicate ‘1’ if the data acquisition board is detected, ‘0’ otherwise. If it is not detected, one should ensure the data acquisition board is connected via its USB cable to the computer and then press on ‘Refresh’. The value should now change to ‘1’.
One can click on ‘Test sync pulses’: this will send a short pulse to both the ‘SyncPulse’ and
‘VisualOut’ TTL pulses, so that the experimenter can ensure that they are detected on their
neurophysiological recording system.
Click on ‘Finalized options’: this will disable all functionality on the left side of the GUI, enable functionality on the right side of the GUI, and send a sync pulse to the neurophysiological recording system indicating the start of the experiment.
Note: at any point moving forward, the experimenter can enter notes about the experiment (such as
interruptions, subject’s attentiveness, etc), by typing in the ‘Comment’ box then clicking on ‘Log
comment’. A timestamp of when the experimenter started typing, and what was written, will be saved
in the outputted .log file.

Moving forward, the subject will be exposed to sensory stimuli. At any point, stimuli can be stopped by either pressing on ‘PAUSE’ or ‘STOP’ from within the GUI’s right window, or physically turning off the flicker device (i.e. by switching the ON/OFF button).

Test for comfort:
click on ‘1. Test for Comfort’ – this will open a new window to the right.
The goal of this step is to adjust brightness and volume to levels that are comfortable to the patient. The stimulus used for this purpose will be 40Hz flicker in the case of flicker experiments, or single audio-visual pulses separated by about 1s in the case of the PulseEvokedPotentialsTask.
Ask the subject to put on the glasses and earbuds and click on ‘PLAY…’- this will play the stimulus indefinitely.
Raise the visual and/or auditory levels slowly, until the subject considers those levels to be comfortable.
Click on ‘STOP’ - this will stop the stimulus – then close the right window.
Test for safety:
click on ‘2. Test for Safety’.
The goal of this step is to run through trials of each of the conditions that will be tested during the experiment, and ensure that none of those conditions induce seizure-like symptoms. This may be particularly relevant when running experiments with a population of epilepsy patients, such as with epilepsy patients undergoing intracranial seizure monitoring.
Ask the subject to let the experimenter know if they feel anything unusual at any point during this step. For epilepsy patients, one may specifically ask to lookout for any feelings they might get when they are about to have a seizure, such as their typical auras if they tend to have those.
In the case of FlickerNeurophysTask, press on each condition’s button- this will play a 10s trial of
that condition. In the case of FlickerFrequencyTask, press on the first condition’s button in the top
left- this will play a 10s trial for each of the 27 conditions that will be run.
Run task:
click on ‘3. Run Task’.
This step is the bulk of the experiment, and will present each condition for a planned number of trials, in a pseudo-randomized order, such that no one condition is repeated more than three times in a row. For flicker experiments, this step will last about 1 hour, but every 10 minutes or so the task will automatically be paused so that the subject can take a quick break if desired. For the PulseEvokedPotentialsTask, this step will last about 20 minutes, and there will be no automatic breaks.
Click on ‘START’- this will start running trials.
Once the task is done being run, a pop-up prompt will indicate so.

Occluded condition:
click on ‘4. Test Occluded Condition’.
This step aims to ensure post hoc that we are not detecting noise from the BrainWAVE device, given our experimental setup and preprocessing of the data. It involves occluding sensory stimuli while keeping other experimental variables as close to unchanged as possible, and playing a few sensory stimulation trials.
Ask the patient to wear the sleep mask and earplugs, then place the LED glasses on top of the sleep mask, and earbuds close to the patient’s ears (we’ve found that having the earbuds hang from the glasses’ arms works best).
Click on ‘Run Occluded Condition’.
One this step is done running, the ‘Run Occluded Condition’ button will be disabled.
Measure stimuli intensity levels:
click on ‘5. Measure Brightness and Volume’.
The brightness and volume are tailored to each subject’s comfort. This step ensures that we track
those levels for each subject.
Press on ‘Play 40Hz AV’- this will play a 10s trial of 40Hz audio-visual stimulation. One may need to press this button as needed, depending on how long it takes to take the measurements.
Using the luxmeter, place the detector against the glasses and type in the highest brightness value detected, in the GUI window. We typically obtain 2 measurements (left side and right side of the glasses).
Using the decibel meter, place an earbud against the detector and type in the highest value detected, in the GUI window. Here again, we typically obtain 2 measurements (left-side and right- side earbuds).
Once done measuring, press on ‘Done measuring’.
Close the GUI.
This will send a final ‘SyncPulse’ TTL pulse to the neurophysiological recording system, to indicate
that the experiment is done.

The behavioral data that is saved includes:
‘sub-<subjectID>_stg-raw_task-<task name>_ses-##_desc-<yyyy-mm-dd-hh-mm-ss>_nat-beh.log’: a log file (can be opened with any text editor, or for easier readability with Excel or similar software) containing information about the experiment run, the timing and identity of events, and the timing and content of notes taken by the experimenter.
‘sub-<subjectID>_stg-raw_task-<task name>_ses-##_desc-<yyyy-mm-dd-hh-mm-ss>_nat- beh.mat’: a Matlab file (can be opened with Matlab) containing a structure variable called ‘ExpDetails’, containing information about the experiment run, and details of the stimuli presented. In particular, this variable contains the following 2 important fields:
ExpDetails.SyncPulseCode: 1-column matrix giving relationship between the duration of the ‘SyncPulse’ TTL pulse, and its identity or meaning, with the row number indicating the pulse duration in multiples of 10ms, and the row content indicating the corresponding identity. For example, “Baseline” on row 12 means that sync pulses of duration 120ms indicate the beginning of a baseline (no stimulation) trial.
ExpDetails.Source_Signal: provides details on the types of sensory stimuli that were provided. In particular, this contains matrices with the actual signal outputted by the digital acquisition board for each condition, as well as the order of conditions presented (in ExpDetails.Source_Signal.trials_vector).

Measuring Signals


Measuring Light Output
A photodiode or the analog outputs of a lux meter can be connected to an oscilloscope to measure the frequency and duty cycle of your lights.

To check the frequency of the flicker lights, you can connect the analog output banana terminals to a multimeter or oscilloscope.

The model of lux meter shown above has two BNC analog output ports that can be easily connected to a oscilloscope.

Measuring Audio Output
Two ways you can analyze your audio output include:
Recording the audio and loading the sound file input MATLAB
Connecting a microphone to an oscilloscope to measure the audio in real-time.

A 3.5 mm male to BNC male cable used to connect the audio output of our decibel meter to an oscilloscope:


Interpreting & Troubleshooting Signals


To confirm the accuracy of stimulation and easily diagnose any issues that may have occurred during the building or programming of the device, we measured BrainWAVE Stimulator signals using an oscilloscope.

Common types of atypical signal generator outputs that required further troubleshooting were (1) a signal that produced inaccurate frequencies, (2) asynchronous audio and visual signal outputs when synchronous outputs were desired (Fig. I), and (3) a visual control (input) signal inverted from the visual output signal (Fig. H).

To identify the source of the problem it was important to simultaneously measure audio and visual control signals from the signal generator as well as to measure audio and visual outputs (Fig. F, G).	
	
For the first problem, frequency errors arose from errors within the code used to generate the signal, for example, the amount of time the signal was set to stay on, the delay before the next on- signal, or the temporal precision of the timing signal.

For the second problem, asynchronous light and sound signals have been caused by code that sends output signals in series instead of synchronously, or by sensory modulation or sensory output devices that were not precise enough to transmit the signal without a delay.

The third problem was caused by an error in the visual BrainWAVE Stimulator circuit, where the wires to the gate, drain, and source were connected to the wrong pin of the MOSFET.	
