# Supplementary material for: BrainWAVE: A Flexible Method for Noninvasive Stimulation of Brain Rhythms across Species
Source: eNeuro. 2023 Feb 23;10(2):ENEURO.0257-22.2022. doi: 10.1523/ENEURO.0257-22.2022 (PMC9979148; doi:10.1523/ENEURO.0257-22.2022)
Supplement: Extended Data Table 3-1 — BrainWAVE light and sound levels. These volume for the illuminance of LEDs and the volume of a speaker producing flicker stimulation from have been successfully used in prior studies to comfortably produce γ modulation in humans and mice. Download Table 3-1, DOCX file. [file enu-eN-OTM-0257-22-s04.docx]

***BrainWAVE Light and Sounds Levels***

| Table 2-1. Light and Sound Levels | | |
| --- | --- | --- |
| ***BrainWAVE Light Levels**** | |  |
|  | Mice – LED Strip | 800 lux at side of enclosure near light source  100 lux at side of enclosure far from light source (~6 inches from front of cage) |
|  | Human – Goggles | 100-1000 lux, measured from each eye of the LED goggles with light meter sensor directly on the goggle** |
|  | Human – LED Frame | 500-800 lux, measured at participant’s head position** |
| ***BrainWAVE Audio****** | |  |
|  | Mice – One Speaker | 60-65 dB, measured at animal’s head position |
|  | Human – Earbuds or headphones | 70-80 dB, earbuds, earbuds measured with both earbuds next to decibel meter, headphones measured with decibel meter between headphones** |

*Light measurements were made using a Traceable® Light Meter set to “fast” *while lights flickering at 40 Hz*.

**For human studies, levels may be adjusted to comfort of participant.

***Measured with a dBA decibel meter.
